# Balancing acceleration and turnover in [1 + 1] tetra-imine bis-calix[4]pyrrole reactor for Huisgen cycloadditions

**DOI:** 10.1038/s41467-026-72315-w

**Published:** 2026-04-27

**Authors:** Yifan Li, Gemma Aragay, Pablo Ballester

**Affiliations:** 1 Institute of Chemical Research of Catalonia (ICIQ-CERCA), The Barcelona Institute of Science and Technology (BIST), Avgda. Països Catalans, 16, Tarragona, Spain; 2 ICREA, Passeig Lluís Companys, 23, Barcelona, Spain

**Keywords:** Molecular capsules, Molecular self-assembly

## Abstract

Tailored molecular cages can confine reactive partners and enhance their reaction rates. However, for bimolecular reactions, product inhibition is commonly observed. We report a tetra-imine bis-calix[4]pyrrole cage with two chemically non-equivalent polar hemispheres that promote azide-alkyne Huisgen cycloadditions. This cage forms 1:1 and 1:2 complexes with para-substituted pyridine-*N*-oxides, including ternary hetero-complexes with an azide and an alkyne moiety into proximity. Here, we show that cage confinement accelerates the regioselective formation of 1,4-triazoles. A global kinetic model allows the determination of the intra-vessel rate constant (k_intra_) without direct quantification of the “Michaelis” ternary complex. Modest acceleration is observed for one pair of reactants, yet the cage can still turn over because the product is weakly bound. Extending the azide linker by one methylene dramatically enhances acceleration and introduces product inhibition. Comparisons with a related octa-imine cage reveal how subtle geometric changes tune the balance between transition-state stabilization and product release.

## Abstract (web summary)

Synthetic molecular cages confine substrates within tailored cavities and enhance reaction rates of catalytic reactions but confined bimolecular reactions often suffer from product inhibition due to competition between the products and substrates for binding sites. Here, the authors investigate whether a minimal increase in the cavity length of a bis-calix[4]pyrrole reactor can rebalance confinement-driven acceleration and catalytic turnover while preserving the polar binding motifs.

## Introduction

Enzymes catalyze chemical transformations with high specificity and efficiency by binding substrates in well-defined active sites that preorganize reactive groups and stabilize transition states^1^. Inspired by this function, synthetic molecular cages have emerged as minimalist enzyme mimics^2–4^. Confining substrates within tailored cavities enhances reaction rates by increasing effective local concentrations and enforcing binding geometries resembling the transition state (TS)^5–7^. For bimolecular reactions, the co-binding of the substrates in the so-called “Michaelis” complex converts an intermolecular process into an effectively intramolecular one. Collectively, these are organization effects dominating the catalytic properties of the cages. Polarization/electronic stabilization of the TS and solvation/desolvation effects may also contribute to or even be the main reason for catalytic activity in other systems^8,9^.

Product inhibition is common in both enzymatic and supramolecular catalysis. This occurs when the reaction product competes with the substrates for binding, suppressing catalyst turnover. In biological systems, product inhibition is context-dependent and even regulatory^10^. In supramolecular catalysis, however, it often represents a bottleneck, especially for bimolecular reactions, where entropic factors favor the binding of the product over the binding of two separate substrates. Consequently, synthetic containers are frequently used stoichiometrically or in excess, rather than under truly catalytic conditions.

A few molecular vessels have achieved catalytic turnover in mediating intermolecular cycloadditions. Rebek et al. reported a Diels-Alder (DA) reaction between *p*-benzoquinone and 2,4-dimethylthiophene dioxide inside a self-assembled hydrogen-bonded dimeric capsule capable of turnover^11^. The rate enhancement, expressed as the ratio of half-lives for the encapsulated relative to the background reaction, was modest ( ~10-fold) at 10 mM *p*-benzoquinone and 10 mM diene, while the ternary “Michaelis” complex was undetectable.

More recently, Nau and co-workers described the use of cucurbit[7]uril (CB7) to accelerate the DA dimerization of cyclopentadiene^12^. Adding 10% methanol reduced product binding by more than 10-fold, enabling a TON > 10 for CB7 after 24 h (120 mM cyclopentadiene, 2.5% CB7). The resulting acceleration expressed as kinetic effective molarity (EM_kin_) was estimated to be *~ *10^5 ^M. This value is close to the predicted theoretical maximum ( ~ 10^6 ^M)^13^. Other reported examples of DA reactions showing catalytic turnover rely on fundamentally different mechanisms not involving the formation of a ternary “Michaelis” complex (do not co-confine both substrates). In many of these systems, only one substrate is included within the molecular cavity, or the reaction proceeds outside the cavity, and the resulting catalytic effect is mainly ascribed to substrate polarization and electronic stabilization of the TS, rather than to bimolecular pre-organization within a confined environment^14–17^. Because these approaches operate through a distinct mechanism, they are not directly relevant for comparison with the present investigations. 

For Huisgen cycloadditions, Mock and co-workers showed that cucurbit[6]uril (CB6) accelerates reactions between ammonium-derived azides and alkynes and enforces regiospecific formation of 1,4-triazole^18,19^. They noted that product release was feasible in some cases, demonstrating the container’s capacity for turnover; however, it may become rate-limiting for others. The reported acceleration was approximately 10^5^, based on the ratio of the bimolecular reaction rate constants in the container and in the bulk (k_bulk_). Because the Michaelis complex was not detected, the bimolecular rate constant within CB6 had to be inferred indirectly from kinetic and thermodynamic constant values derived from least-squares fitting of kinetic data. Other reported container-mediated Huisgen cycloadditions exhibited product inhibition^20^.

**Fig. 1:**
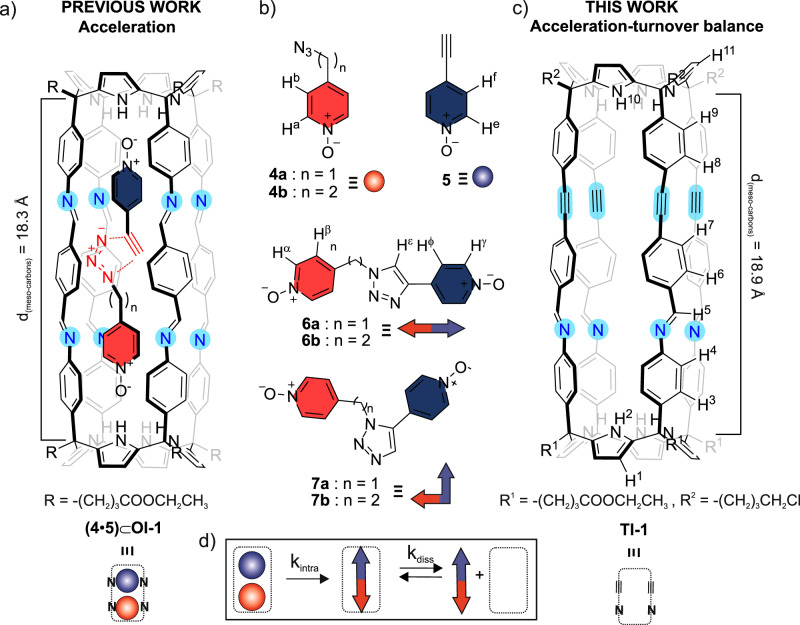
Chemical structures and schematic representations of the cages, substrates, and cycloaddition products. **a** Ternary “Michaelis” complex of the previously reported octa-imine cage **OI-1**; (**b**) *para*-substituted pyridine-*N*-oxide substrates **4a**/**4b** and **5** used in this work, and the corresponding disubstituted triazole products (**6**/**7**, *n* = 1 or 2); (**c**) structure of the tetra-imine cage **TI-1** reported here; (**d**) schematic representation of the intra-vessel 1,3-dipolar cycloaddition reaction (k_intra_) and the product release equilibrium (k_diss_)^23^.

We previously developed poly-imine bis-calix[4]pyrrole cages with polar cavities and tunable sizes (Fig. 1)^21,22^, which mediate Huisgen^23,24^ and DA^25,26^ reactions with high regioselectivity and large EM_kin_ values (10^3 ^M). In the octa-imine cage **OI-1**, two converging polar binding sites strongly bind *para*-substituted pyridine-*N*-oxide and 2-pyridinone derivatives, suitable for Huisgen cycloadditions, and organize their reactive ends in face-to-face arrangements resembling, in the 1:1:1 “*Michaelis*” complex, the bulk transition state (TS) geometry of the 1,4-triazole, accounting for both acceleration (primarily entropic) and regioselectivity. We showed through Eyring analysis that the observed rate acceleration arose mainly from a reduction in the entropic cost of the bimolecular process, consistent with the cage acting as an “entropic trap”.

Here, we ask whether a minimal increase in the cavity length, while preserving the polar binding motifs, can rebalance confinement-driven acceleration and catalytic turnover in bis-calix[4]pyrrole reactors. To address this question, we report the self-assembly of a [1 + 1] bis-calix[4]pyrrole tetra-imine cage, **TI-1** (Fig. 1), obtained by replacing four imines (C-N = C-C, 3.7 Å) in **OI-1** with alkynes (C-C ≡ C-C, 4.3 Å), thereby elongating the cavity by ~ 0.6 Å while retaining the cylindrical architecture and the inwardly directed polar groups. We then quantify the binding and the kinetics of the Huisgen cycloadditions of azides **4a**/**4b** with alkyne **5** within the cavity of **TI-1**.

We show that bis-calix[4]pyrrole poly-imine cages are highly editable reactor vessels. In moving from **OI-1** to **TI-1**, the sub-Å cavity elongation provides a direct means to balance confinement-driven entropy-based acceleration with product release, enabling catalytic turnover previously unattained in our systems.

**TI-1** is a unique molecular vessel that uses strong, directional interactions to orient both substrates and products while still enabling turnover in the reaction of **4a** with **5** within its cavity. Finally, moving beyond empirical rate comparisons common in earlier studies, the use of a global kinetic model yields k_intra_ and the corresponding effective molarity (EM_k__in_) as robust quantitative descriptors providing an ideal framework for rationalizing confined catalysis.

## Results

### Synthesis of pyridine-*N*-oxide derivatives

The pyridine-*N*-oxide derivatives **4a,** **4b,** **5,** **6a**, and **6b** were prepared following previously described procedures^23^.

### Synthesis of precursors for the self-assembly of cage TI-1

The tetra-amine tetra-ester aryl-extended calix[4]pyrrole AE-C[4]P **3** was prepared following a reported procedure^27^.

The tetra-formyl tetra-chloro super-aryl extended calix[4]pyrrole SAE-C[4]P **2** was synthesized via a quadruple Sonogashira cross-coupling reaction and isolated as a yellow solid in 60% yield after chromatography and recrystallization (see SI for details). Its ^1^H NMR spectrum is broadened in chlorinated solvents, most likely due to aggregation, whereas the spectrum in DMF-*d*_7_ showed sharp signals for all protons (see Supplementary Figs. S2, S3), consistent with *C*_4v_ symmetry (formyl singlet at δ = 10.1 ppm; see SI for further details).

**Fig. 2:**
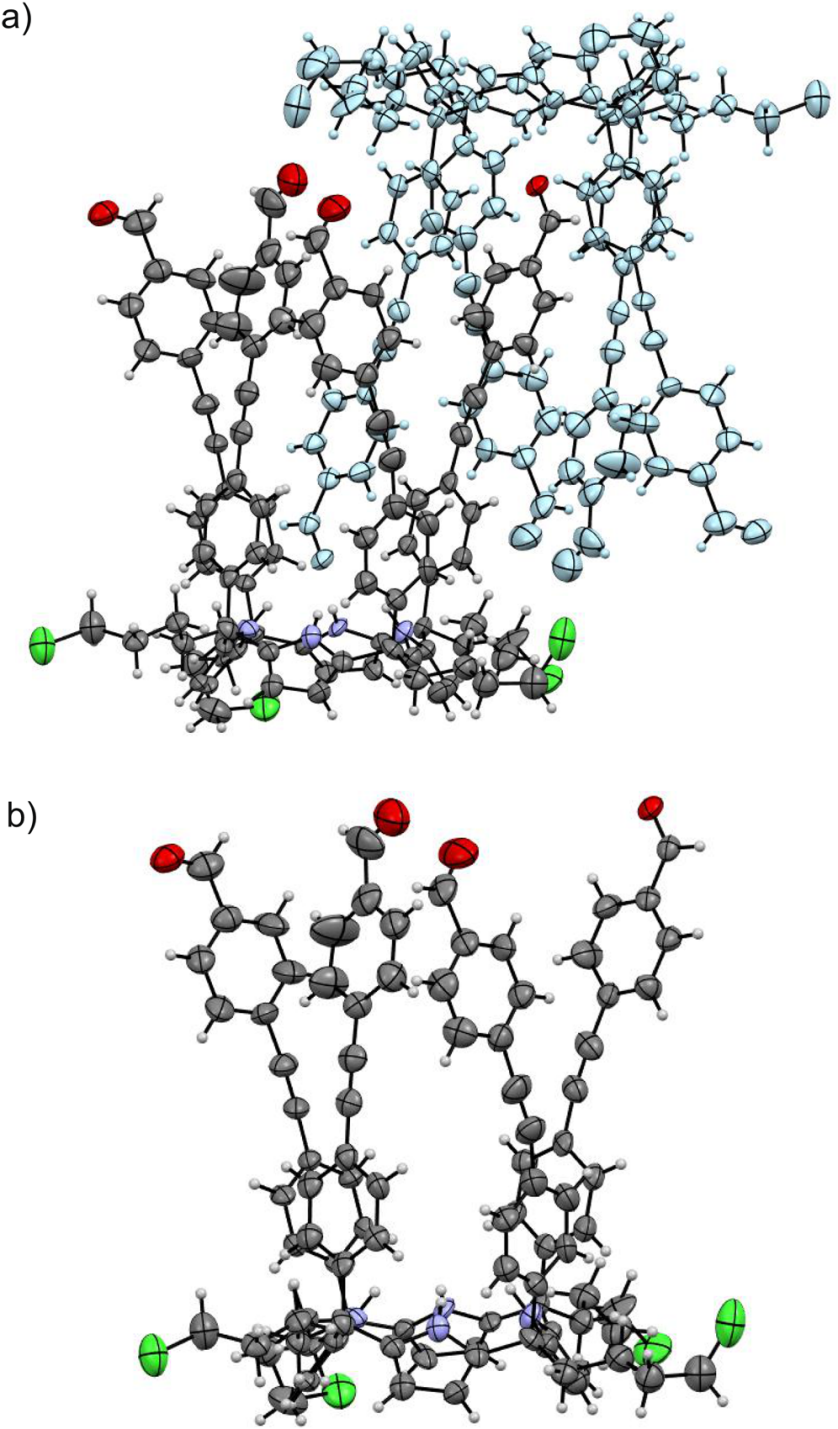
X-ray structure of SAE-C[4]P 2. Side views of the X-ray structure of tetra-formyl *meso*-tetra-4-chlorobutyl-tetra-*p*-(ethynyl-benzaldehyde)phenyl calix[4]pyrrole, SAE-C[4]P **2**. Panel (**a**) shows the dimeric structure present in the crystal packing, and panel (**b**) shows the asymmetric unit cell. The structures are shown as ORTEP representations with thermal ellipsoids set at 50% probability for the non-hydrogen atoms. Hydrogen atoms are depicted as fixed-size spheres with a radius of 0.15 Å.

X-ray diffraction revealed that SAE-C[4]P **2** crystallizes as an interpenetrated dimer (Fig. 2), in which the inclusion of an aromatic *meso-*aryl substituent from the adjacent monomer and formyl oxygen···HN pyrrole hydrogen bonding stabilizes the cone conformation. This packing motif supports monomer-dimer equilibrium in non-polar solvents, in agreement with the observed broadening of proton signals.

### Self-assembly of tetra-imine cage TI-1

**Fig. 3:**
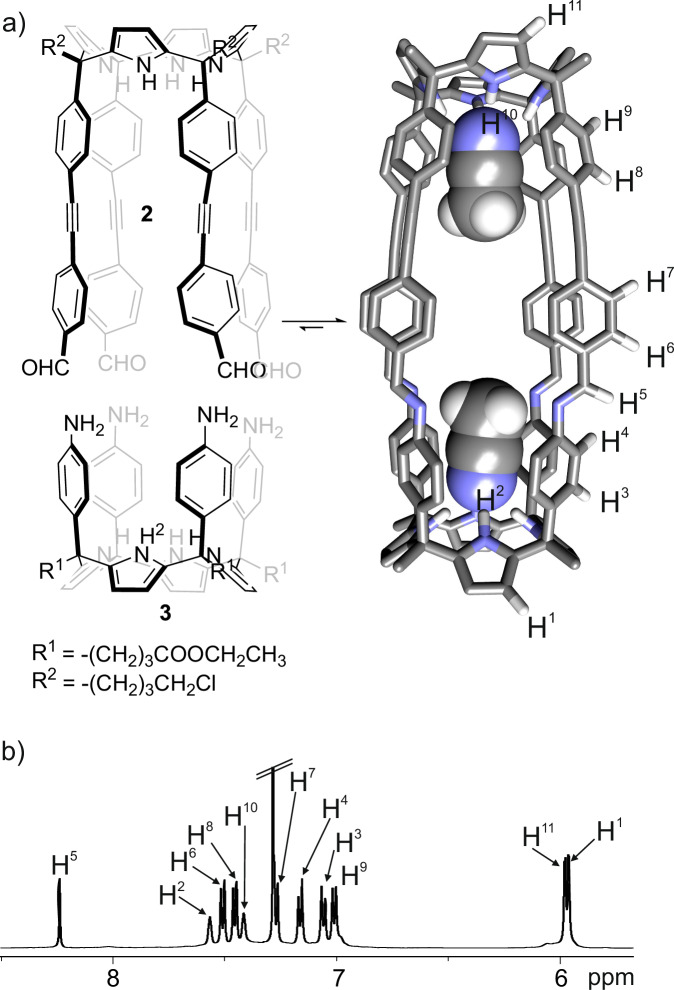
Synthesis and characterization of TI-1. **a** Synthetic scheme for the reversible self-assembly of tetra-imine cage **TI-1**. The DFT energy-optimized structure **TI-1**, incorporating two acetonitrile molecules within its polar cavity, is shown. The cage structure is shown in stick representation, whereas the included CH_3_CN molecules are depicted as CPK models. Non-polar hydrogen atoms are omitted for clarity, except those used for proton assignment. **b** Partial ^1^H NMR spectrum (500 MHz, 298 K) of **TI-1** recorded in a CDCl_3_:CD_3_CN 9:1 solvent mixture, with proton assignment corresponding to cage **TI-1** in (**a**).

Self-assembly of **TI-1** was monitored using ^1^H NMR spectroscopy from an equimolar mixture of SAE-C[4]P **2** and AE-C[4]P **3** (2 mM) in CDCl_3_:CD_3_CN (9:1) with 0.05% acetic acid (Fig. 3). After 4 days at room temperature, all proton signals of the precursors disappeared, and a new set of sharp signals assigned to **TI-1** appeared (Fig. 3). Using 1,3,5-trimethoxybenzene as an internal standard (i.s.), we quantified the yield to be > 90%. The slower self-assembly of **TI-1** relative to a smaller analog^22^ is attributed to the aggregation/dimerization equilibria of SAE-C[4]P **2** that reduce the free monomer concentration. The ^1^H NMR spectrum of **TI-1** showed two broad singlets resonating at δ = 7.38 and δ = 7.55 ppm, attributed to the pyrrole NHs of the two chemically non-equivalent hemispheres. The diagnostic imine-proton, H^5^, appeared as a sharp singlet at δ = 8.25 ppm. The protons of the cage aromatic walls, H^3^, H^4^, H^6^, H^7^, H^8^, and H^9^, resonated as six distinct *ortho*-coupled doublets between δ = 7.0 and 7.5 ppm (Fig. 3a, b).

The β-pyrrole protons in the two hemispheres appeared as two separate doublets centered at approximately δ = 5.89 ppm. **TI-1** was characterized using a complete set of high-resolution spectra (NMR, HRMS, and DOSY D = 4.0 × 10^−10^ m^2^·s^−1^, see SI). A preparative-scale self-assembly of **TI-1** (40 mg), followed by MeOH-induced precipitation from CH_2_Cl_2_, afforded **TI-1** as a yellow solid in ~80% yield for subsequent studies. 

### Binding studies of TI-1 cage with pyridine-*N*-oxide derivatives

Before kinetic measurements, we characterized the binding of **TI-1** toward monotopic substrates (azides **4a**/**4b**), and alkyne **5**) and ditopic 1,4-triazole products (**6a**/**6b**) in CDCl_3_:CD_3_CN (9:1) using NMR spectroscopy. Because **TI-1** has two chemically non-equivalent hemispheres (AE and SAE), 1:1 inclusion typically affords two regioisomeric complexes, while higher guest loadings yield 2:1 complexes.

### Binding of 4-ethynyl pyridine-*N*-oxide 5 with TI-1

**Fig. 4:**
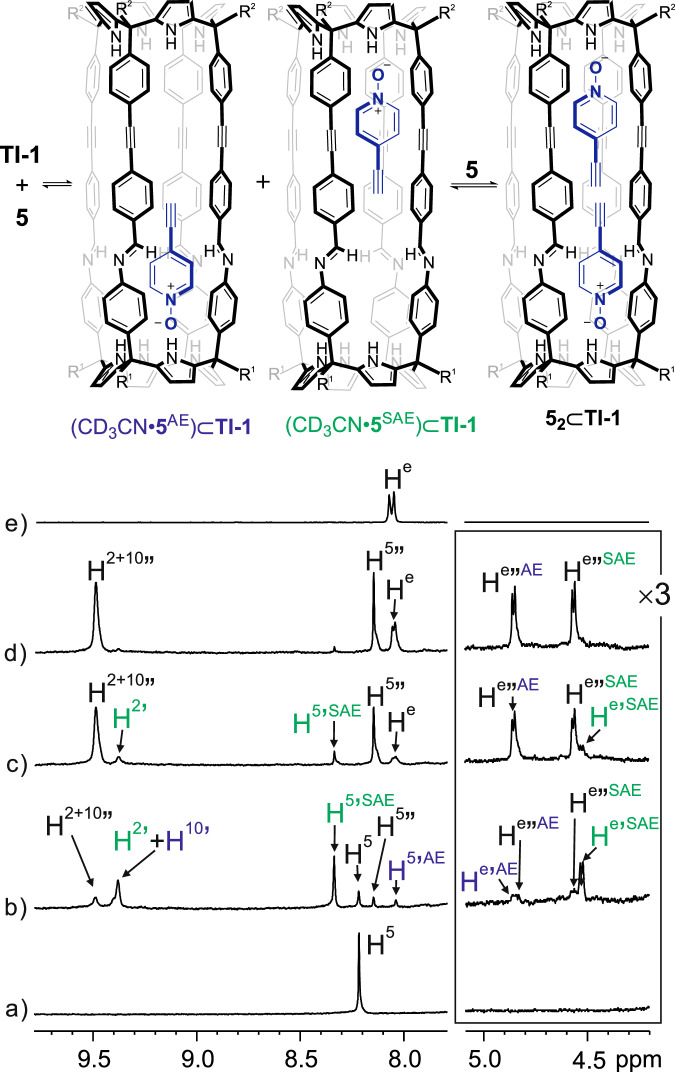
Binding of 5 to TI-1. Top) Equilibria for the formation of 1:1 regioisomers [(CD_3_CN•**5**^SAE^)⊂**TI-1**, (CD_3_CN•**5**^AE^)⊂**TI-1**] and **5**_**2**_⊂**TI-1**. Bottom) Partial ^1^H NMR spectra for the titration (500 MHz, at 298 K, CDCl_3_:CD_3_CN 9:1) of **TI-1** (2 mM) with **5**: (**a**) 0 equiv, (**b**) 1 equiv, (**c**) 2 equiv, (**d**) 3 equiv; spectrum (**e**) shows free **5**. Primed signals correspond to 1:1 and double-primed signals to 2:1 complexes; the host and guest signals in (CD_3_CN•**5**^SAE^)⊂**TI-1** and (CD_3_CN•**5**^AE^)⊂**TI-1** are highlighted in green (SAE) and blue (AE), respectively. See Figs. 1, 3 for the proton assignments.

In CDCl_3_:CD_3_CN (9:1), adding 1 equiv of **5** to **TI-1** (2 mM) generates four imine-H^5^ signals (Fig. 4b): free **TI-1** (δ = 8.22 ppm, 19%), two 1:1 isomers **5**⊂**TI-1** (δ = 8.34 ppm, 54%, and δ = 8.02 ppm, 13%), and the 2:1 complex **5**_**2**_⊂**TI-1** (δ = 8.14 ppm, 14%).

2D-NOESY assigns the major 1:1 isomer (54%) to (CD_3_CN•**5**^SAE^)⊂**TI-1**, corresponding to **5** bound in the SAE-hemisphere with a co-included CH_3_CN molecule in the opposite pole (see SI). The constant isomer ratio from the outset indicates rapid equilibration and thermodynamic control of binding regioselectivity. The (CD_3_CN•**5**^SAE^)⊂**TI-1** isomer is favored by 0.8 kcal/mol. Diagnostic complexation-induced shifts include the bound pyrrole NHs moving upfield (δ ~ 9.5 ppm) relative to free **TI-1**, consistent with HN⋯O(*N*-oxide) hydrogen bonding. The bound NHs of **5**_**2**_⊂**TI-1** appear as the most downfield shifted low intensity signal, whereas those of the two **5**⊂**TI-1** regioisomers overlap into a more intense one. No signals are observed for free **5**.

The aromatic *alpha*-pyridyl-*N*-oxide protons in **5**_**2**_⊂**TI-1** complex are upfield-shifted, resonating as two separate *ortho*-coupled doublets of equal but very reduced intensity (δ = 4.6 and 4.9 ppm, *Δ*δ = 3.5 and 3.2 ppm, respectively). Two additional *ortho*-coupled doublets with unequal intensities appear in the same region: the more intense, more upfield belongs to (CD_3_CN•**5**^SAE^)⊂**TI-1**, and the lower-intensity to (CD_3_CN•**5**^AE^)⊂**TI-1**.

The complexation-induced shifts (CIS) of the aromatic *alpha* protons of bound **5** in the 1:1 regioisomers serve as a reference for the assignment of the corresponding proton signals of the two pyridine-*N*-oxide copies in **5**_**2**_⊂**TI-1**. 

Upon adding more than 1 equiv of **5**, the signals of **5**_**2**_⊂**TI-1** increase at the expense of those of the 1:1 regioisomers (Fig. 4c). With slightly more than 2 equiv of **5**, only the signals of **5**_2_⊂**TI-1** and free **5** remain (Fig. 4d). Taken together, these observations indicate that the inclusion of **5** into **TI-1** is fast on the human timescale (less than 60 s), the stepwise binding constants for forming **5**_2_⊂**TI-1** exceed 10^4 ^M^−1^, and the binding process exhibits weak negative allosteric cooperativity. Free **TI-1** and its complexes are in slow chemical exchange on both the proton chemical-shift and EXSY time scales (*k*_off_ < 0.01 s^−1^).

### Binding of 4-azido(alkyl)-pyridine-N-oxides 4a/4b with TI-1

**Fig. 5:**
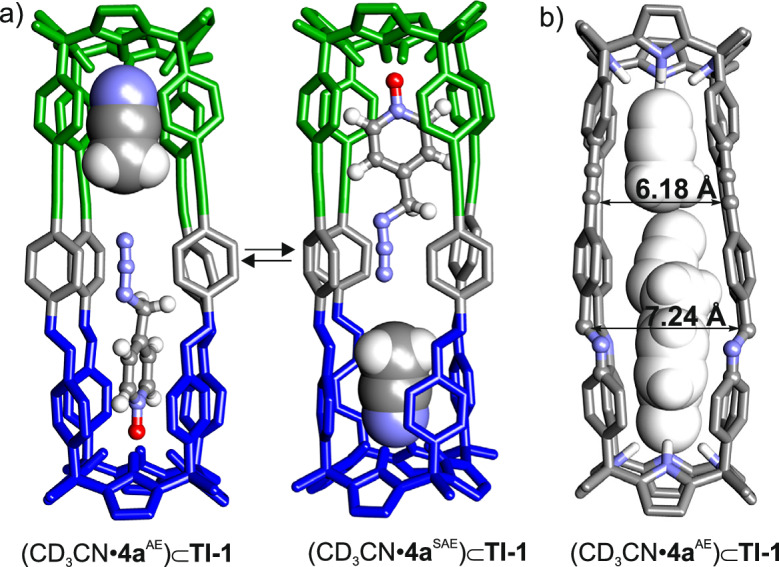
Regioisomers of (CD_3_CN)•4a⊂TI-1 and exchange mechanism. **a** Isomerization equilibrium between kinetic and thermodynamically favored **4a**⊂**TI-1** regioisomers: (AE/SAE)_kinetic_ ≈ 4:1 to (AE/SAE)_thermodynamic_ ≈ 1:4 over several hours. **b** Putative TS of “french-doors” guest-exchange model showing distinct AE and SAE portals; labeled distances indicate a difference of ~ 1 Å between the two openings. The adjacent *meso*-phenyls adopt an almost parallel orientation in the TS.

Azides **4a**/**4b** show binding patterns analogous to **5** (two 1:1 regioisomers plus **4**₂⊂**TI-1** at higher loading), but with a distinctive time dependence in the 1:1 isomer ratio. Immediately after mixing **TI-1** with 1 equiv of **4**, the kinetic distribution is dominated by the (CD_3_CN•**4**^AE^)⊂**TI-1** isomer (AE/SAE ≈ 4:1). Over ~ 4 h, the ratio relaxes to the thermodynamic value AE/SAE ≈ 1:4, establishing that for G = **4** or **5** the (CD_3_CN•**G**^SAE^)⊂**TI-1** isomer is consistently favored by ~ 0.8 kcal·mol⁻¹ (Fig. 5a). The longer time required to reach equilibrium in the case of **4**⊂**TI-1** suggested an increase in its kinetic stability (*k*_off_ ≤ 0.002 s^−1^, see Supplementary Figs. S13 and S14).

In short, the thermodynamic regioselectivity of 1:1 complexes is uniform across guests, whereas the kinetic selectivity depends on the guest structure. We attribute this behavior to the existence of two independent “french-doors” exchange processes operating at the **TI-1** portals^28^. The portal opening at the AE binding site is slightly larger in the exchange transition state (Fig. 5b), lowering the barrier for ingress/egress at this site. Bulkier azides **4** therefore enter preferentially via the AE site under kinetic control, but the kinetically favored (CD_3_CN•**4**^AE^)⊂**TI-1** converts slowly to the thermodynamically preferred SAE isomer via slow dissociation (k_off_ <0.002 s⁻¹).  The less bulky alkyne **5** exhibits faster exchange, and the kinetic and thermodynamic preferences for the (CD_3_CN•**5**^SAE^)⊂**TI-1** isomer coincide. Hence, the mixture of 1:1 isomers for **5** results from thermodynamic control of binding from the beginning. Given the similar thermodynamic stabilities across the (CD_3_CN•**G**)⊂**TI-1** complexes (see ITC section), their kinetic stabilities (*K* =k_on_/k_off_) are expected to be of the same order of magnitude, which argues against a markedly faster dissociation of any putative (CD_3_CN•**5**^AE^)⊂**TI-1** kinetic isomer.

Upon adding more than 1 equiv of **4**, signals of **4**₂⊂**TI-1** increase at the expense of those of the 1:1 complexes; with 2 equiv, **4**₂⊂**TI-1** dominates, with a minor residual 1:1 and free guest.

### Binding of TI-1 with 1,4-disubstituted triazole cycloaddition products 6a and 6b

Because **6a**/**6b** are sparingly soluble in CDCl_3_:CD_3_CN (9:1), we used reverse titrations (adding **TI-1** to solutions of **6**). For **6a,** **TI-1** addition (0.5 equiv) produces two sharp sets of **6a**⊂**TI-1** signals in an initial 4:1 ratio that slowly evolves to ~1:1 over ~4 days, while free **6a** remains present and no free **TI-1** is detected. These observations indicate (i) strong binding (K > 10⁴ M⁻¹), (ii) slow exchange on the chemical-shift time scale, and (iii) slow interconversion between the two regioisomeric complexes. We denote **6a**^N-SAE^⊂**TI-1** as the kinetic isomer (azide-derived terminus in the SAE hemisphere), consistent with preferential passage of the less bulky *N*-oxide end (ethynyl-derived pyridine-*N*-oxide) through the larger portal opening at the AE binding site. The ditopic complexes are also kinetically more stable than the monotopic ones (k_off_ <0.001 s⁻¹ *vs*. ~ 10⁻²–10⁻³ s⁻¹, estimated using simulation function in COPASI software). This is consistent with the equilibration kinetics of the **6a**⊂**TI-1** regioisomers, which took several days.  Notably, the CIS for the pyrrole-NHs in **6a**⊂**TI-1** are about half those of monotopic complexes (Δδ(NH) ≈ 1 ppm *vs*. ≈ 2 ppm), suggesting a length mismatch: **6a** cannot optimally span both polar sites of **TI-1**.

In contrast, **6b** forms two regioisomeric complexes in a persistent SAE:AE ~ 9:1 ratio with no detectable equilibration over two weeks, indicating greater kinetic stability and possibly stronger binding. The larger NH CIS for **6b**⊂**TI-1** (Δδ(NH) = 2.2–2.5 ppm) supports a better geometric fit and shorter HN⋯O interactions than in **6a**⊂**TI-1**.

### Displacement of the ditopically bound guests in 6⊂TI-1 complexes by adding an excess of monotopic alkyne 5

To probe product release, we exposed **6a**⊂**TI-1** to an excess of **5**. Starting from an equimolar **TI-1**/**6a** mixture (2 mM), addition of 1 equiv of **5** produced after 2 h (CD_3_CN·**5**)⊂**TI-1** ( < 5%) and **5**₂⊂**TI-1** ( ~ 25%), accompanied by a drop in **6a**⊂**TI-1** ( ~ 70%) and the appearance of free **6a**. With 4 equiv of **5**, the extent of displacement increased, confirming that **6a** can be replaced by monotopic substrates and reach equilibrium on a several-hour time scale, consistent with k_off_ < 0.001 s⁻¹. We simulated these competition experiments (HySS2009), fixing the stability constants for (CD_3_CN·**5**)⊂**TI-1** and **5**_2_⊂**TI-1** to the ITC-derived values and optimizing K(**6a**⊂**TI-1**) to reproduce the speciation profiles, giving K ≈ 3 × 10⁵ M⁻¹ (Supplementary Fig. S27). We estimated the thermodynamic effective molarity as EM_thermo_(**6a**⊂**TI-1**) = K(**6a**⊂**TI-1**)/K(**5**_2_⊂**TI-1**) ≈ 3 × 10⁻³ M, indicating that turnover is feasible at millimolar substrate concentrations (the binding constant of **5**_**2**_**⊂T1-1** is arbitrarily used as reference instead of K_ref_^2^ for the binding of **5** to a binding site model). In contrast, analogous experiments with **6b** showed no detectable displacement, consistent with very strong binding, anticipating product inhibition for reactions producing **6b**. Theoretical speciation analysis (Hyss2009) indicated that the stability constant of **6b**⊂**TI-1** exceeds 10^7 ^M^-1^ (EM_thermo_(**6b**⊂**TI-1**) > 0.1 M, see Supplementary Fig. S33). 

### Isothermal Titration Calorimetry (ITC) experiments

We used ITC to determine binding thermodynamics for the formation of the 1:1 and 2:1 complexes of **TI-1** with **4a,** **4b**, and **5** in a CHCl_3_:CH_3_CN (9:1). Incremental addition of each *N*-oxide to a tenfold-diluted **TI-1** solution produced single sigmoidal binding isotherms (Supplementary Figs. S16, S18, S20), indicating negligible binding cooperativity. Fits to the “one set of sites” model (MicroCal) yielded intrinsic (statistically corrected) binding constants (Table 1).  The stoichiometry parameter *n* converged to *n* ~ 2 for alkyne **5**, as expected for two nearly equivalent sites, whereas *n* ~ 1.4-1.6 for azides **4**. We attributed these low *n* values to the unusually slow dissociation (as shown above) that compromises equilibration during injections^29^. The intrinsic binding constants are consistent with estimates from the ^1^H NMR titration experiments. Overall, ITC confirms that azides **4a/4b** bind slightly more strongly than **5**, and that binding is enthalpy-driven, with a reduced unfavorable entropic term, supporting significant solvation/desolvation contributions.

**Table 1:** Intrinsic binding constant (K, M^-1^), and binding enthalpy and entropy terms (ΔH and TΔS, kcal·mol^-1^) for the interactions of the monotopic pyridine-*N*-oxides 4a, 4b, and 5 with TI-1

Guest	K_intrinsic_×10^-4^ (M^-1^)^a,b^	ΔH(kcal·mol^-1^)	TΔS(kcal·mol^-1^)
**4a**	4.4 ± 1.0	− 9.1 ± 0.1	− 2.7 ± 0.2
**4b**	1.8 ± 0.5	− 7.3 ± 0.1	− 1.6 ± 0.2
**5**	0.9 ± 0.1	−6.9 ± 0.5	− 1.5 ± 0.5

a The two binding sites are assumed to be identical and independent. The intrinsic binding constants are related to the stepwise and overall thermodynamic constants of the (CD_3_CN•**G**)⊂**TI-1** and **G**_2_⊂**TI-1** complexes as follows: K_1:1_ = 2 × K_intrinsic_; K_1:1 ⇌ 2:1_ = K_intrinsic_ / 2; β_2:1_ = K_intrinsic_^2^. b It is worth noting that the broad shape of the heat release peaks suggests slow binding kinetics and complicates fitting the data to standard binding models. We used long equilibration times between injections to allow complete equilibration.

We attempted analogous ITC measurements for ditopic **6a** and **6b**, but the very slow dissociation of the formed complexes relative to the calorimeter equilibration time scale resulted in broad, non-ideal heat-release profiles indicative of kinetically limited binding^29^. As a result, standard fitting underestimated affinities, and we rely on competitive ^1^H NMR titration experiments for robust K values (see “displacement experiments” section above).

### Kinetic studies of the 1,3-dipolar cycloaddition reactions of 4a and 4b with 5 in the cavity of TI-1. Stoichiometric conditions

Mixing equimolar **4a,** **5**, and **TI-1** (2 mM each) in CDCl_3_:CD_3_CN (9:1) produces a ^1^H NMR spectrum showing a multispecies mixture, including the known **4a**⊂**TI-1** and **4a**₂⊂**TI-1** complexes and a new imine signal (δ = 8.22 ppm) assigned to the ternary Michaelis complex (**4a**·**5**)⊂**TI-1** (likely as two regioisomers with overlapping H^5^ signals)(Supplementary Fig. S34). Speciation simulations (HySS2009) using the measured binding constants are consistent with the experimental mixture when assigning β((**4a**·**5**)⊂**TI-1**) = 2.0 × 10⁸ M⁻², giving [(**4a**·**5**)⊂**TI-1**] ≈ 0.34 mM under these conditions (Supplementary Fig. S35).

Reaction progress monitored by ^1^H NMR shows the growth of diagnostic signals for the two **6a**⊂**TI-1** regioisomers (1:1), with concomitant decay of substrate-bound species.  It is worth noting that the half-life of the bimolecular reaction between any of the two azides **4** and the alkyne **5** in the bulk at 2 mM is 283 years. Thus, the observation of proton signals of the corresponding cycloaddition triazole products, **6a** and **6b**, or their **TI-1** complexes in the ^1^H NMR spectra must arise from substantial acceleration within **TI-1**. After 70 days, **6a**⊂**TI-1** dominates. From initial rates, the acceleration of the confined reaction relative to bulk is quantified by EM_kin_ ≈ 45 M (k_intra_/k_bulk_ = 2.5 × 10⁻⁶ s⁻¹ / 5.6 × 10⁻⁸ M⁻¹ s⁻¹).

For the analogous cycloaddition reaction between **4b** and **5** in the presence of 1 equiv of **TI-1**, the ^1^H NMR spectrum showed exclusively the signals of **6b**⊂**TI-1** just after 3 h (Supplementary Fig. S47). The reaction within **TI-1** is too fast for accurate EM_kin_ characterization using the initial rates method.

**Fig. 6:**
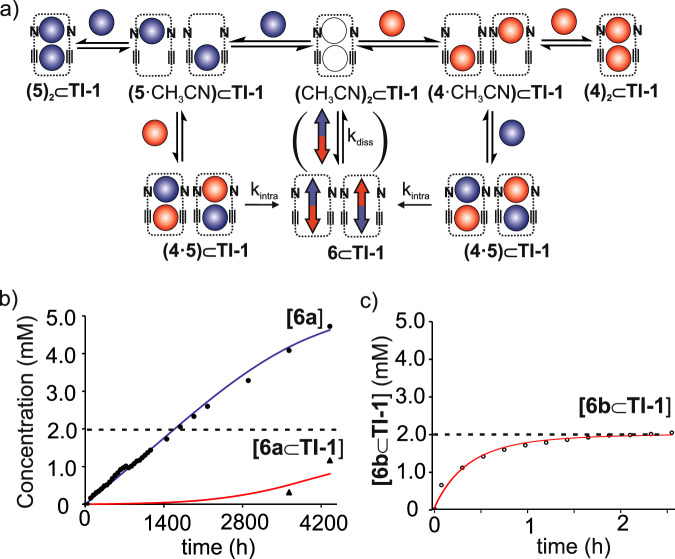
Kinetic analysis. **a** Global kinetic model; (**b**, **c**) ^1^H NMR-derived concentration profiles for reactions starting from **TI-1**:**4**:**5** = 1:4:4 [**TI-1**]_0_ = 2 mM in CDCl₃:CD₃CN (9:1): (**b**) **6a** (black circles) and **6a**⊂**TI-1** (black triangles); (**c**) **6b**⊂**TI-1** (empty black circles). Solid lines: fits from COPASI v4.25 with k_intra_ as the sole fitted parameter. Dashed lines indicate total **T1**-**1** cage concentration. See the Supplementary Dataset for experimental data of graphs (**b**) and (**c**).

As in our previous work^23–26^, we analyzed the complete time courses of the reactions with an elaborated kinetic model that includes all relevant supramolecular equilibria (substrate and product binding to **TI-1**) and a single irreversible intra-vessel cycloaddition step. The model comprises seven reversible binding equilibria and one irreversible step (ten species total) (Fig. 6a). Background cycloaddition in bulk is neglected because the estimated half-life at 2 mM is extremely long (see above), making its contribution negligible on the experimental time scale. Under strictly stoichiometric conditions (**TI-1**:**4**:**5** = 1:1:1), we further simplified the model by omitting product-dissociation equilibria.

Fitting the full 0–70 days’ time-course for the reaction of **4a** + **5** inside **TI-1** gives k_**6a**-intra_ = 2.0 ± 0.1 × 10^−6 ^s^−1^ (Supplementary Fig. S38 (2 mM) and S39 (8 mM)),  corresponding to EM_kin_ = k_**6a**-intra_/k_**6a**-bulk_ = 2.0 × 10^−6 ^s^−1^ / 5.6 × 10^−8^ M^−1^ s^−1^ = 36 ± 4 M. Assuming that the acceleration arises exclusively from entropic effects (i.e., *ΔΔH*^‡^ ≈ 0), this EM_kin_ corresponds to −7.1 e.u. (*ΔΔS*^*‡*^ = *ΔS*^‡^_bulk_ - *ΔS*^‡^_intra_ = - R ln(EM_kin_)) (see Supplementary Table S1 for details). This agrees with EM_kin_ ≈ 45 M derived from initial rates. Global fitting of the reaction **4b** + **5** inside **TI-1** yields k_**6b**-intra_ = 3.8 ± 0.3 × 10^−3 ^s^−1^ (Fig. 6c). This value corresponds to EM_kin_ = 1.1 ± 0.7 × 10^5^ M (*ΔΔS*^*‡*^ ≈ − 23 e.u.), approaching enzyme-like entropic contributions (i.e., ~ − 35 e.u.) (see Supplementary Table S1 for details)^13^. Notably, the **6b**⊂**TI-1** regioisomer AE:SAE ratio emerging from the intra-vessel reaction is ~ 1:1, whereas binding experiments starting from free **6b** give a kinetically trapped SAE:AE ~ 9:1 ratio. This suggests that the two Michaelis-complex orientations, (**4b**^AE^·**5**^SAE^)⊂**TI-1** and (**4b**^SAE^·**5**^AE^)⊂**TI-1**, are formed with similar probabilities and provide comparable acceleration/regioselectivity, while the resulting **6b**⊂**TI-1** isomers are kinetically locked and do not interconvert on the experimental time scale.

### Kinetic studies of the 1,3-dipolar cycloaddition reactions of 4a and 4b with 5 in the cavity of TI-1. Catalytic conditions

With substoichiometric **TI-1** (2 mM) and excess substrates **4a** and **5** (8 mM each), ^1^H NMR monitoring shows progressive accumulation of free **6a**, demonstrating turnover (Supplementary Fig. S40). Signals of **6a**⊂**TI-1** become prominent only after substantial conversion ( ~ 150 days, ~ 50%). Simultaneous fitting of the concentration changes of **6a** and **6a⊂TI-1** to the full kinetic model (including product dissociation, Fig. 6b) returns k_**6a**-intra_ = 2.6 × 10^−6 ^s^−1^ as the sole adjustable parameter, in agreement with stoichiometric experiments. After 180 days, the total product is ~ 6 mM ([**6a**] = 4.7 mM; [**6a**⊂**TI-1**] = 1 mM), corresponding to TON ≈ 3 at 75% conversion (Supplementary Fig. S41). Thus, **TI-1** combines modest acceleration (EM_kin_ ~ 30–40 M) with measurable turnover because **6a** binds weakly enough to be displaced by substrates under millimolar conditions. Turnover in vessel-mediated Huisgen cycloadditions is rare. In early cucurbituril systems^18,19^, product release could become rate‑limiting or inhibit turnover, making the present catalyst turnover under directional hydrogen‑bonding interactions particularly relevant.

Under analogous conditions with **4b** + **5** (8 mM each, **TI-1** 2 mM), the concentration of **6b**⊂**TI-1** rapidly reaches ~ 2 mM ( < 2 h) and remains unchanged over extended times, indicating complete product inhibition consistent with the inability of the substrates to displace **6b** from the cavity.

### DFT-optimized structures of 1,4-triazole 6 complexes with TI-1 and OI-1

We optimized the structures of the product inclusion complexes, **6a**^**AE**^⊂**TI-1** and **6b**^**AE**^⊂**TI-1**, using DFT calculations at the RI^30–32^-BP86^30^-def2-SV(P)^33,34^ level and performed single-point energy calculations at the RI^30–32^-BP86^30^-def-TZVP^33,34^ level with Turbomole 7.8^35^.

**Fig. 7:**
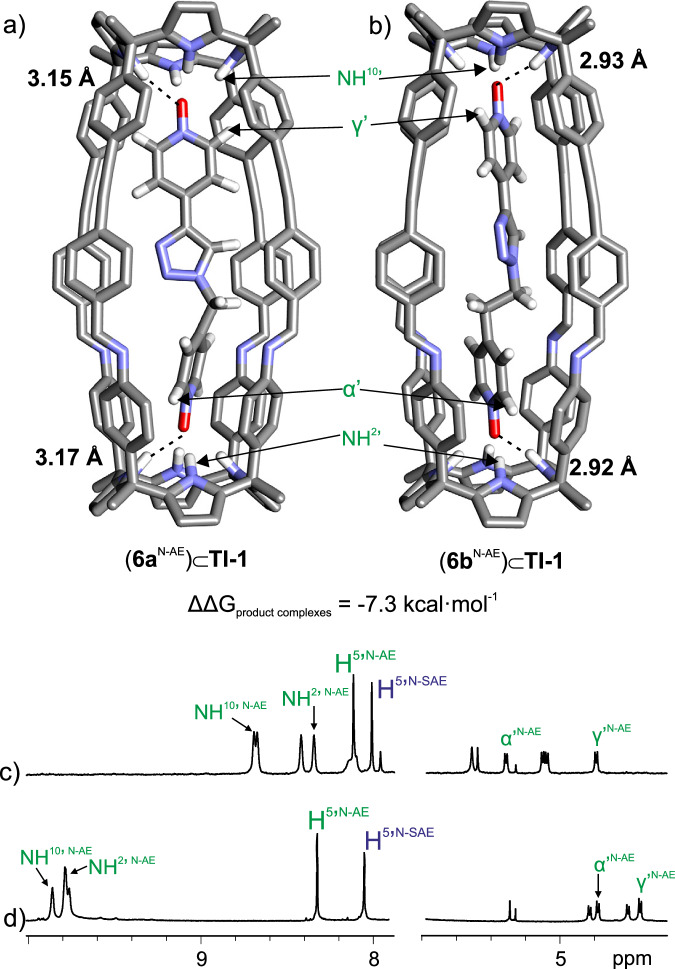
DFT structures and ^1^H NMR spectra of product inclusion complexes. Top) Energy-optimized structures (DFT) of the inclusion complexes **6a**^**AE**^⊂**TI-1** (**a**) and **6b**^**AE**^⊂**TI-1** (**b**). ∆ΔG_product-complexes_ = (G(**6b**^**AE**^⊂**TI-1**) – G(**5**^**SAE**^**•4b**^**AE**^)⊂**TI-1**)**-**(G(**6a**^**AE**^⊂**TI-1**) – G(**5**^**SAE**^**•4a**^**AE**^)⊂**TI-1**). ∆ΔG is obtained by referencing the energy of each product complex to its corresponding ternary complex. Bottom) Partial ^1^H NMR spectra (CDCl_3_:CD_3_CN (9:1)) of equimolar mixtures of: (**c**) **6a**^**AE**^⊂**TI-1**/**6a**^**SAE**^⊂**TI-1** and (**d**) **6b**^**AE**^⊂**TI-1**/**6b**^**SAE**^⊂**TI-1**.

Referencing each product complex to its corresponding ternary complex, the computed relative stabilization favors **6b**^**AE**^⊂**TI-1** by ∆∆G_product-complexes_ = – 7.3 kcal·mol⁻¹, in good agreement with the experimental trend in energy barriers (∆∆G^‡^ = − 4.5 kcal·mol⁻¹) between the reactions yielding **6b** *vs*. **6a**. Structurally, **6b**^**AE**^⊂**TI-1** exhibits shorter pyrrole HN⋯O(*N*-oxide), hydrogen bonds than **6a**^**AE**^⊂**TI-1** (average decrease Δd~0.23 Å) (Fig. 7a), approaching optimal distances seen for 1:1 AE-[C4]P pyridine-*N*-oxide complexes (2.9-3.0 Å)^28^. This indicates that the cavity of **TI**-**1** provides a better geometric match to both the confined reactive arrangement and the geometry of the corresponding TS for the reacting pair in (**4b** + **5**)⊂**TI-1** (see Supplementary Movies 1 and 2 for the animations of the imaginary frequency vibrational modes).

In contrast, the longer HN⋯O(*N*-oxide) distances in **6a**⊂**TI-1** imply a narrower span of the polar binding sites and a higher distortion penalty to reach the productive TS geometry from (**4a** + **5**)⊂**TI-1**, explaining lower EM_kin_ and weaker product binding required for turnover. The experimentally smaller NH CIS for **6a**⊂**TI-1** (CIS_NH_ = 1 ppm) *vs*. **6b**⊂**TI-1** (CIS_NH_ = 2.2-2.5 ppm) is consistent with these calculated hydrogen-bond metrics (Fig. 7c, d).

The analogous HN⋯O(*N*-oxide) distances in **6a**⊂**OI-1** and **6b**⊂**OI-1** are both optimal ( ~ 2.93 Å), supporting strong TSs complementarity, and consequently, large accelerations accompanied by product inhibition.

### Comparison of Huisgen cycloadditions kinetics in OI-1 and TI-1 cavities

**TI-1** differs from **OI-1**^23^ by replacing four imine bonds (C-N = C-C, 3.7 Å) with alkynes (C-C ≡ C-C, 4.3 Å), producing a sub-Ångström cavity elongation ( ~ 0.6 Å) while retaining the polar binding sites. This small geometric change substantially reshapes the balance between acceleration and turnover (Table 2).

**Table 2:** Rate constants (k_intra_) for the 1,3-dipolar cycloadditions of 5 with 4a and 4b within TI-1 and OI-1 cavities

Cage	(4a + 5) k_6a-intra_	(4b + 5) k_6b-intra_
**TI-1**	2.0 ± 0.1 × 10^−6 ^s^−1^	3.8 ± 0.3 × 10^−3 ^s^−1^
**OI-1**	5.0 ± 1.0 × 10^−5 ^s^−1^	8.1 ± 1.0 × 10^−5 ^s^–1^

For **4a** + **5,** **TI-1** maintains high 1,4-regioselectivity yet gives only moderate acceleration (EM_kin_ ~ 30 M); importantly, product **6a** binds weakly enough to allow **TI-1** turnover (TON ~ 3 at 75% conversion over 180 days under millimolar conditions). In contrast, **OI-1** accelerates the same reaction much more strongly (EM_kin_ ~ 10^3 ^M) but suffers from product inhibition.

This trend is reversed for the reacting pair involving the longer azide **4b** and **5** (Table 2). The **TI-1** provides an enzyme-like entropic enhancement (EM_kin_~ 10^5 ^M), whereas the **OI-1** cage shows a significantly smaller effect (EM_kin _= 10^3 ^M). However, both cages are subjected to product inhibition for **6b** due to tight product binding, preventing turnover.

**Fig. 8:**
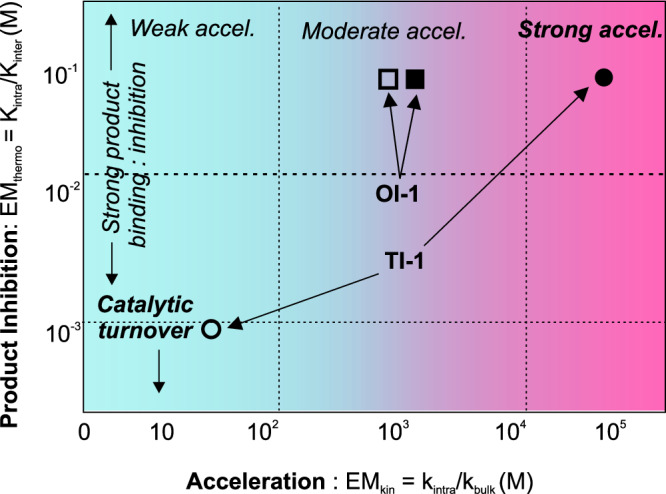
Acceleration–turnover trade-off in molecular cages. Acceleration–turnover trade-off for **OI-1** (squares) and **TI-1** (circles) molecular cages. EM_kin_ (*x*) *vs*. EM_thermo_ (*y*) for reactions of azides **4a** + **5** (open symbols) and **4b** + **5** (filled symbols). Sub-Å cavity elongation shifts the balance between rate enhancement and product inhibition toward improved turnover for **4a** + **5** in **TI-1**.

Overall, sub-Ångström elongation of the cavity in **TI-1** compared to **OI-1** (squares in Fig. 8) decouples entropic acceleration, achieved through effective substrate co-binding and TS preorganization, from deleterious product binding (**4a** + **5** open circle, and **4b** + **5** closed circle in Fig. 8). Notably, 1,4-regioselectivity is conserved in both reactor vessels, indicating that the kinetics are dominated by the geometry of the productive Michaelis complex.

To conclude, we report the quantitative self-assembly of the [1 + 1] tetraimine bis-calix[4]pyrrole cage **TI-1** and establish its function as a molecular vessel for Huisgen 1,3-dipolar cycloadditions of *para*-substituted pyridine-*N*-oxides. **TI-1** forms thermodynamically stable 1:1 and 2:1 inclusion complexes with azides **4a**/**4b** and alkyne **5**; binding constants were obtained from ¹H NMR and ITC for monotopic guests. Competitive binding analysis yielded significant differences in product binding (EM_thermo_ ≈ 10⁻³ M for **6a** and EM_thermo_ > 0.1 M for **6b**), predicting turnover only for the shorter-linker product **6a**.

Inside **TI-1**, both substrate pairs react with high 1,4-regioselectivity. For **4a** + **5**, **TI-1** delivers moderate acceleration (EM_kin _= 36 M), yet enables slow but measurable turnover under millimolar conditions (TON ≈ 3, 75% conversion, 180 days) because **6a** binds weakly enough to be displaced by substrates. Extending the azide linker (**4b**) yields near enzyme-like entropic acceleration (EM_kin _= 1.1 × 10^5 ^M) but causes product inhibition, as **6b** cannot be displaced by starting materials.

Comparison with the octa-imine analog **OI-1** shows that the sub-Å cavity elongation ( ~ 0.6 Å) in **TI-1** rebalances acceleration *vs*. turnover without compromising 1,4-regioselectivity. DFT-optimized product complexes support a TS-complementarity picture in which the better geometric match of **6b**⊂**TI-1** strengthens hydrogen-bonding contacts and product binding, rationalizing both the larger acceleration and the inhibition observed in the **4b** + **5** pathway.

Overall, our results address a recurring limitation encountered in many examples of confined catalysis: product inhibition. We demonstrate that sub-Å cavity tuning of reactor vessels modulate the acceleration–inhibition trade–off in supramolecular systems that rely on entropic effects and involve directional interactions to bind substrates and products.

## Methods

*NMR Spectroscopy* - Routine ^1^H NMR and ^13^C NMR spectra were recorded on a Bruker Avance 400 (400 MHz for ^1^H NMR and 100 MHz for ^13^C NMR), Bruker Avance 500 (500 MHz for ^1^H NMR and 125 MHz for ^13^C NMR). Otherwise stated, NMR experiments were performed at 298 K. Chemical shifts are reported in ppm relative to the residual ^1^H signal of the deuterated solvent used. COSY, NOESY, and ROESY experiments were recorded to aid proton assignment.

*Mass Spectrometry* - Mass spectrometry experiments were performed on a BRUKER Autoflex matrix-assisted laser desorption ionization (MALDI) time-of-flight mass spectrometer.

*Isothermal titration calorimetry (ITC)* - ITC experiments were performed using a MicroCal VP-ITC MicroCalorimeter with VP Viewer 2000 software (MicroCal, version 7.0). All the titrations were carried out in a chloroform: acetonitrile 9:1 solution mixture at 298 K. Titrations for monotopic guests were carried out by adding small aliquots (8 μL, 16 s) of a solution of the guest into a solution of the host in the same solvent mixture. The injection spacing was set based on the different kinetics of guest inclusion (from 900 s (guest **5**) to 1800 s (guests **4a** and **4b**). The concentration of the guest solution was approximately sixteen times that of the host solution. The association constants and the thermodynamic parameters were obtained from the fit of the titration data to the “one set of sites” binding model implemented in the Microcal ITC Data Analysis software.

*Single crystal X-ray diffraction -* X-ray diffraction experiments were performed using a Rigaku MicroMax-007HF diffractometer equipped with a PILATUS 200 K detector and a Bruker Apex II Duo with an APEX II detector, both using Mo Kα radiation. Structures were solved using VLD and Patterson methods implemented in SIR2014 v14.10 and refined by the least-squares method on F2 with SHELXL-2018/3. Due to the limited resolution of the diffraction data and the resulting low data-to-parameter ratio, additional restraints and constraints were applied to ensure a stable refinement and chemically reasonable structural model. Specifically, a global RIGU restraint was employed, supplemented by atom-specific RIGU and ISOR restraints where necessary. EADP constraints were applied to selected atom pairs in close proximity or disordered environments to further stabilize their displacement parameters.

*Kinetic studies -* To determine the rate constants of the reactions studied, the kinetic data were mathematically analyzed using COPASI 4.43 software, employing an elaborated theoretical kinetic model.

## Supplementary information

Supplementary Information

Description of Additional Supplementary Files

Supplementary Movie 1

Supplementary Movie 2

Transparent Peer Review file

## Data Availability

All data generated or analyzed during this study are included in this published article (and its supplementary information files). The datasets generated during the current study are available in the figshare repository, 10.6084/m9.figshare.31819954. Moreover, the dataset containing the computational results for this manuscript is available in the ioChem-BD repository^36^ and can be accessed via 10.19061/iochem-bd-1-418. The X-ray crystallographic coordinates for the structure reported in this study have been deposited at the Cambridge Crystallographic Data Center (CCDC), under deposition number 2528452. All data are available from the corresponding author upon request.
