# Supplementary material for: Balancing acceleration and turnover in [1 + 1] tetra-imine bis-calix[4]pyrrole reactor for Huisgen cycloadditions
Source: Nat Commun. 2026 Apr 27;17:6525. doi: 10.1038/s41467-026-72315-w (PMC13377019; doi:10.1038/s41467-026-72315-w)
Supplement: Supplementary file 1 — Supplementary Information [file 41467_2026_72315_MOESM1_ESM.pdf]

Balancing acceleration and turnover in [1 + 1] tetra-imine bis-calix[4]pyrrole reactor for Huisgen cycloadditions.

Yifan Li,[a] Gemma Aragay,[a] Pablo Ballester*[a],[b]

Supplementary Information

1. Materials and General Methods S2
2. Synthesis and characterization data S3
3. Binding studies of tetra-imine cage **TI-1** with monotopic para-substituted pyridine-*N*-oxide substrates **4a**, **4b**, and **5**. S11
4. Binding studies of tetra-imine cage **TI-1** with ditopic bis-pyridine-*N*-oxide cycloaddition products **6a** and **6b**. S20
5. Kinetic characterization of cycloaddition reactions inside **TI-1** S32
   5.1. Cycloaddition reaction of **4a** with **5** S32
   5.2. Cycloaddition reaction of **4b** with **5** S41
6. DFT calculations S45
7. X-ray crystal structure S47
8. References S48

1. Materials and General Methods

All reagents and solvents were purchased from commercial suppliers and used without further purification. All reactions were performed under Ar atmosphere unless otherwise specified. All solvents were of HPLC grade quality, commercially obtained, and used without further purification except pyrrole, which was distilled and freshly used. Anhydrous solvents were obtained from the SPS-400-6 solvent purification system (Innovative Technologies).

Routine ^{1}H NMR and $^{13}C\{^{1}H\}$NMR spectra were recorded on a Bruker Avance 400 (400 MHz for ^{1}H NMR and 100 MHz for ^{13}C NMR), Bruker Avance 500 (500 MHz for ^{1}H NMR and 125 MHz for ^{13}C NMR). Otherwise stated, NMR experiments were performed at 298 K. Chemical shifts are reported in ppm relative to the residual ^{1}H signal of the deuterated solvent used. ^{1}H NMR splitting patterns were designated as singlet (s), doublet (d), triplet (t), quartet (q), or multiplet (m). Coupling constants (J) were given in Hz. COSY, NOESY, and ROESY experiments were recorded to aid proton assignment.

Mass spectrometry experiments were performed on a BRUKER Autoflex matrix-assisted laser desorption ionization (MALDI) time-of-flight mass spectrometer.

Isothermal titration calorimetry (ITC) experiments were performed using a MicroCal VP-ITC MicroCalorimeter with VP Viewer 2000 software (MicroCal, version 7.0). All the titrations were carried out in a chloroform: acetonitrile 9:1 solution mixture at 288 K. Titrations for monotopic guests were carried out by adding small aliquots (8 µL, 16 s) of a solution of the guest into a solution of the host in the same solvent mixture. The injection spacing was set based on the different kinetics of guest inclusion (from 900 s (guest **5**) to 1800 s (guests **4a** and **4b**). The concentration of the guest solution was approximately sixteen times that of the host solution. The association constants and the thermodynamic parameters were obtained from the fit of the titration data to the "one set of sites" binding model implemented in the Microcal ITC Data Analysis software.

Single crystal X-ray diffraction experiments were performed using a Rigaku MicroMax-007HF diffractometer equipped with a PILATUS 200K detector and a Bruker Apex II Duo with an APEX II detector, both using Mo Kα radiation. Structures were solved using VLD and Patterson methods implemented in SIR2014 v14.10 and refined by the least-squares method on F2 with SHELXL-2018/3. Due to the limited resolution of the diffraction data and the resulting low data-to-parameter ratio, additional restraints and constraints were applied to ensure a stable refinement and chemically reasonable structural model. Specifically, a global RIGU restraint was employed, supplemented by atom-specific RIGU and ISOR restraints where necessary. EADP constraints were applied to selected atom pairs in close proximity or disordered environments to further stabilize their displacement parameters.

To determine the rate constants of the reactions studied, the kinetic data were mathematically analyzed using COPASI 4.43 software, employing an elaborated theoretical kinetic model.

2. Synthesis and characterization data

-Tetra-formyl tetra-chloro super aryl-extended calix[4]pyrrole SAE-C[4]P ***2****.*

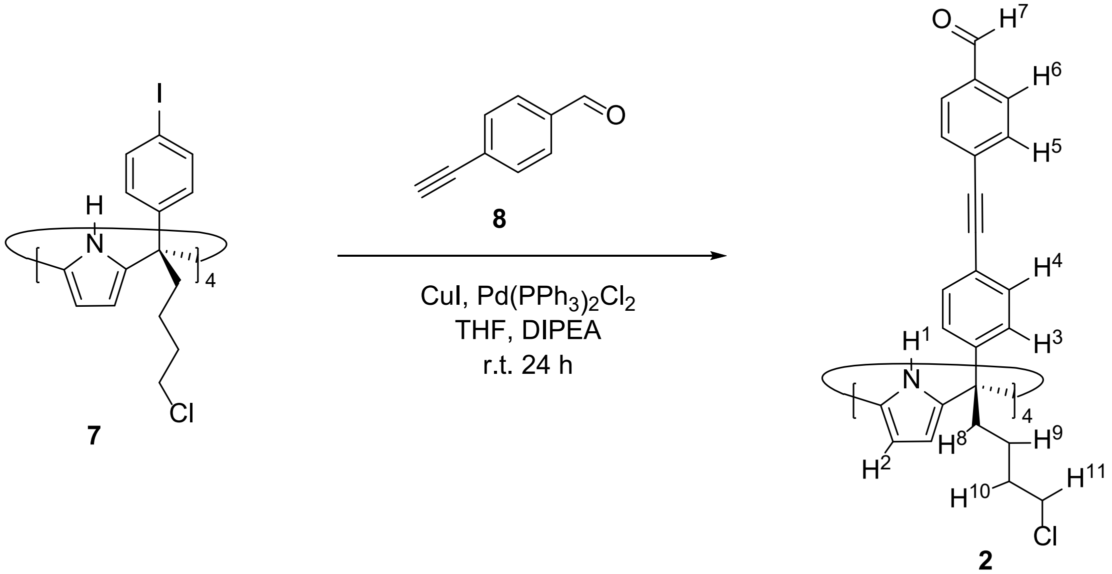

Figure S 1. Synthetic scheme for the preparation of tetra-formyl tetra-chloro super-aryl extended C[4]P **2**.

In a 50 mL Schlenk flask, tetra-α 4-iodophenyl-4'-chlorobutyl C[4]P **7** (320 mg, 215 μmol, 1 equiv), $Pd(PPh_3)_2Cl_2$ (7.55 mg, 10.7 μmol, 0.05 equiv), CuI (4.10 mg, 21.5 μmol, 0.1 equiv), and 4-ethynyl benzaldehyde **8** (224 mg, 1.72 mmol, 8 equiv) were mixed and kept under argon atmosphere. Dry THF (15.0 mL) and dry diisopropylamine (15.0 mL) were added. The mixture was stirred at room temperature for 24 hours. Afterward, the solvent was removed under reduced pressure, and the crude product was redissolved in DCM (100 mL), then washed with brine (2 × 50 mL) and water (50 mL). The organic layer was dried over Na_2SO_4, filtered, and concentrated. The crude product was purified by column chromatography on silica gel (10 g, 100:0 → 97:3 DCM: EtOAc) to yield a yellow solid. This yellow solid was further purified by recrystallization from a 1:1 mixture of DCM and CH_3CN, resulting in the final product (205 mg, 137 μmol, 60% yield).

SAE-C[4]P **2** (205 mg, 60%): Rf = 0.4 (DCM, 3% EtOAc). ^{1}H NMR (500 MHz, 298 K, DMF-*d*$_7$): δ = 10.05 (s, 4H), 8.88 (s, 4H), 7.79 (d, J = 8.2 Hz, 8H), 7.75 (d, J = 8.1 Hz, 8H), 7.60 (d, J = 8.2 Hz, 8H), 7.10 (d, J = 8.1 Hz, 8H), 6.13 (d, J = 2.6 Hz, 8H), 3.66 (t, J = 6.6 Hz, 8H), 2.46 (t, J = 8.4 Hz, 8H), 1.79 (tt, J = 6.9 Hz, 6.6 Hz, 8H), 1.31 (tt, J = 6.9 Hz, 8.4 Hz, 8H). ^{13}C NMR (124 MHz, 298K, 9:1 $CDCl_3$:CD_3CN). The solubility of SAE-C[4]P **2** in DMF was not sufficient to acquire a reliable ^{13}C NMR spectrum. HR-MS (MALDI+) m/z: $[M]^+$calculated for $C_{96}H_{80}Cl_4N_4O_4$ 1492.4928, found 1492.4940.

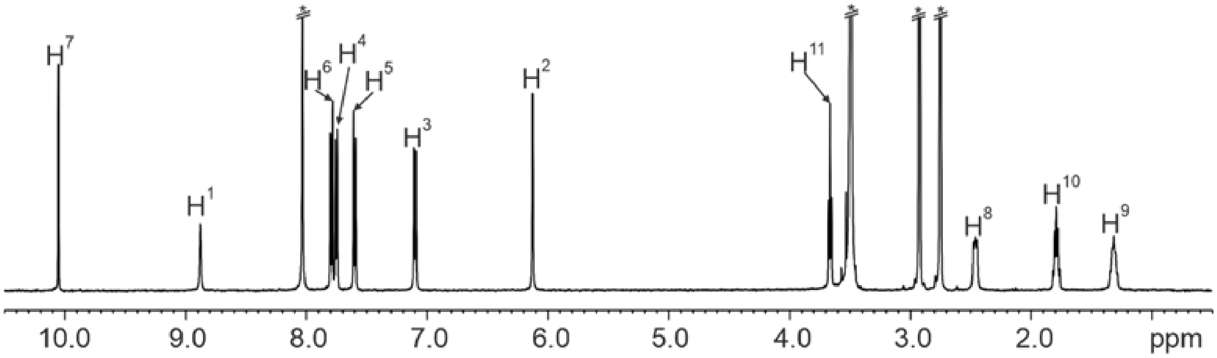

Figure S 2. ^{1}H NMR spectrum (500 MHz, at 298 K, DMF-d_7) of SAE-C[4]P **2**.

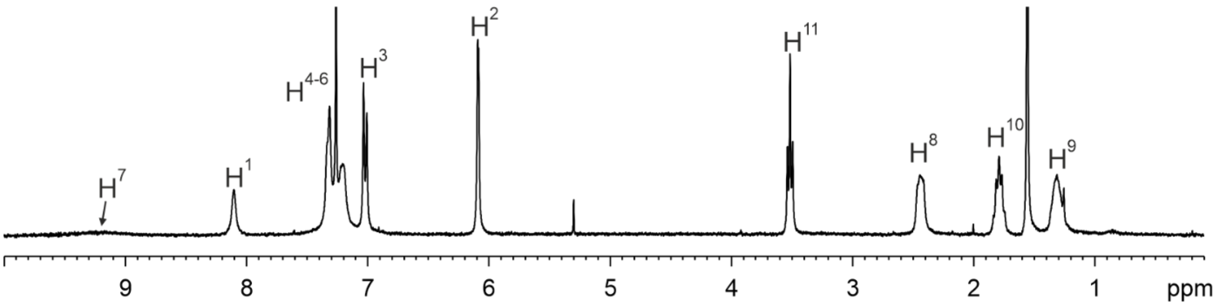

Figure S 3. ^{1}H NMR spectrum (500 MHz, at 298 K, $CDCl_3$) of SAE-C[4]P **2**.

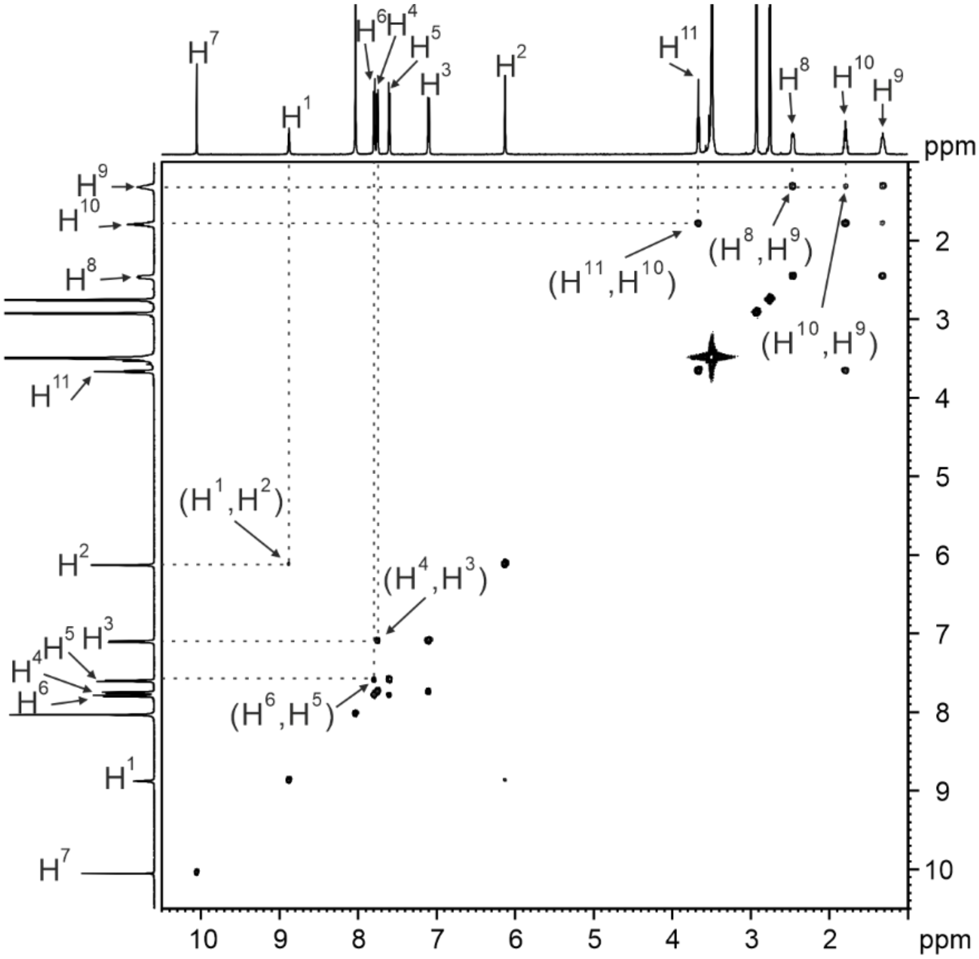

Figure S 4. Selected region of ^{1}H-^{1}H COSY NMR (500 MHz, at 298 K, DMF-d_7) of SAE-C[4]P **2**.

*-Tetra-imine cage **TI-1**.*

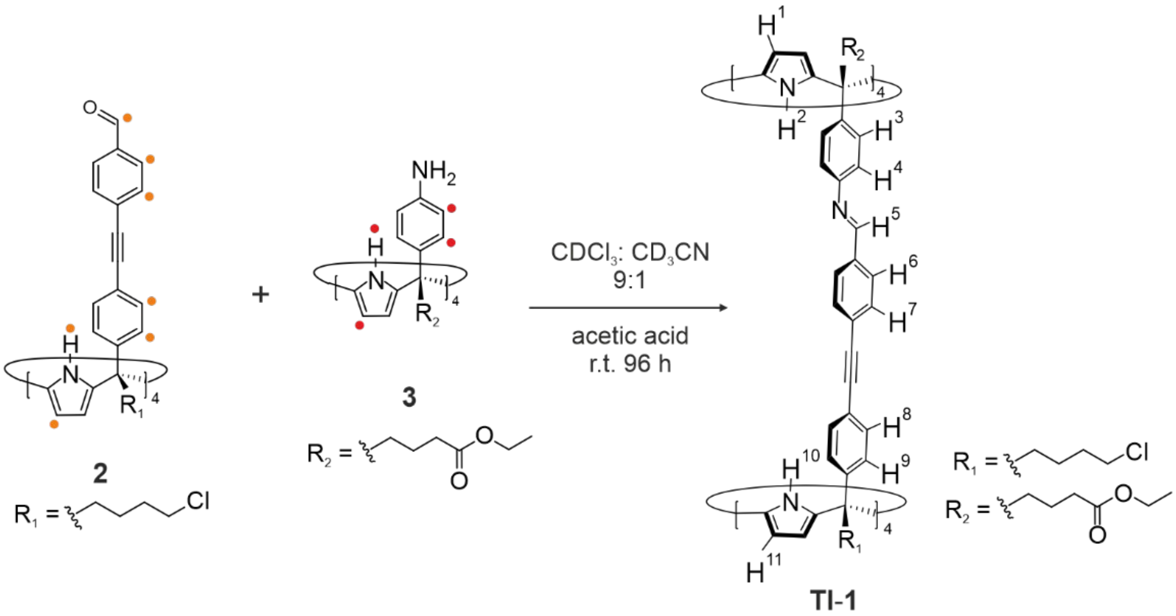

*Figure S 5. Synthetic scheme for the preparation of cage **TI-1**.*

NMR-scale synthesis: Tetra-formyl SAE-C[4]P **2** (1.50 mg, 1.00 µmol, 1 equiv) and tetra-amine AE-C[4]P **3** (1.10 mg, 1.00 µmol, 1 equiv) were placed in a J-Young NMR tube with 500 µL of a 9:1 $CDCl_3$:CD_3CN solvent mixture containing 0.01 mM acetic acid. 1,3,5-trimethoxybenzene was added as internal standard (i.s. final concentration = 1 mM). The solution was left at room temperature for 96 hours. The mixture turned yellow, and the ^1H NMR confirmed the formation of cage **TI-1** in a 90% yield (based on the relative integrals of the cage signals and those of the i.s. in solution).

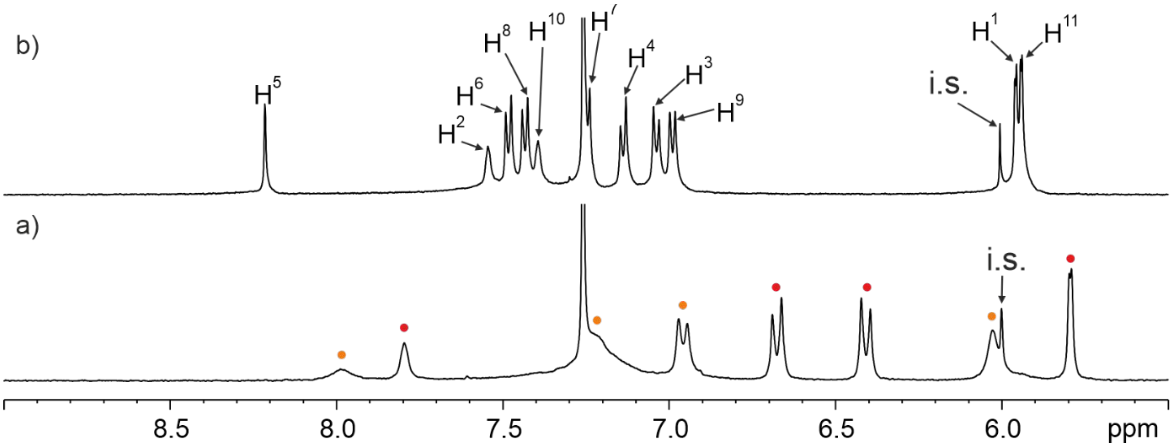

Figure S 6. Selected downfield region of the ^1H NMR spectra (500 MHz, at 298 K, $CDCl_3$:CD_3CN 9:1 mixture) of (a) 1:1 mixture of SAE-C[4]P **2** and AE-C[4]P **3** immediately after mixing the two components and (b) after 96 h at 298 K. The proton signals in panel (b) are diagnostic of the quantitative self-assembly of the tetra-imine cage **TI-1**. See *Figure S 5* for the proton assignment.

mg scale synthesis of **TI-1:** In a 25 mL previously oven-dried Schlenk flask, tetra-formyl SAE-C[4]P **2** (29.8 mg, 20.0 μmol, 1 equiv) and tetra-amine AE-C[4]P **3** (22.8 mg, 20.0 μmol, 1 equiv.) were dissolved in 10 mL of a $CHCl_3$:CH_3CN 9:1 mixture containing 0.01 mM of acetic acid. The mixture was stirred at room temperature under argon, protected from light for 120 hours. Subsequently, the solvent was removed under vacuum. The resulting crude solid was redissolved in 5 mL of DCM and 0.5 mL of methanol was added. The solution was concentrated under reduced pressure, yielding cage **TI-1**, which precipitated as a yellow solid. The precipitate was filtered and washed with methanol to give pure cage **TI-1** (40.0 mg, 16.0 μmol, 80% yield) as a yellow solid.

Cage **TI-1** (40 mg, 80%). ^{1}H NMR (500 MHz, 298 K, 9:1 $CDCl_3$:CD_3CN) δ = 8.21 (s, 4H), 7.54 (bs, 4H), 7.48 (d, *J* = 7.9 Hz, 8H), 7.43 (d, *J* = 7.9 Hz, 8H), 7.39 (bs, 4H), 7.25 (d, *J* = 8.1 Hz, 8H), 7.14 (d, *J* = 8.1 Hz, 8H), 7.03 (d, *J* = 8.1 Hz, 8H), 6.98 (d, *J* = 8.1 Hz, 8H), 5.95 (d, *J* = 2.7 Hz, 8H), 5.94 (d, *J* = 2.7 Hz, 8H), 4.05 (q, *J* = 7.1 Hz, 8H), 3.44 (t, *J* = 6.6 Hz, 8H), 2.34 (m, 16H), 2.21 (t, *J* = 7.3 Hz, 8H), 1.70 (t, *J* = 7.3 Hz, 8H), 1.54 – 1.44 (m, 8H), 1.34 – 1.26 (m, 8H), 1.18 (t, *J* = 7.1 Hz, 12H). ^{13}C NMR (124 MHz, 298K, 9:1 $CDCl_3$:CD_3CN) δ = 173.4, 159.9, 158.5, 158.0, 148.6, 147.7, 146.8, 144.5, 137.1, 136.9, 135.6, 131.6, 131.0, 130.9, 130.6, 130.0, 128.6, 125.7, 120.4, 120.0, 105.0, 104.8, 90.8, 89.0, 60.2, 48.3, 48.0, 44.8, 38.6, 38.3, 34.4, 32.9, 22.2, 20.5, 14.2, 14.1 ppm. HR-MS (MALDI+) m/z: $[M]^+$ calculated for $C_{164}H_{152}Cl_4N_{12}O_8$ 2557.0605, found 2557.0578.

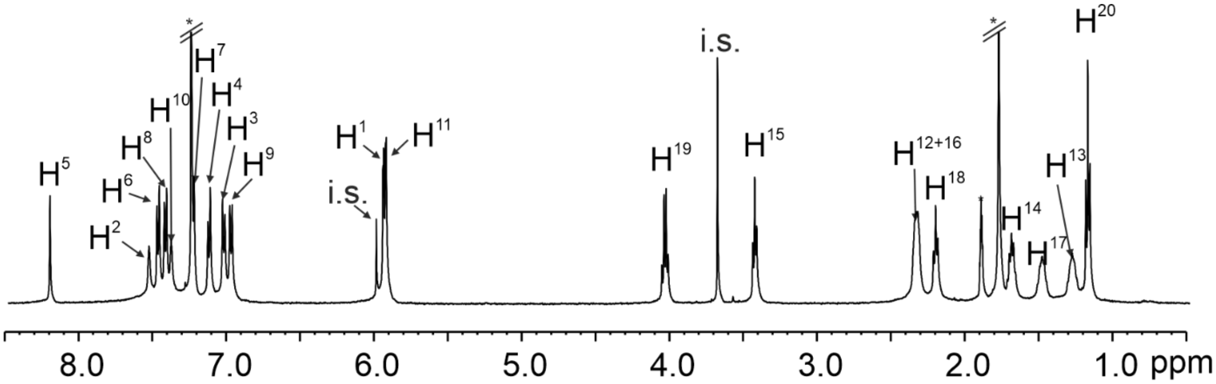

Figure S 7. ^{1}H NMR spectrum (500 MHz, at 298 K, $CDCl_3$:CD_3CN 9:1) of **TI-1**.

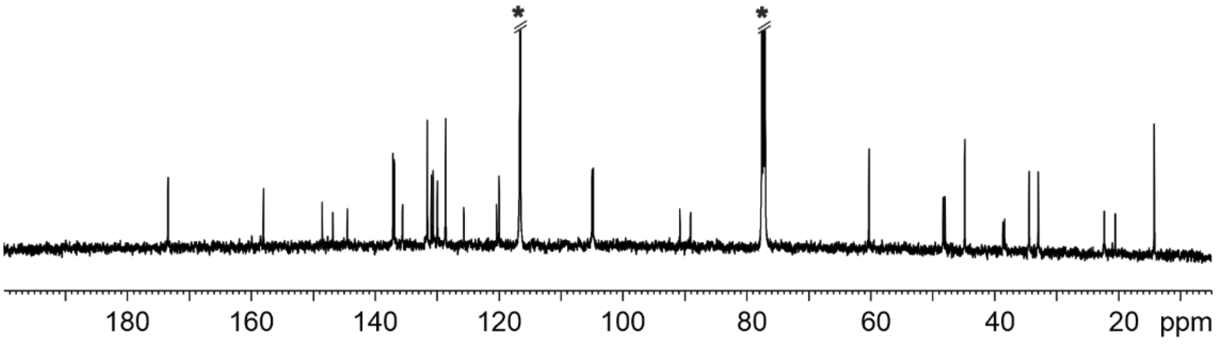

Figure S 8. ^{13}C NMR spectrum (124 MHz, at 298 K, $CDCl_3$: CD_3CN 9:1) of **TI-1**.

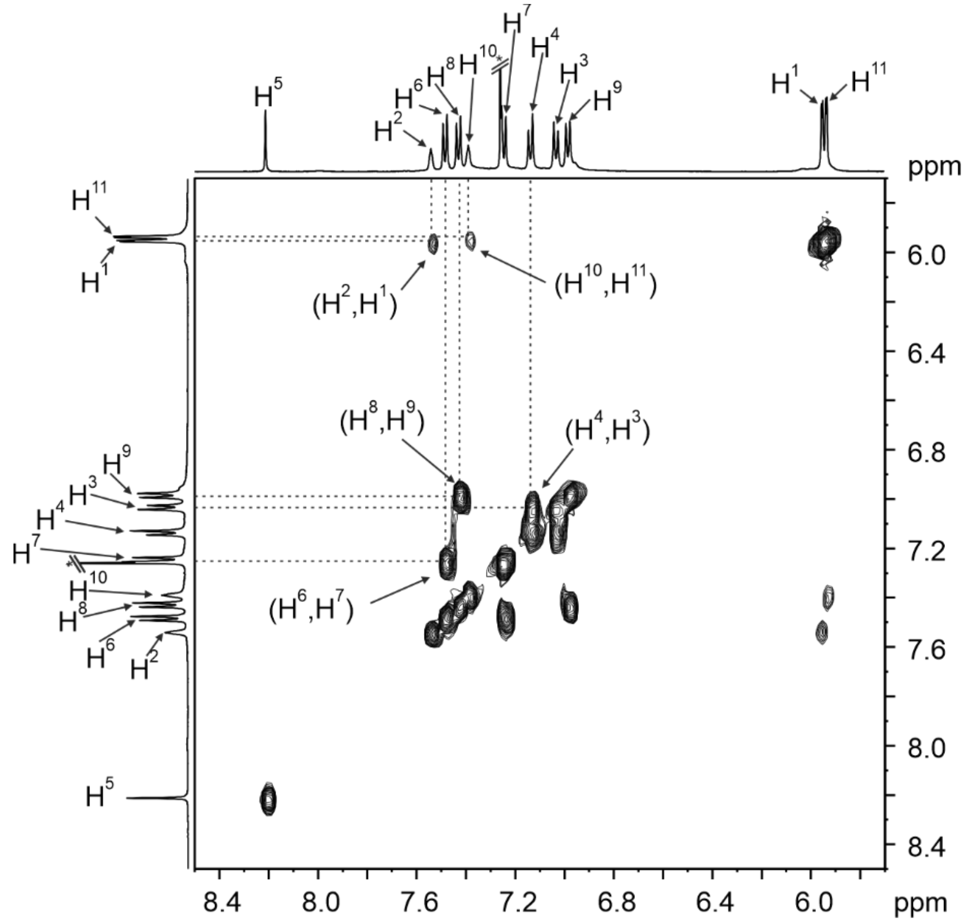

Figure S 9. Selected downfield region of ^1H-^1H COSY NMR (500 MHz, at 298 K, $CDCl_3$:CD_3CN 9:1) of a 2 mM solution of **TI-1**.

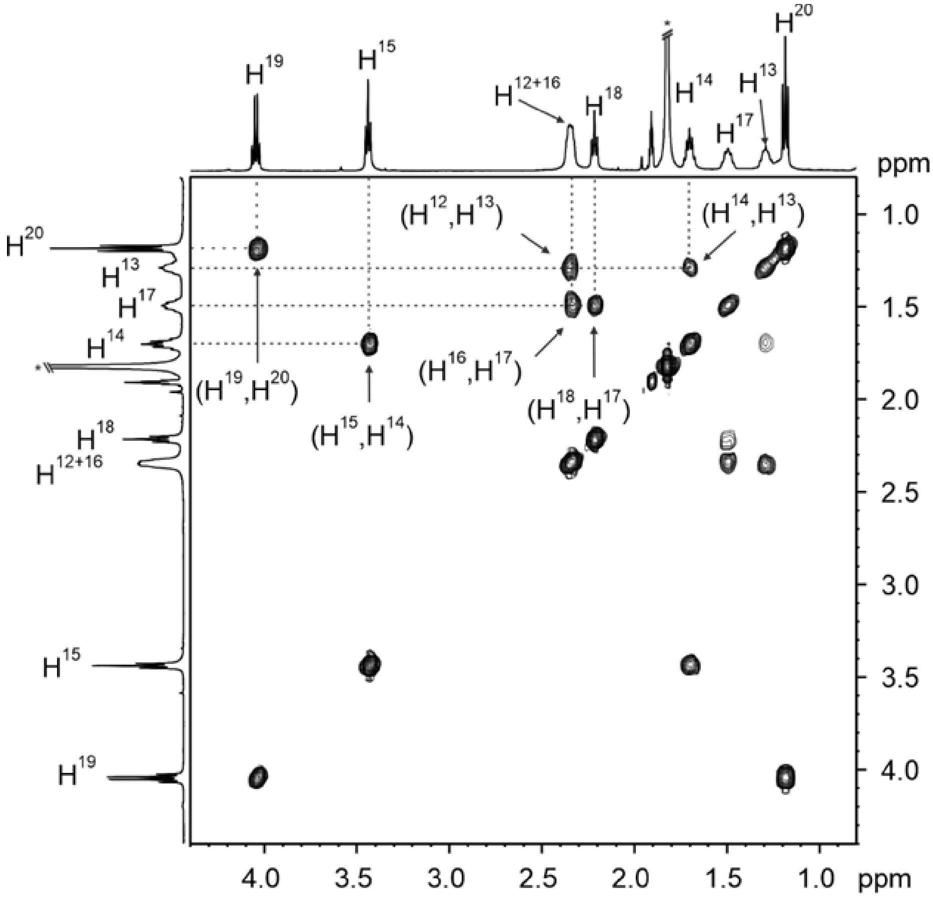

Figure S 10. Selected upfield region of ^{1}H-^{1}H COSY NMR (500 MHz, at 298 K, $CDCl_3$: CD_3CN 9:1) of a 2 mM solution of **TI-1**.

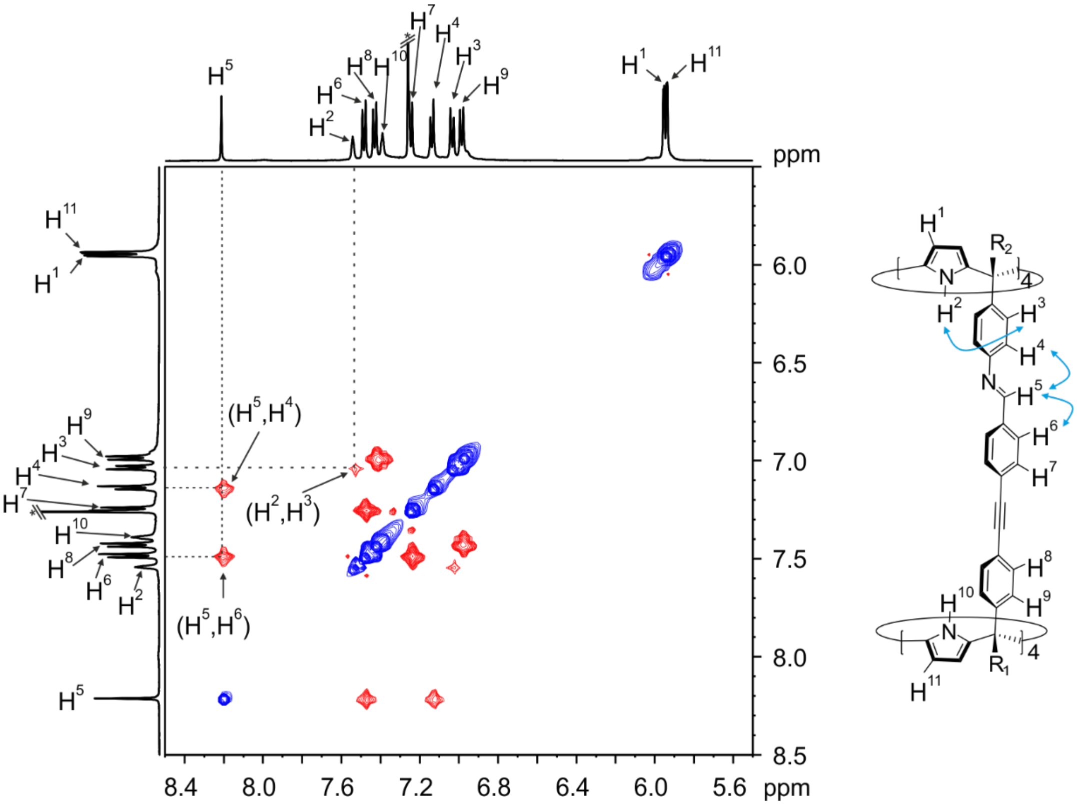

Figure S 11. Selected downfield region of 2D ^{1}H ROESY NMR (500 MHz, at 298 K, $CDCl_3$:CD_3CN 9:1, d8 (mixing time) = 300 ms) of **TI-1**.

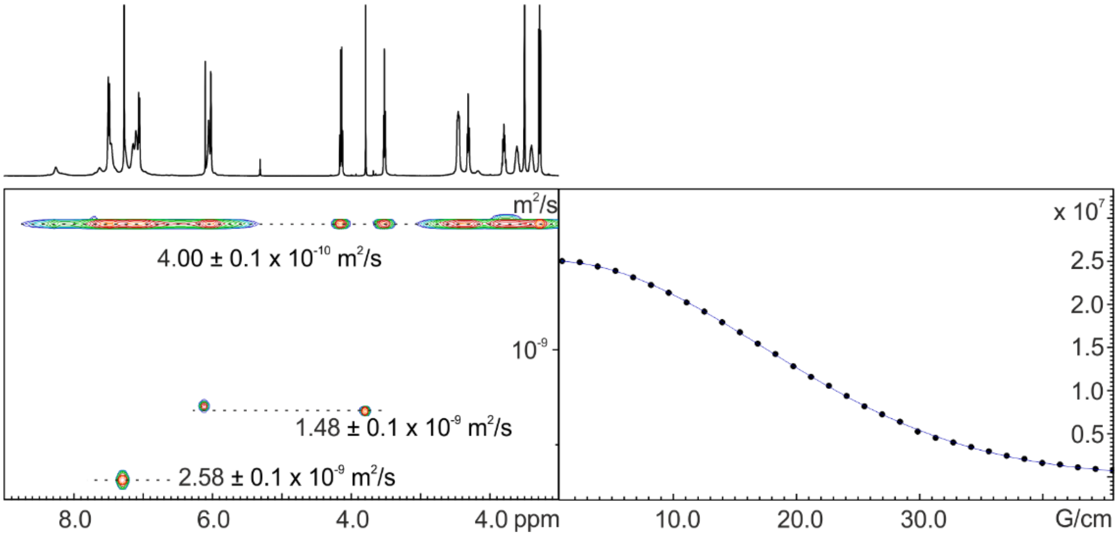

Figure S 12. (left) ^{1}H pseudo 2D plot of the DOSY experiment (500 MHz, 298 K, $CDCl_3$, D20 = 150 ms; P30 = 1 ms) of **TI-1**. (right) Fit of the decay of the proton signal resonating at δ= 6.1 ppm corresponding to β-pyrrole protons to a mono-exponential Stejskal–Tanner function. Errors are indicated as the standard deviation.

3. Binding studies of tetra-imine cage TI-1 with monotopic para-substituted pyridine-*N*-oxide substrates 4a, 4b, and 5.

3.1. ^{1}H NMR spectroscopic titrations

- *Binding studies of **TI-1** with 4-ethynyl pyridine-N-oxide **5***

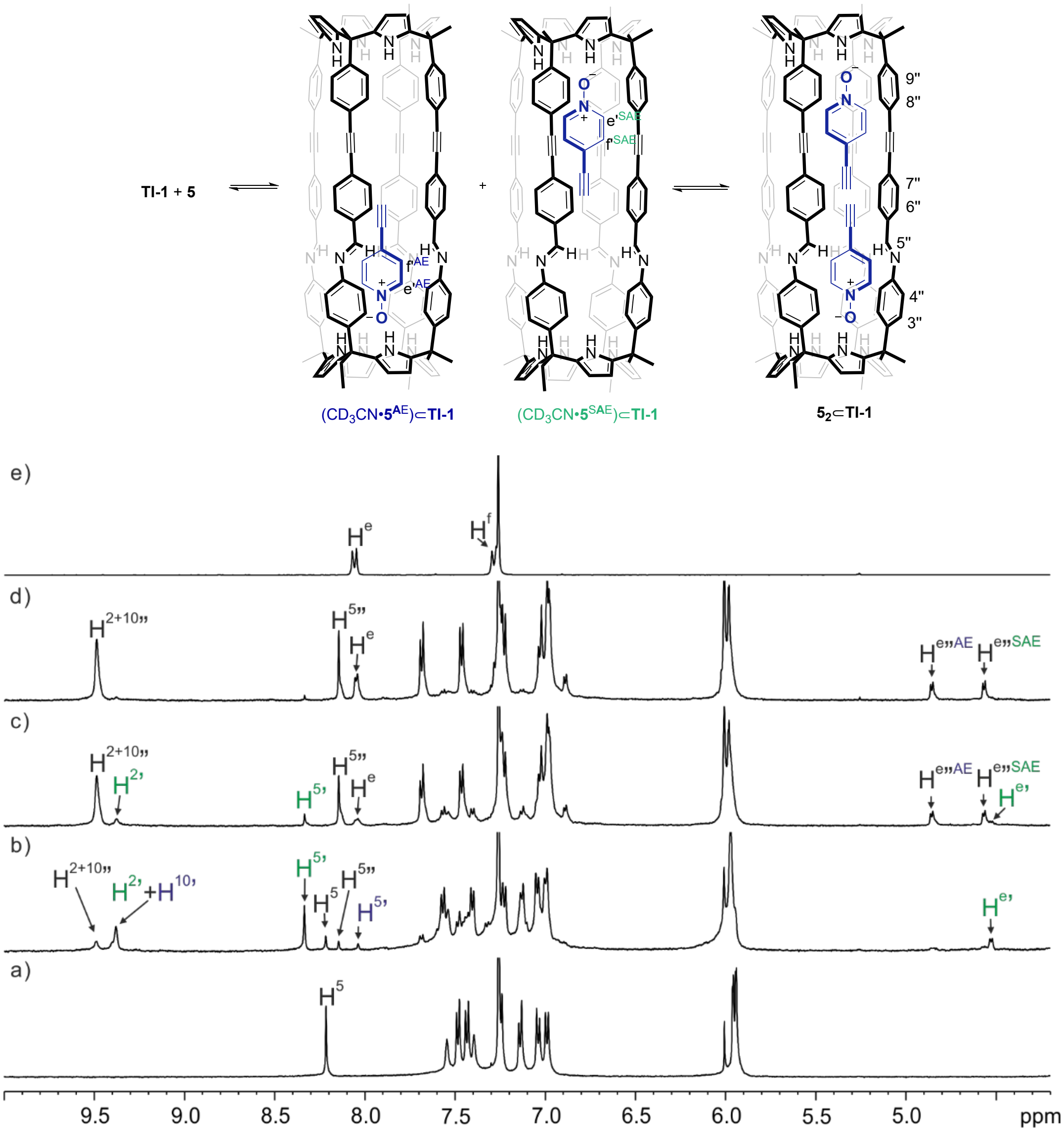

Figure S 13. Top) Binding equilibria of **TI-1** with guest **5** to produce two regioisomeric 1:1 complexes, and one 2:1 complex. Bottom) Selected region of the ^{1}H NMR (500 MHz, at 298K, $CDCl_3$:CD_3CN 9:1) spectra acquired during the titration of a 2 mM solution of **TI-1** with incremental additions of 4-ethynyl pyridine-*N*-oxide **5**: a) 0 equiv, b) 1 equiv, c) 2 equiv, d) 3 equiv. Spectrum e) corresponds to the free **5** in the same solvent mixture. Primed and double-primed numbers correspond to 1:1 and 2:1 complexes, respectively. Blue and green colors indicate the guest protons at the two possible hemispheres: aryl-extended AE (blue), and super aryl-extended SAE (green).

- *Binding studies of **TI-1** with 4-azido(methyl) pyridine-N-oxide **4a***

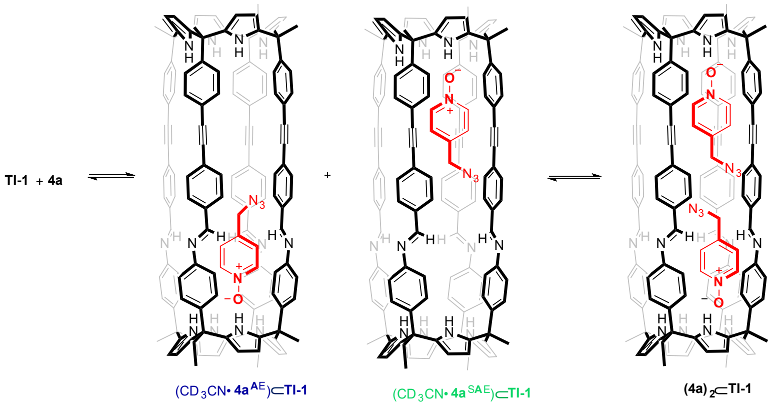

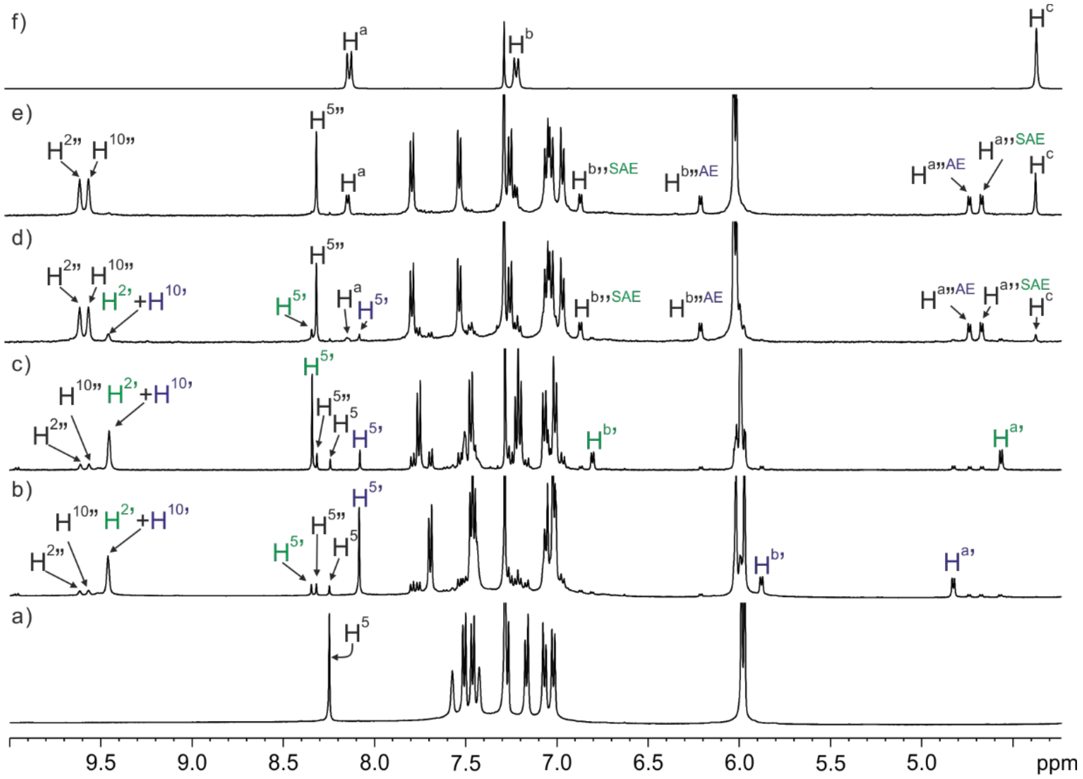

Figure S 14. Top) Binding equilibria of **TI-1** with guest **4a** to produce two regioisomeric 1:1 complexes, and one 2:1 complex. Bottom) Selected region of the ^1H NMR (500 MHz, at 298K, $CDCl_3$:CD_3CN 9:1) spectra acquired during the titration of a 2 mM solution of **TI-1** with incremental additions of **4a**: a) 0 equiv, b) immediately after the addition of 1 equiv of **4a**, c) 1 equiv of **4a** after 12 hours, d) 2 equiv, e) 3 equiv. Spectrum f) corresponds to the free **4a** in the same solvent mixture. Primed and double-primed numbers correspond to 1:1 and 2:1 complexes, respectively. Blue and green colors indicate the guest protons at the two possible hemispheres: aryl-extended AE (blue), and super aryl-extended SAE (green). $k_{off} \leq 0.002\ s^{-1}$ *was determined using COPASI's parameter estimation function and* ^1H *NMR exchange kinetic data of* *(CD_3CN•**4a**^{AE})⊂**TI-1** and (CD_3CN•**4a**^{SAE})⊂**TI-1**.*

- *Binding studies of **TI-1** with 4-azido(methyl) pyridine-N-oxide **4b***

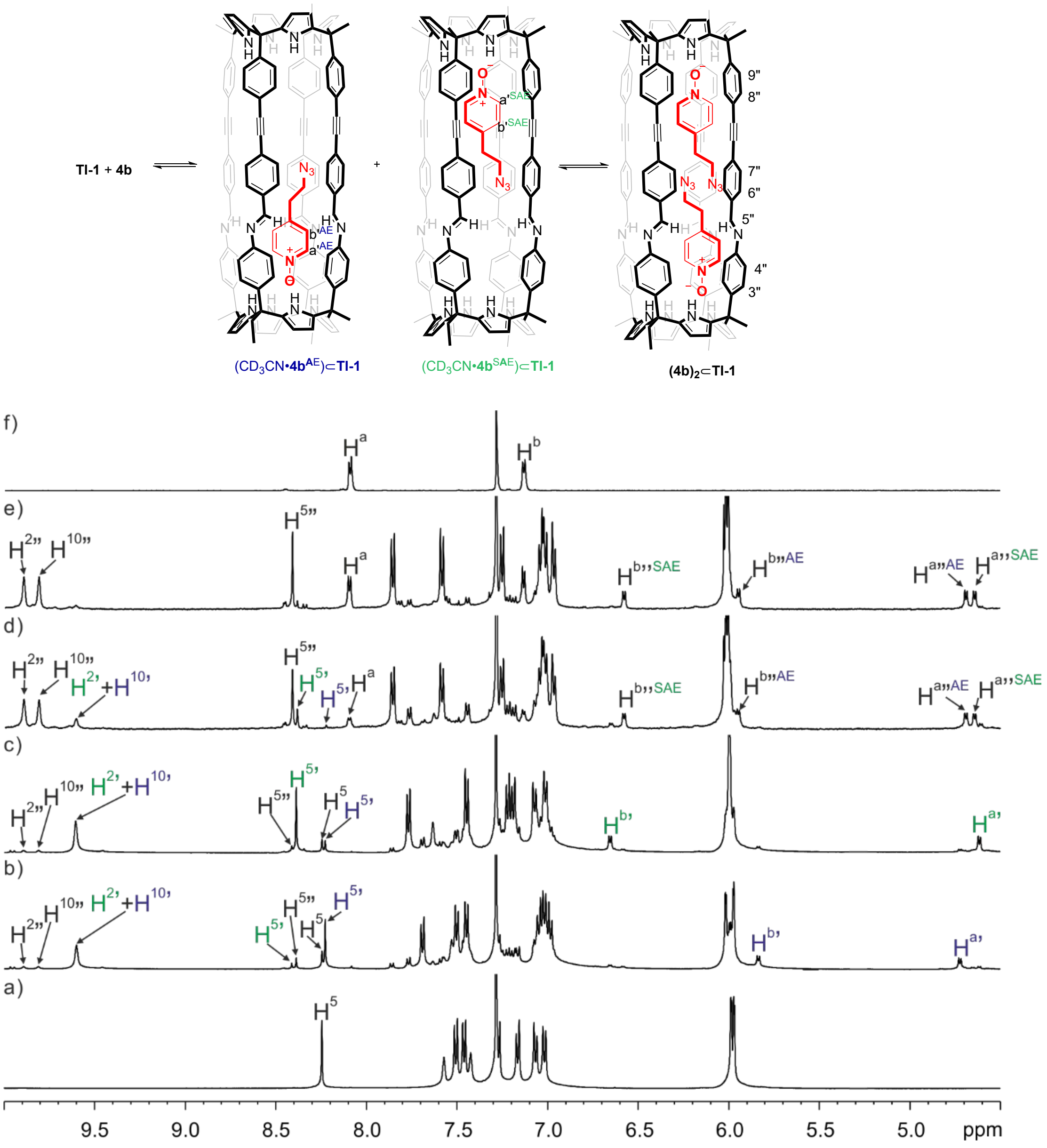

Figure S 15. Top) Binding equilibria of **TI-1** with guest **4b** to produce two regioisomeric 1:1 complexes, and one 2:1 complex. Bottom) Selected region of the ^{1}H NMR (500 MHz, at 298K, $CDCl_3$:CD_3CN 9:1) spectra acquired during the titration of a 2 mM solution of **TI-1** with incremental additions of **4b**: a) 0 equiv, b) 1 equiv immediately after addition, c) 1 equiv after 12 hours, d) 2 equiv, e) 3 equiv. Spectrum f) corresponds to the free **4b** in the same solvent mixture. Primed and double-primed numbers correspond to 1:1 and 2:1 complexes, respectively. Blue and green colors indicate the guest protons at the two possible hemispheres: aryl-extended AE (blue), and super aryl-extended SAE (green).

3.2. Isothermal titration calorimetry

- *ITC of **TI-1** with 4-ethynyl pyridine-N-oxide **5***

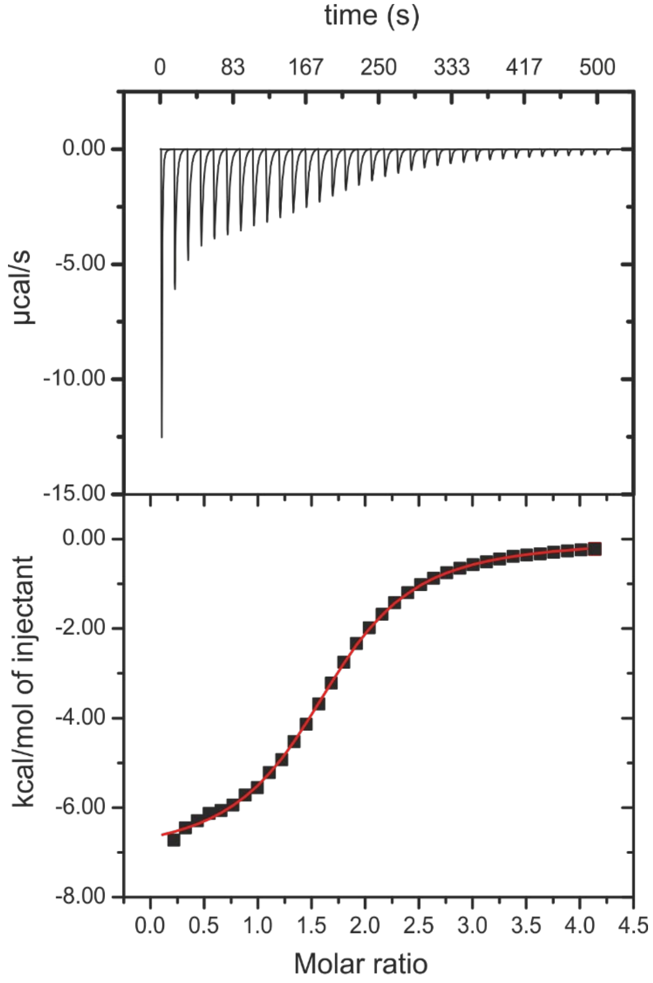

Figure S 16. Top) Traces of the raw data (heat *vs.* time) of the ITC experiment of cage **TI-1** ([cell] = 0.83 mM) with **5** ([syringe] = 16 mM). The solutions were prepared using a $CHCl_3$:CH_3CN 9:1 solvent mixture. Bottom) Normalized integrated heat (black squares) *vs.* **5/TI-1** molar ratio. Experimental data were fit to a one-set-of-sites model implemented in Microcal software (solid red line).

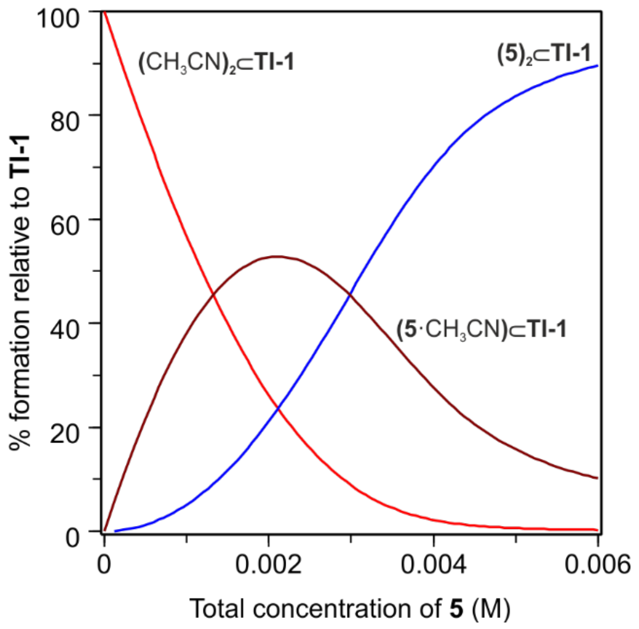

Figure S 17. Theoretical speciation profile of the binding of **5** (up to 3 equiv) to a 2 mM solution of **TI-1,** considering the previously determined binding constants $K_{(1:1)} = 1.8 \times 10^4\ M^{-1}$ and $\beta_{2:1} = 8.3 \times 10^7\ M^{-2}$.

- *ITC of **TI-1** with 4-azido(methyl) pyridine-N-oxide **4a***

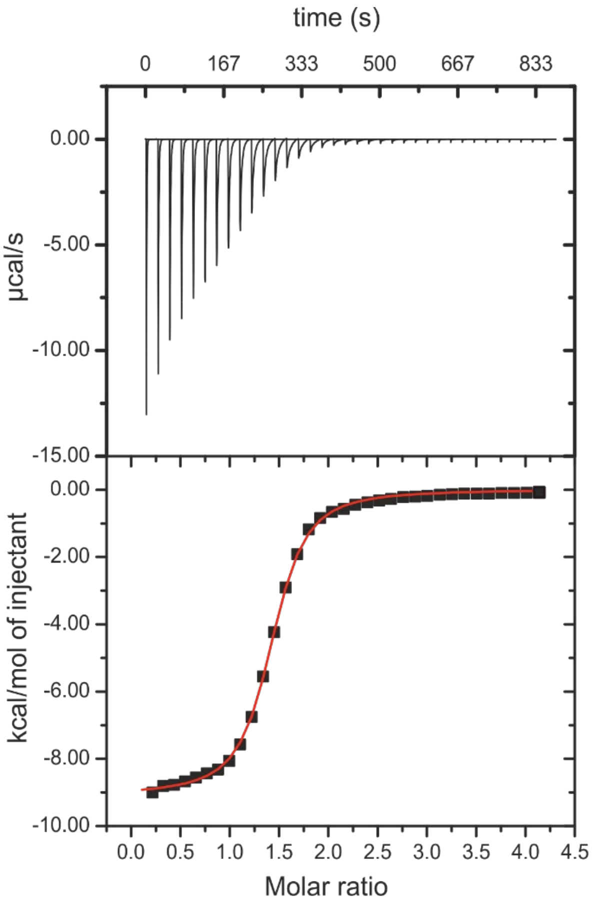

Figure S 18. Top) Traces of the raw data (heat *vs.* time) of the ITC experiment of cage **TI-1** ([cell] = 0.83 mM) with **4a** ([syringe] = 16 mM). The solutions were prepared using a $CHCl_3$:CH_3CN 9:1 solvent mixture. Bottom) Normalized integrated heat (black squares) *vs.* **4a/TI-1** molar ratio. Experimental data were fit to a one-set-of-sites model implemented in Microcal software (solid red line).

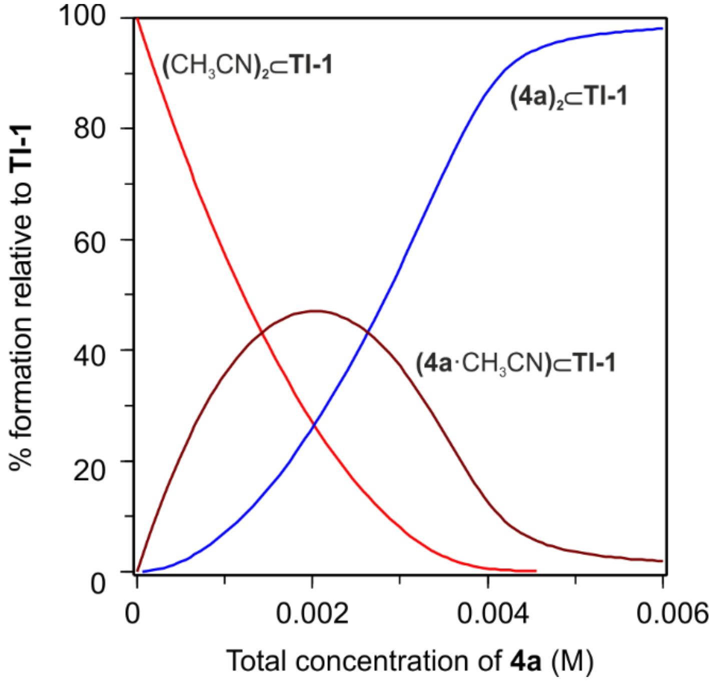

Figure S 19. Theoretical speciation profile of the binding of **4a** (up to 3 equiv) to a 2 mM solution of **TI-1,** considering the previously determined binding constants $K_{(1:1)} = 8.8 \times 10^4$ M^{-1} and $\beta_{2:1} = 2.0 \times 10^9$ M^{-2}.

- *ITC of **TI-1** with 4-azido(ethyl) pyridine-N-oxide **4b***

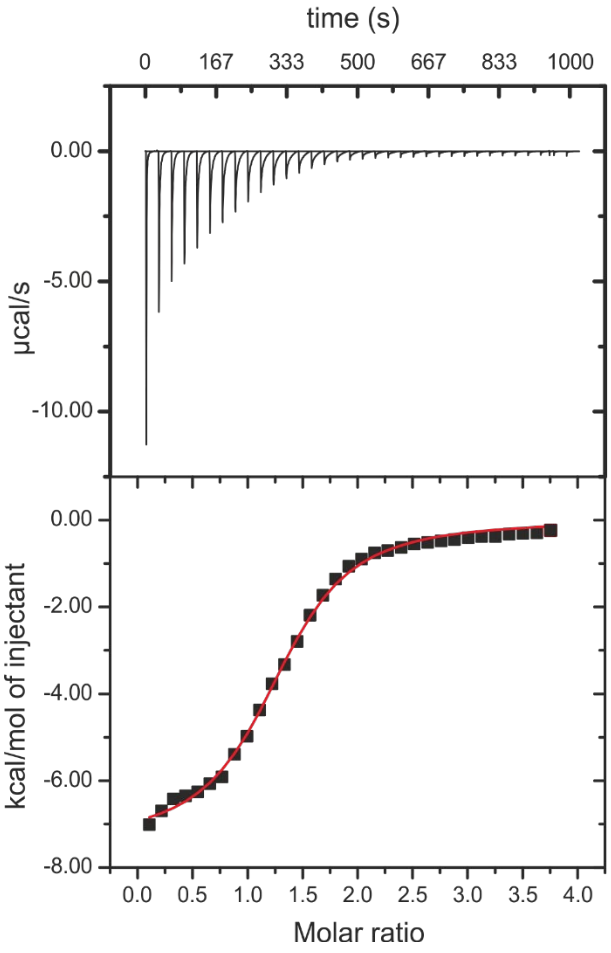

Figure S 20. Top) Traces of the raw data (heat vs. time) of the ITC experiment of cage **TI-1** ([cell] = 0.86 mM) with **4b** ([syringe] = 16 mM). The solutions were prepared using a $CHCl_3$:CH_3CN 9:1 solvent mixture. Bottom) Normalized integrated heat (black squares) vs. **4b/TI-1** molar ratio. Experimental data were fit to a one-set-of-sites model implemented in Microcal software (solid red line).

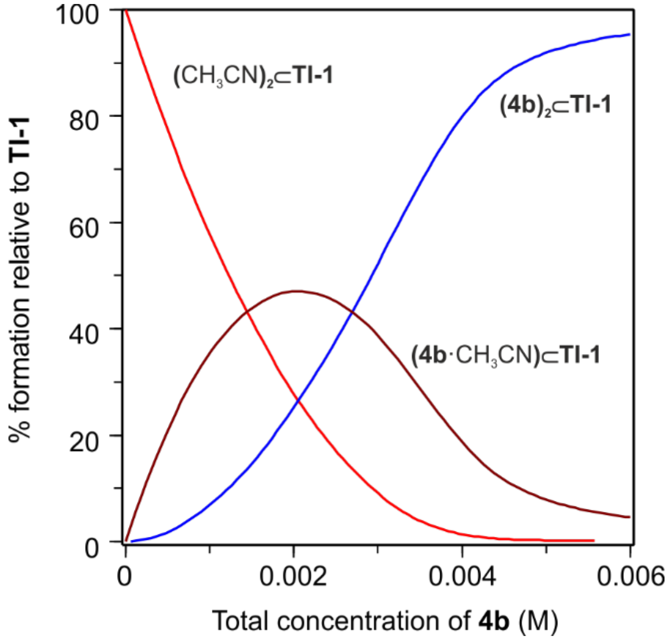

Figure S 21. Theoretical speciation profile of the binding of **4b** (up to 3 equiv) to a 2 mM solution of **TI-1,** considering the previously determined binding constants $K_{(1:1)} = 3.5 \times 10^4\ M^{-1}$ and $\beta_{2:1} = 3.4 \times 10^8\ M^{-2}$.

4. Binding studies of tetra-imine cage TI-1 with ditopic bis-pyridine-*N*-oxide cycloaddition products 6a and 6b.

- *Binding of 1,4-disubstituted triazole guest **6a** with a 2 mM solution of **TI-1** in $CDCl_3$:CD_3CN 9:1 mixture.*

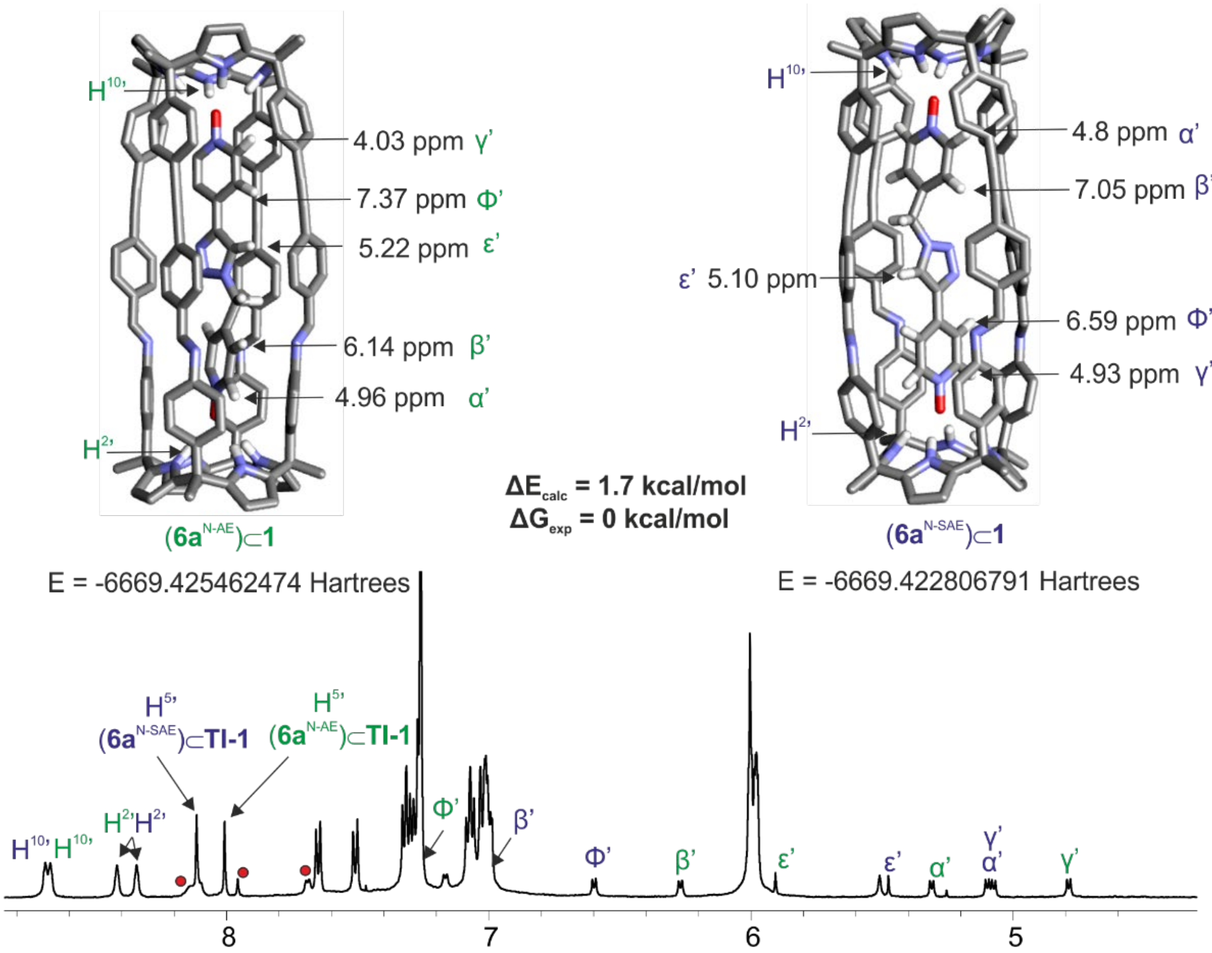

Figure S 22. Top) Energy-minimized structures of the regioisomeric complexes **6a**^{AE}⊂**TI-1** and **6a**^{SAE}⊂**TI-1,** including the values for the computed energy (RI-BP86-def-SV(P) level of theory) and chemical shift of selected proton signals. The DFT-computed chemical shift values for the aromatic protons of the bound N-oxides in the energy-optimized structure of the (**5**)$_2$⊂**TI-1** complex supported the assignment. Bottom) ^{1}H NMR spectrum after equilibration of a solid-liquid extraction experiment of bis-pyridine-*N*-oxide **6a** with a 2 mM solution of **TI-1** in $CDCl_3$:CD_3CN 9:1 mixture. Primed numbers correspond to 1:1 complexes, and blue and green colors discriminate the protons from the two possible regioisomers of the 1:1 complexes, **6a**^{SAE}⊂**TI-1** (blue), and **6a**^{AE}⊂**TI-1** (green). Red circles indicate proton signals of the free **6a**.

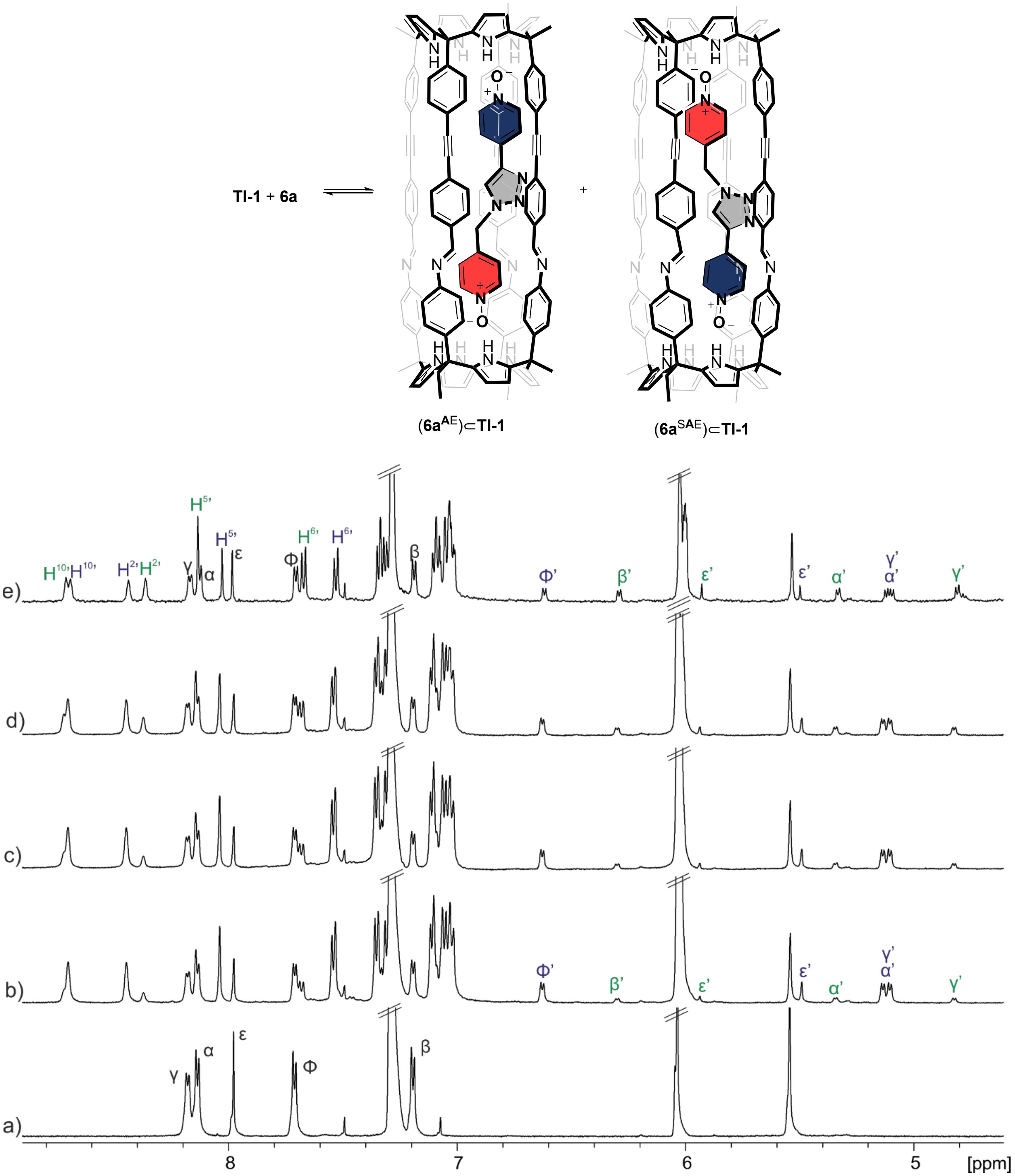

Figure S 23. Top) Binding equilibrium of **6a** and **TI-1** producing two regioisomeric 1:1 complexes. Bottom) Selected downfield region of the ^{1}H NMR spectra (500 MHz, at 298 K, $CDCl_3$:CD_3CN 9:1) of a) 0.8 mM solution of guest **6a**; b) **6a** immediately after addition of 0.5 equiv of **TI-1**, and after 2 h (c), 12h (d), and 4 days (e). Primed numbers correspond to 1:1 complexes. Blue and green colors discriminate the protons from the two possible regioisomers of the 1:1 complexes, **6a**^{SAE}⊂**TI-1** (blue), and **6a**^{AE}⊂**TI-1** (green).

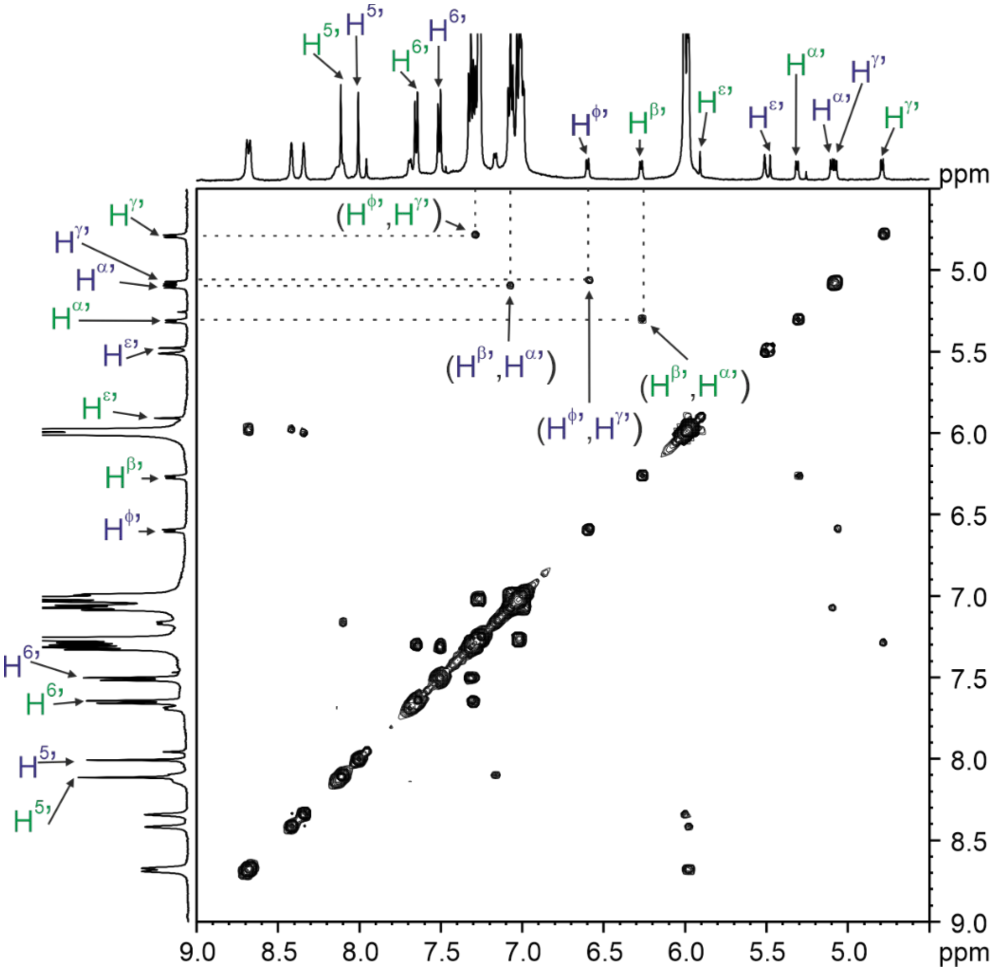

Figure S 24. Selected downfield region of the ^{1}H-^{1}H COSY NMR (500 MHz, at 298 K, $CDCl_3$:CD_3CN 9:1 mixture) spectrum of cage **TI-1** with 2 equiv. of **6a**.

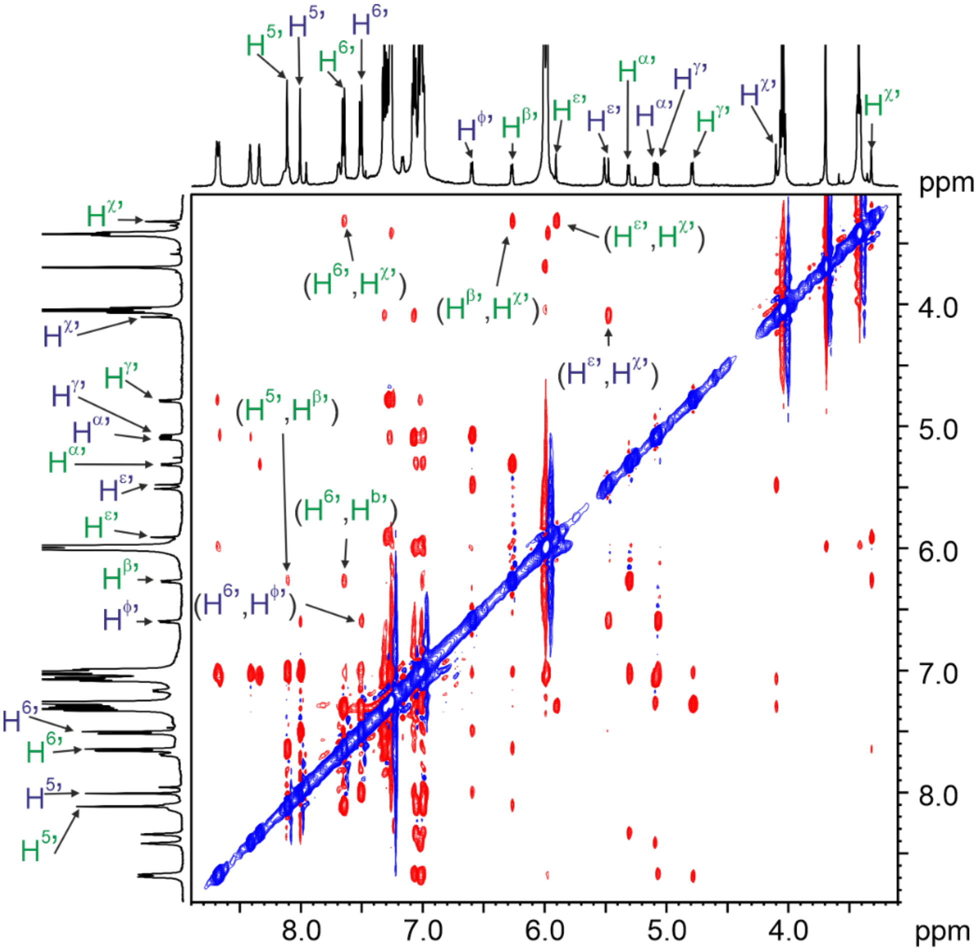

Figure S 25. Selected downfield region of the 2D ^{1}H ROESY NMR (500 MHz, at 298 K, $CDCl_3$:CD_3CN 9:1 mixture, d8 (mixing time) = 300 ms) spectrum of cage **TI-1** with 2 equiv. of **6a**.

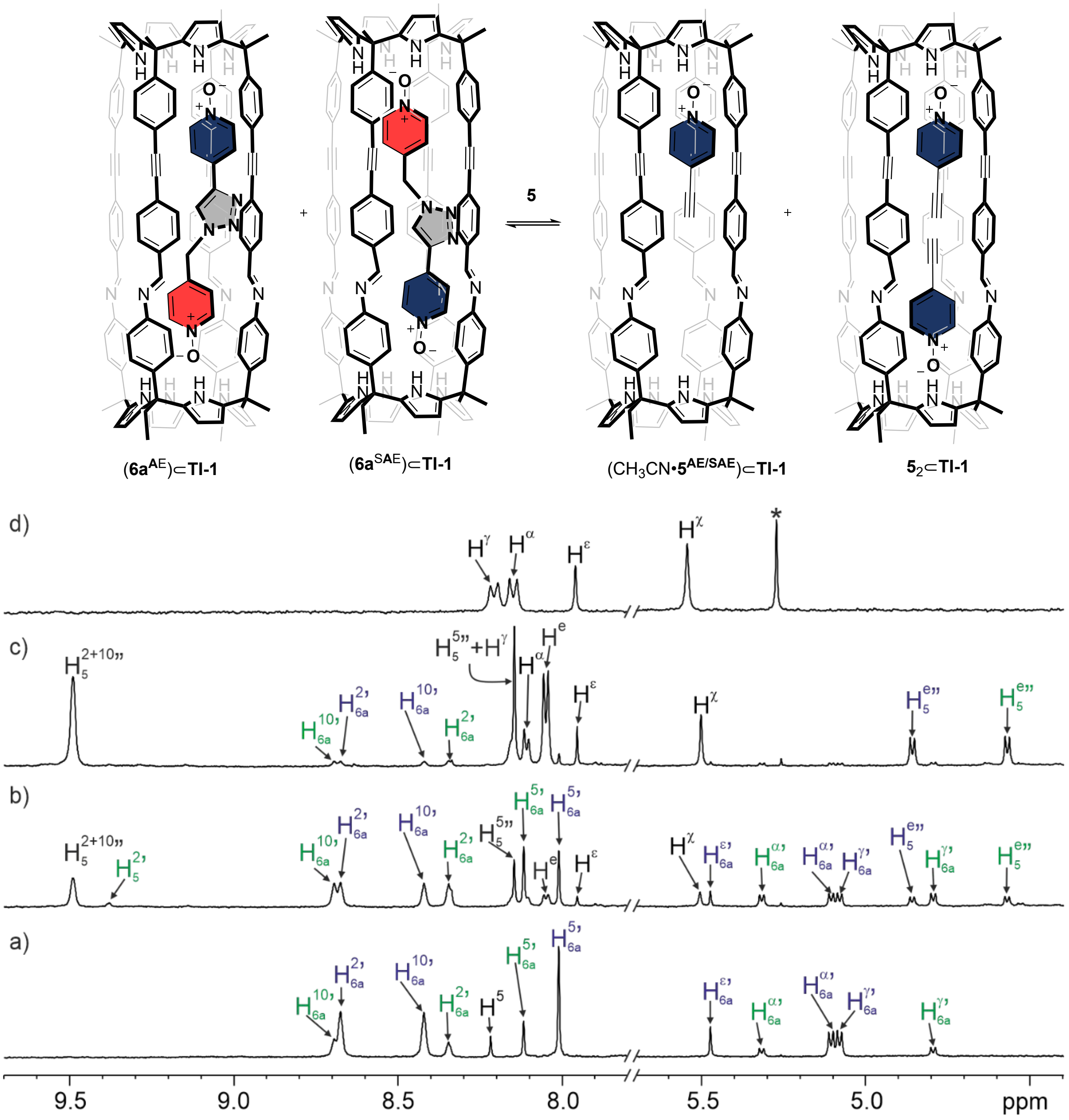

Figure S 26. Top) Binding equilibrium of the competitive experiment of **6a** and **5** for cage **TI-1**. Bottom) Selected downfield region of ^{1}H NMR spectra (500 MHz, 298K, $CDCl_3$:CD_3CN 9:1 mixture) of a) thermally equilibrated 2mM solution of **TI-1** containing 0.9 equiv. of **6a**. Solution a) upon addition of b) 1 equiv. of **5**, and c) 4 equiv. of **5**. Spectrum d) corresponds to that of free **6a** in the same solvent mixture. ^{1}H NMR spectra b and c were measured 1 hour after each addition. Primed and double-primed numbers correspond to 1:1 and 2:1 complexes, respectively. Blue and green colors discriminate the protons from the two possible regioisomers of the 1:1 complexes, (**6a/5**^{SAE})⊂**TI-1** (blue), and (**6a/5**^{AE})⊂**TI-1** (green).

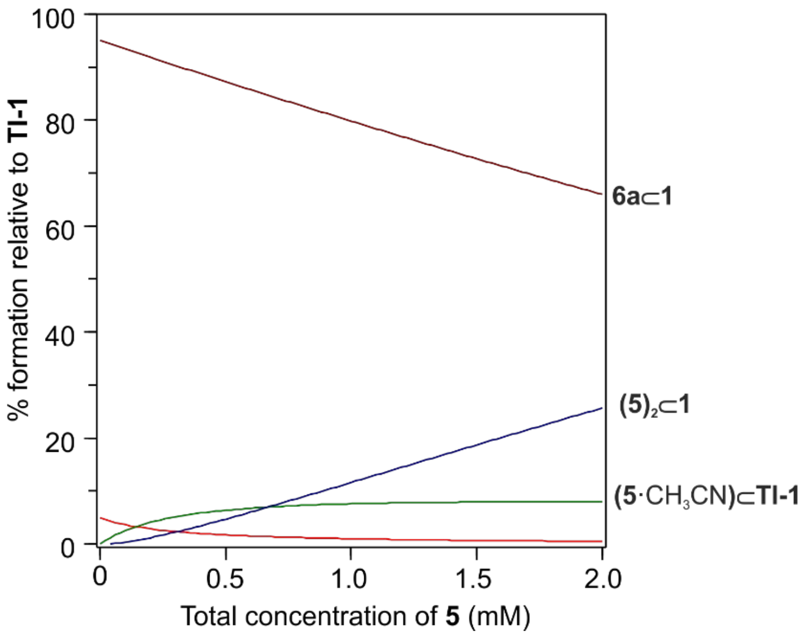

Figure S 27. Theoretical speciation profile of the competitive binding of **6a** and **5** for **TI-1** at 2 mM concentrations, considering the determined binding constants. We also performed the reverse competitive experiment. The addition of 1 equiv of **6a** to a 1:1 mixture of **5** and cage **TI-1** (final concentration 0.7 mM), produced the gradual replacement of the monotopic guests by the ditopic one. The exchange is slow on the human timescale (~12 h). At equilibrium, we quantified a 3:20:77 (CH_3CN•**5**)⊂**TI-1**:(**5**)$_2$⊂**TI-1**:**6a**⊂**TI-1** molar ratio, in agreement with the theoretical speciation profile obtained using the determined and estimated binding constants.

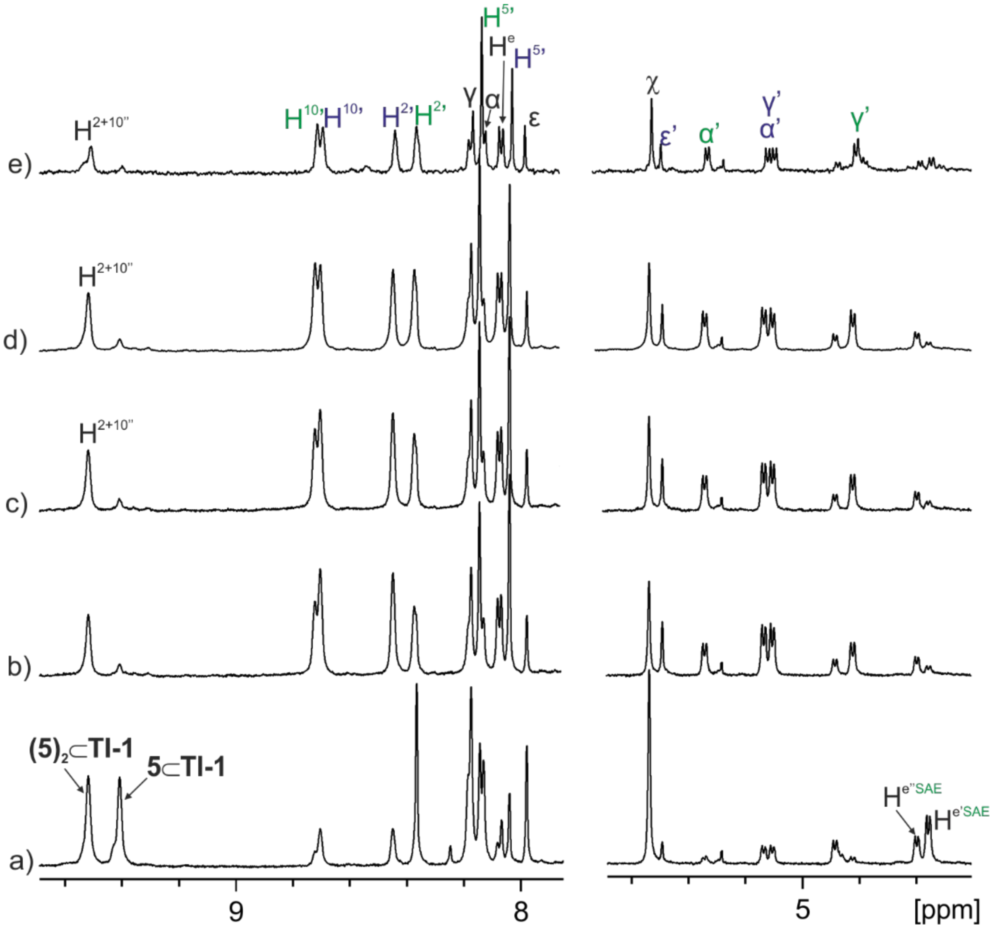

Figure S 28. Selected downfield region of ^{1}H NMR spectra (500 MHz, 298K, $CDCl_3$:CD_3CN 9:1 mixture) of an equimolar 0.8 mM solution of **TI-1**, **5,** and **6b** a) immediately after mixing the compounds and after b) 4h, c) 6h, d) 12h and e) 48 h. Primed and double-primed numbers correspond to 1:1 and 2:1 complexes, respectively. Blue and green colors discriminate the protons from the two possible regioisomers of the 1:1 complexes, (**6a/5**^{SAE})⊂**TI-1** (blue), and (**6a/5**^{AE})⊂**TI-1** (green).

Alternatively, the addition of 1 equiv of **6b** to a 1:1 mixture of **5** and cage **TI-1** (final concentration 1 mM), resulted in the replacement of the monotopic guests by the ditopic one. The exchange is slow on the human timescale. At equilibrium, we exclusively observed the presence of complex **6b⊂TI-1** and free **5** in solution. These results indicated that the binding constant of **6b⊂TI-1** should be larger than $10^{7}M^{-1}$ (EM > 0.1 M).

- *Binding of 1,4-disubstituted triazole guest **6b** with a 2 mM solution of **TI-1** in $CDCl_3$:CD_3CN 9:1 mixture.*

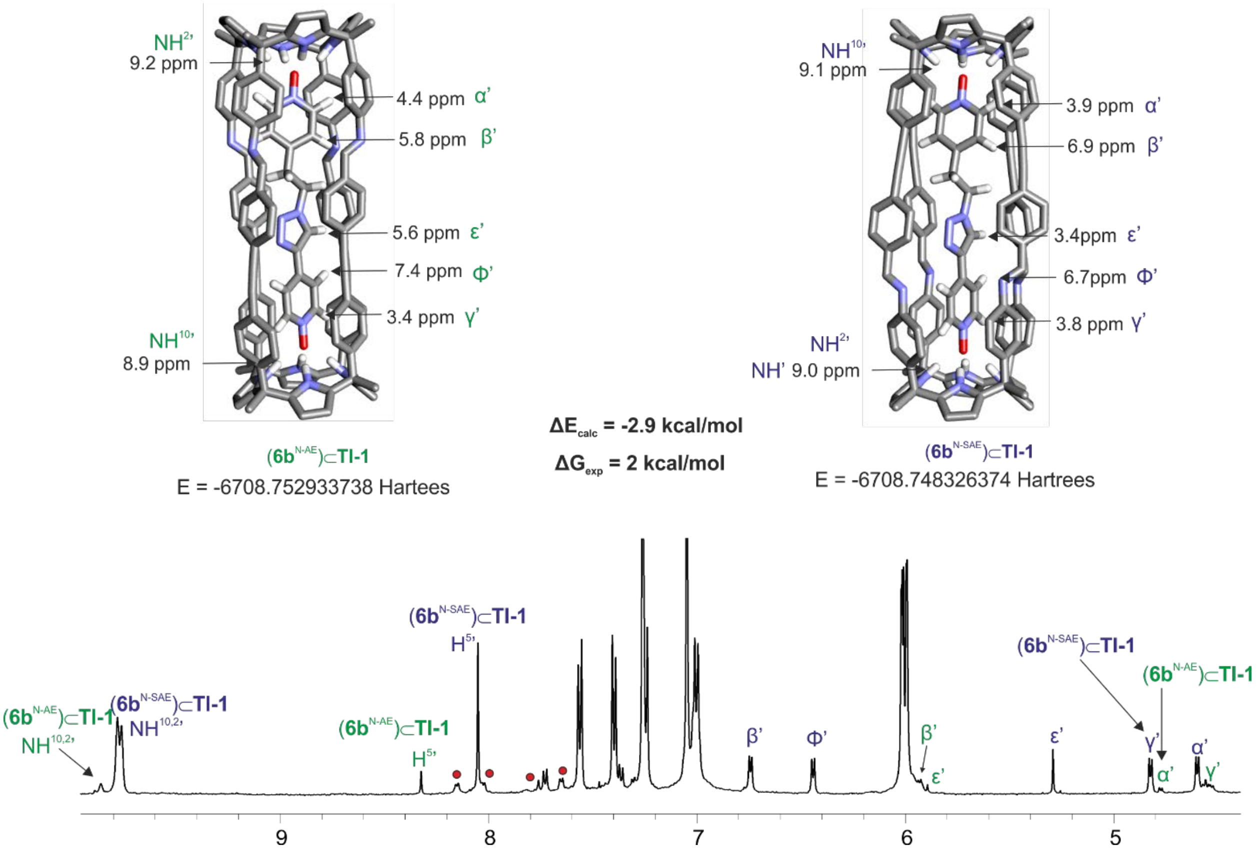

Figure S 29. Top) Energy-minimized structures of the regioisomeric complexes $\mathbf{6b}^{SAE}\subset$**TI-1** and $\mathbf{6b}^{AE}\subset$**TI-1,** including the values for the computed energy (RI-BP86-def-SV(P) level of theory) and chemical shift of selected proton signals. The DFT-computed chemical shift values for the aromatic protons of the bound N-oxides in the energy-optimized structure of the $(\mathbf{5})_2\subset$**TI-1** complex supported the assignment. Bottom) ^{1}H NMR spectrum after solid-liquid extraction experiment of bis-pyridine-*N*-oxide **6b** with a 2 mM solution of **TI-1** in $CDCl_3$:CD_3CN 9:1 mixture. The solution was equilibrated for 24 h. Primed numbers correspond to 1:1 complexes, and blue and green colors discriminate the protons from the two possible regioisomers of the 1:1 complexes, $\mathbf{6b}^{SAE}\subset$**TI-1** (blue), and $\mathbf{6b}^{AE}\subset$**TI-1** (green). Red circles indicate proton signals of the free **6a**.

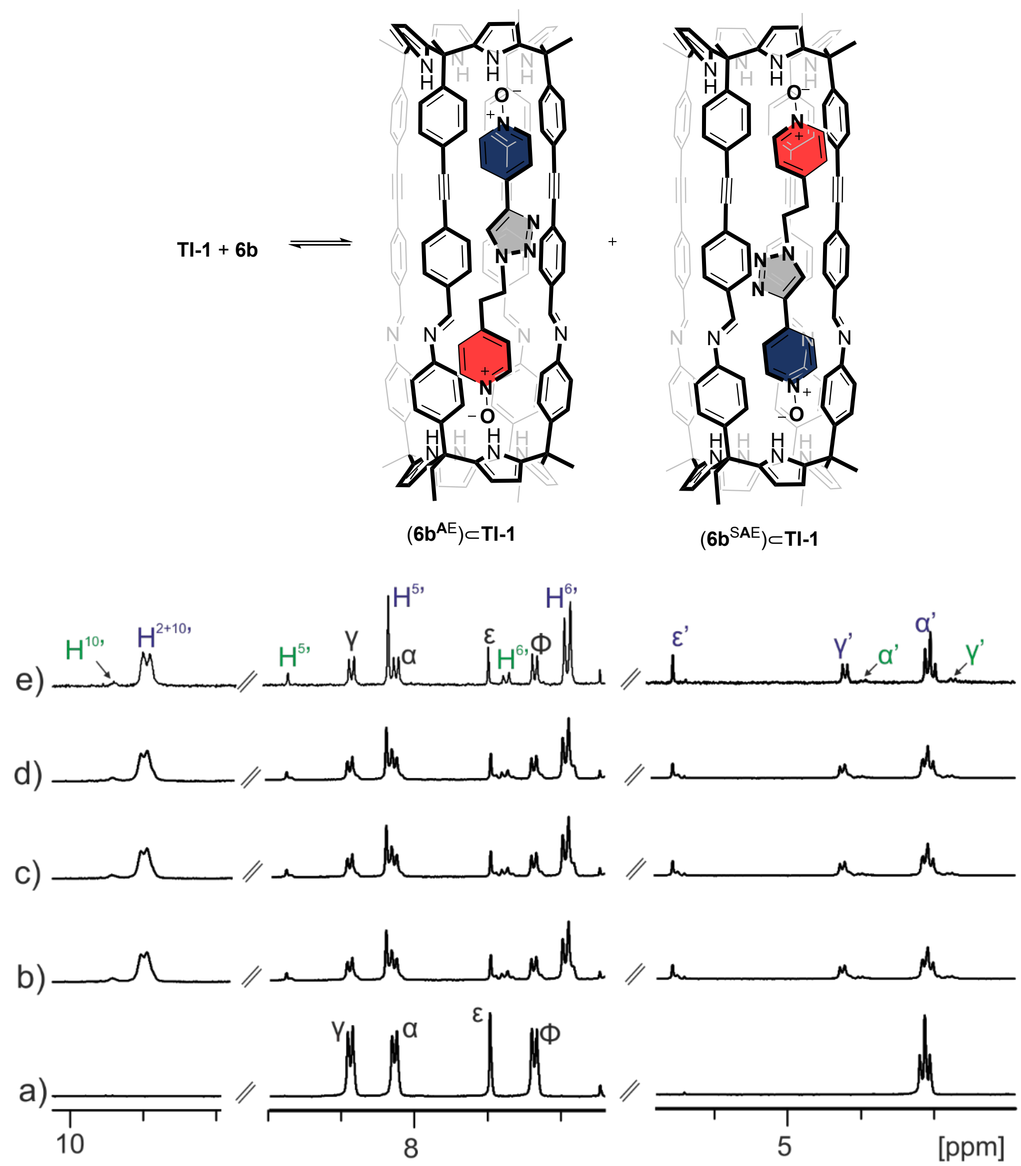

Figure S 30. Top) Binding equilibrium of **6b** and **TI-1** producing two regioisomeric 1:1 complexes. Bottom) Selected downfield region of the ^{1}H NMR spectra (500 MHz, at 298 K, $CDCl_3$:CD_3CN 9:1) of a) 1.1 mM solution of guest **6b**; b) **6b** immediately after addition of 0.5 equiv of **TI-1**, and after 12 h (c), 4 days (d), and 2 weeks (e). Primed numbers correspond to 1:1 complexes. Blue and green colors discriminate the protons from the two possible regioisomers of the 1:1 complexes, **6b**^{SAE}⊂**TI-1** (blue), and **6b**^{AE}⊂**TI-1** (green).

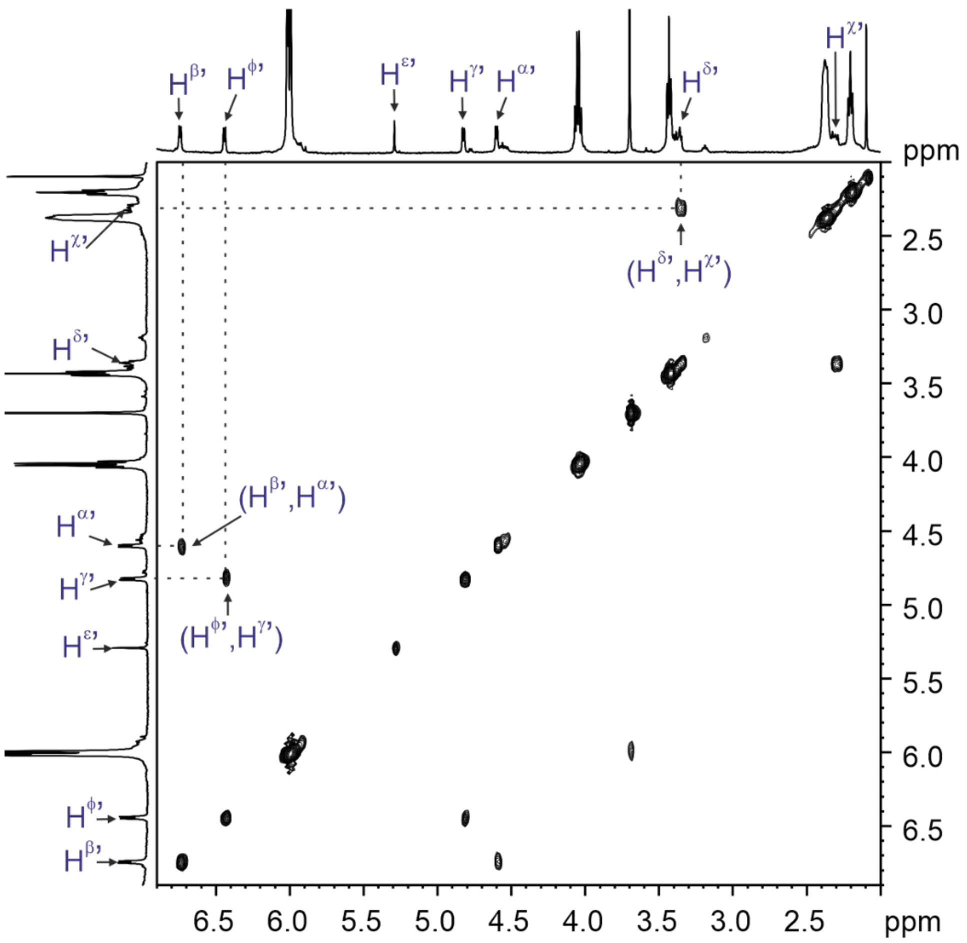

Figure S 31. Selected region of the ^1H-^1H COSY NMR (500 MHz, at 298 K, $CDCl_3$:CD_3CN 9:1 mixture) spectrum of cage **TI-1** with 2 equiv. of **6b**.

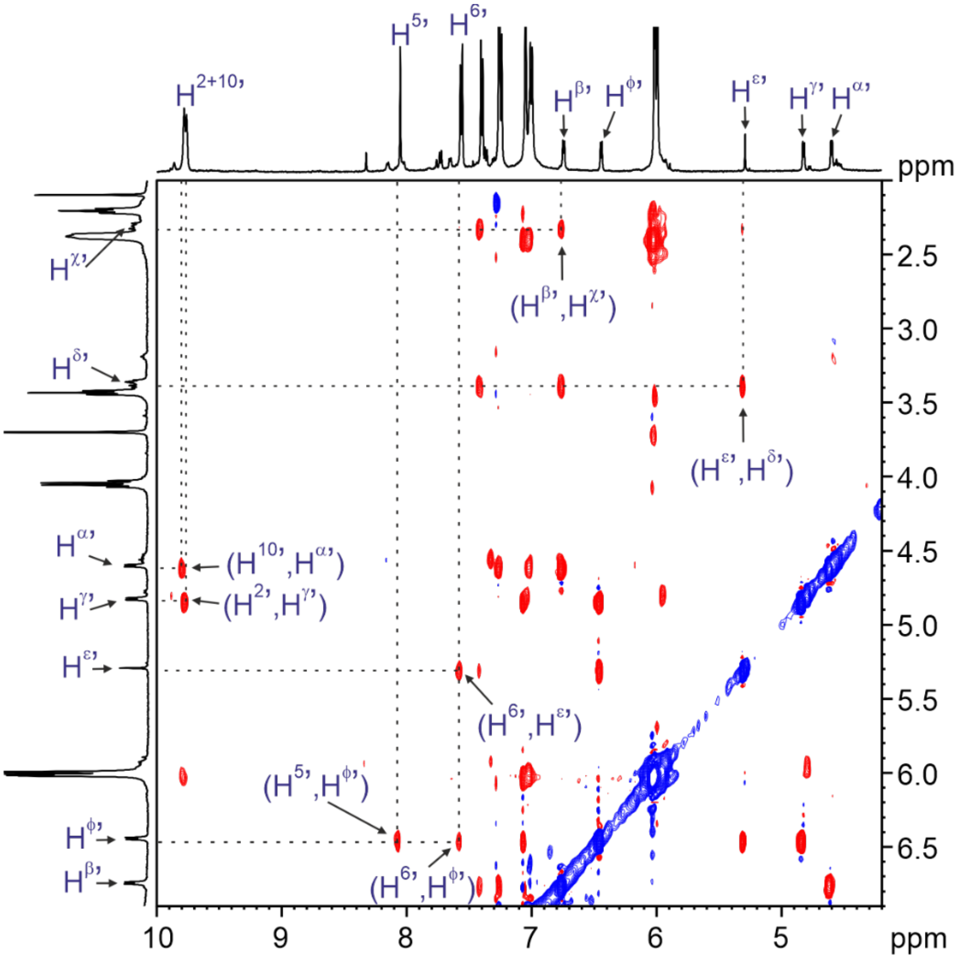

Figure S 32. Selected region of the 2D ^{1}H ROESY NMR (500 MHz, at 298 K, $CDCl_3$:CD_3CN 9:1 mixture, d8 (mixing time) = 300 ms) spectrum of cage **TI-1** with 2 equiv. of **6b**.

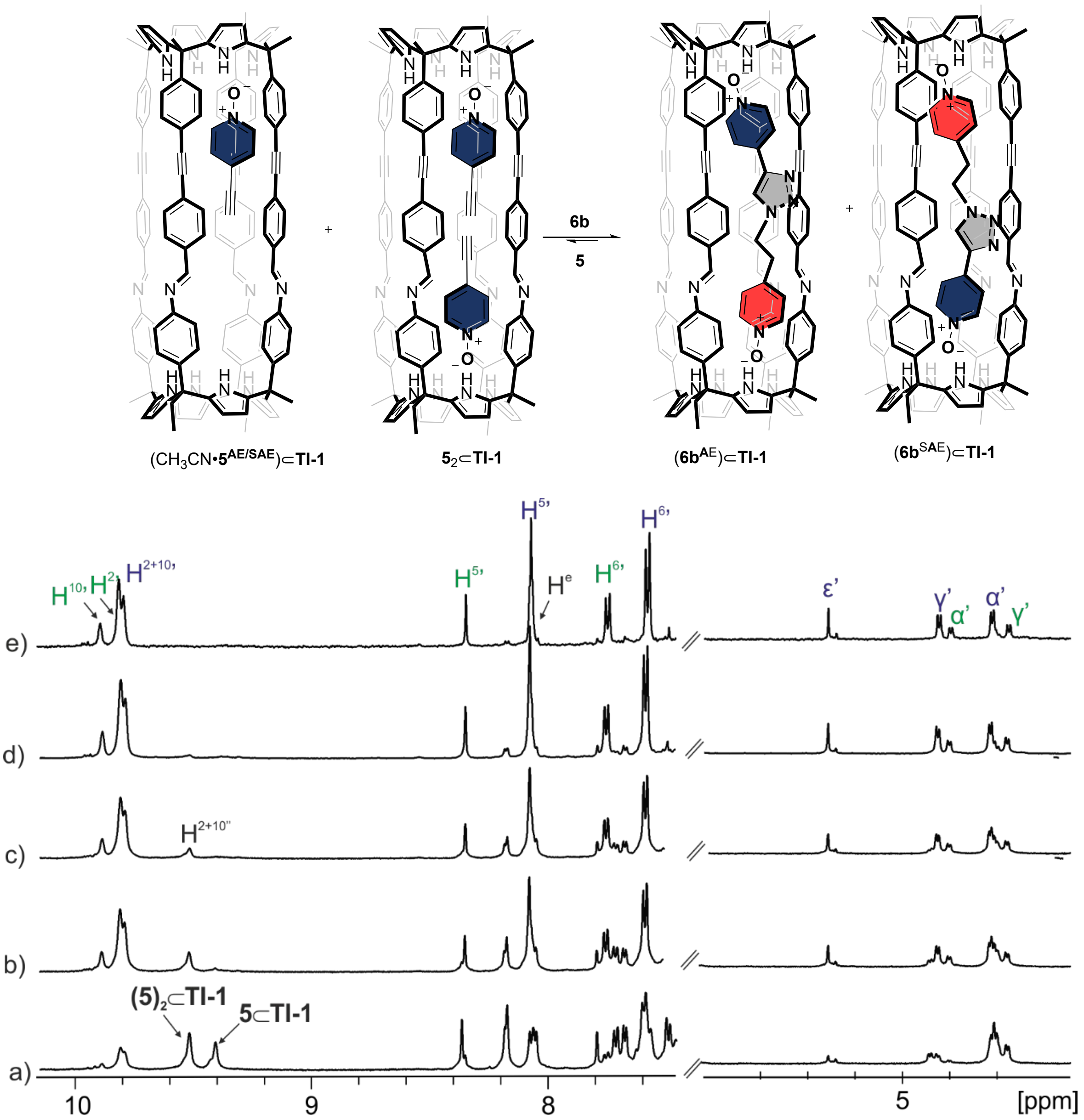

Figure S 33. Top) Binding equilibrium of the competitive experiment of **6b** and **5** for cage **TI-1**. Bottom) Selected downfield region of ^{1}H NMR spectra (500 MHz, 298K, $CDCl_3$:CD_3CN 9:1 mixture) of an equimolar 1.0 mM solution of **TI-1**, **5,** and **6b** a) immediately after mixing the compounds and after b) 1h, c) 2h, d) 12h and e) 48 h. Primed and double-primed numbers correspond to 1:1 and 2:1 complexes, respectively. Blue and green colors discriminate the protons from the two possible regioisomers of the 1:1 complexes, **6b**^{SAE}**⊂TI-1** (blue), and **6b**^{AE}**⊂TI-1** (green).

5. Kinetic characterization of cycloaddition reactions inside TI-1

5.1. Cycloaddition reaction of 4a with 5

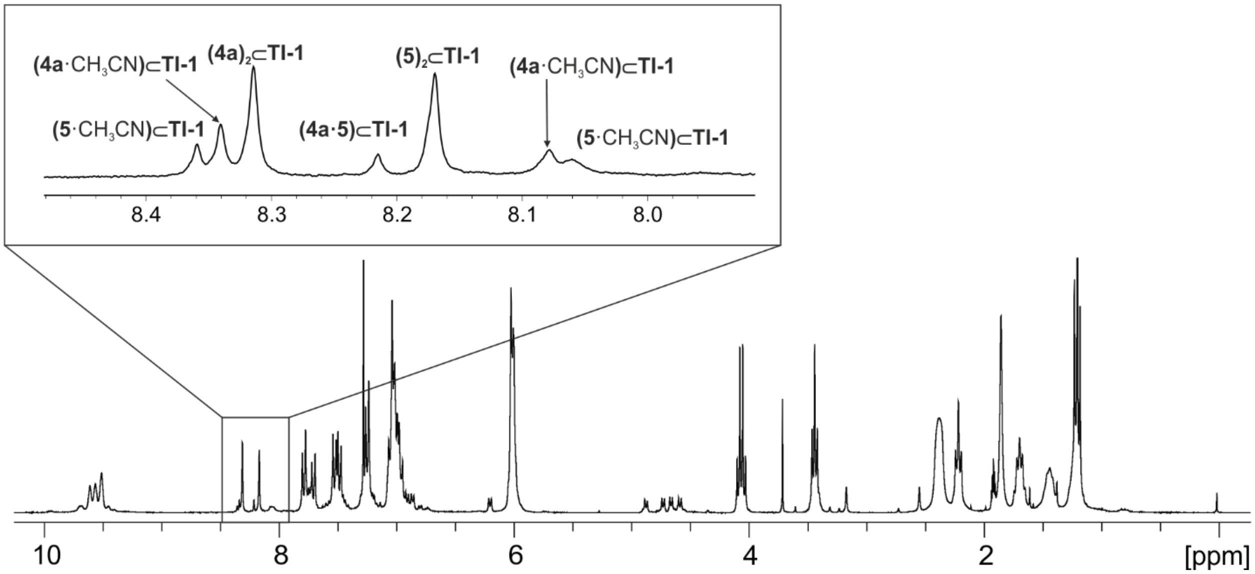

Figure S 34. ^{1}H NMR spectrum of a 1:1:1 mixture of **4a**, **5**, and **TI-1** in $CDCl_3$:CD_3CN 9:1 acquired immediately after the mixture.

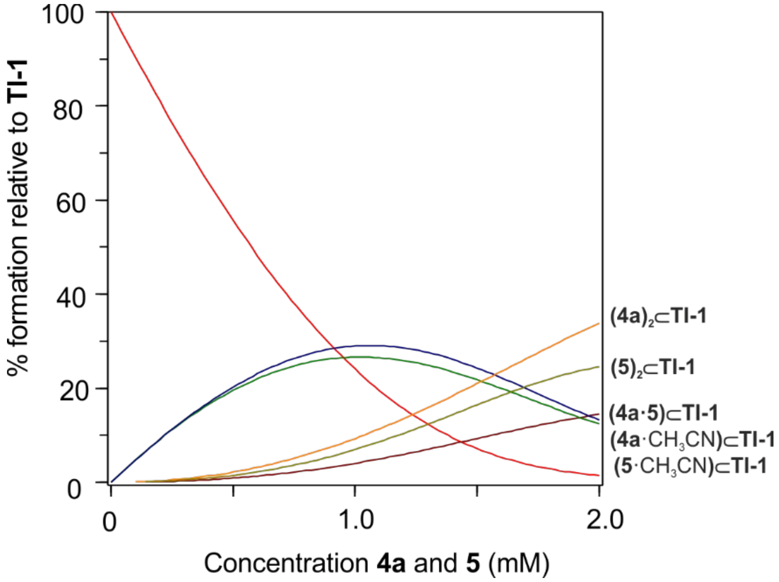

Figure S 35. Simulated speciation profile for the binding of **4a** and **5** (equimolar) to a 2 mM solution of **TI-1**, determined using Hyperquad Simulation and Speciation software (HySS2009) using the model that considers the reversible formation of 1:1 and 2:1 homo- and hetero-inclusion complexes with the constants determined using ITC and ^{1}H NMR binding studies. Regioisomeric complexes were treated as a single species and were not distinguished as separate entities in the model.

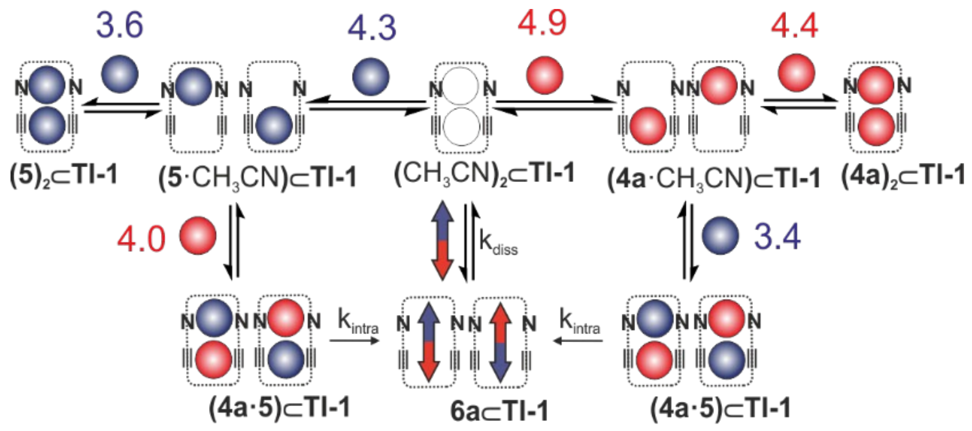

Figure S 36. Theoretical kinetic model used for the non-linear analysis of the experimental data from the mediation of the reaction between **4a** and **5.** The thermodynamic equilibrium constants for each binding process are included. Red spheres correspond to azido-substrate **4a** and blue spheres to ethynyl substrate **5.** Regioisomeric complexes were treated as a single species and were not distinguished as separate entities in the model.

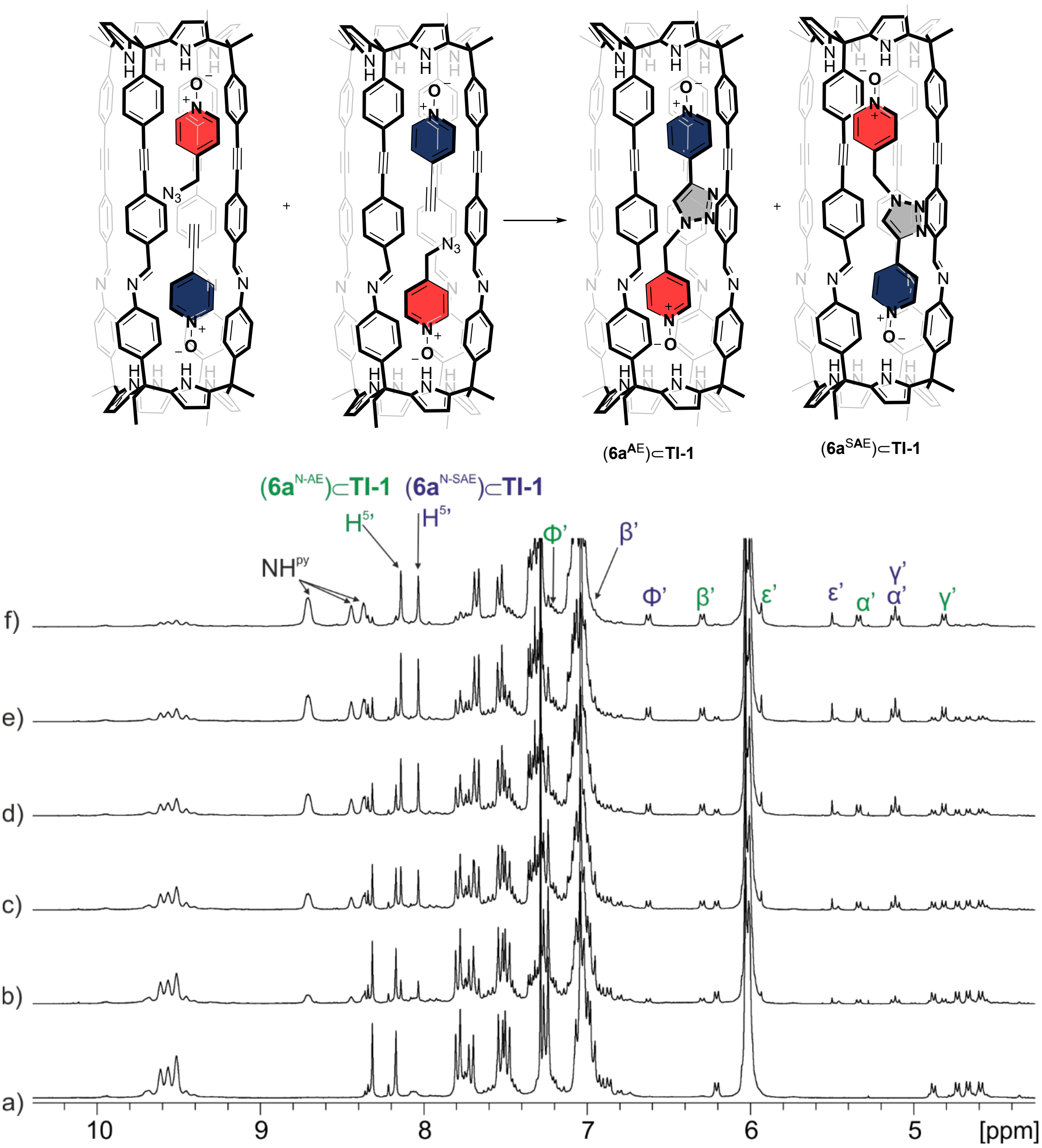

Figure S 37. Top) Dipolar cycloaddition reaction between **4a** and **5** included in **TI-1** to yield **6a⊂TI-1** complex. Bottom) Selected region of the ^{1}H NMR spectra corresponding to the monitoring of the formation of complex **6a⊂TI-1** from an equimolar 2 mM mixture of **TI-1**, **4a**, and **5** in $CDCl_3$:CD_3CN 9:1 solvent mixture. a) 0 days, b) 10 days, c) 20 days, d) 30 days, e) 40 days, and f) 70 days. Primed numbers correspond to 1:1 complexes, and blue and green colors discriminate the protons from the two possible regioisomers of the 1:1 complexes, **6a^{SAE}⊂TI-1** (blue), and **6a^{AE}⊂TI-1** (green).

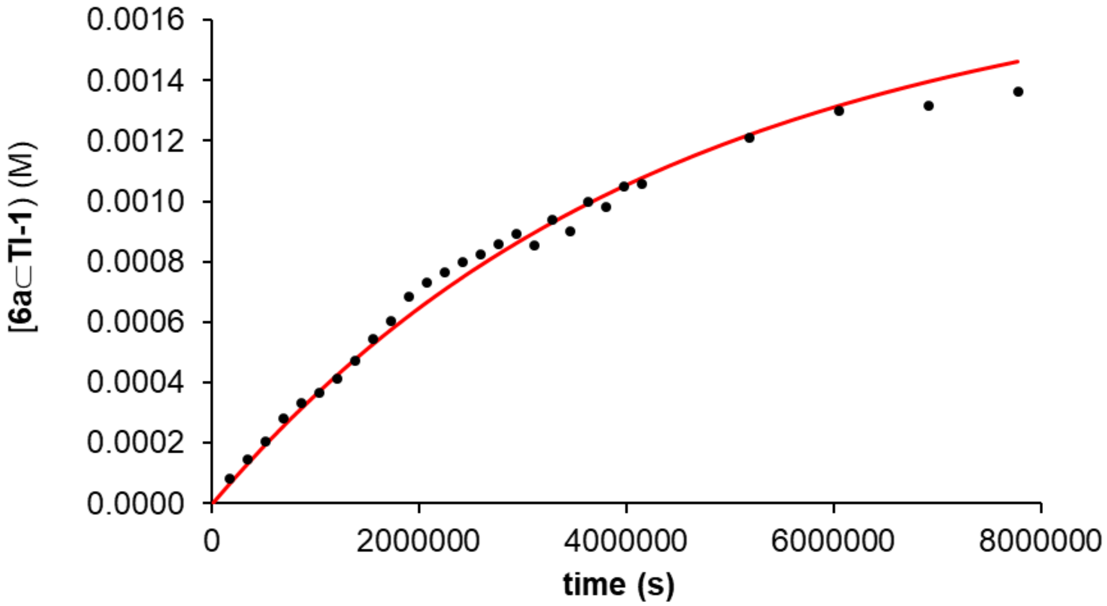

Figure S 38. Change in the concentration of the sum of two regioisomeric complexes **6a⊂TI-1** (black dots) with time, starting from a 1:1:1 mixture of **TI-1**, **4a** and **5** (2 mM). Solid red line represents the fit of the experimental data to the theoretical kinetic model using the parameters estimation module of COPASI software version 4.25. The k_{on}/k_{off} ratio of all the binding equilibria were manually fixed based on the estimated k_{off} and the determined thermodynamic binding constants. k_{intra} was the only variable parameter used for the fit k_{intra} = 1.99 (± 0.2) × 10^{-6} s^{-1} (from two kinetic experiments).

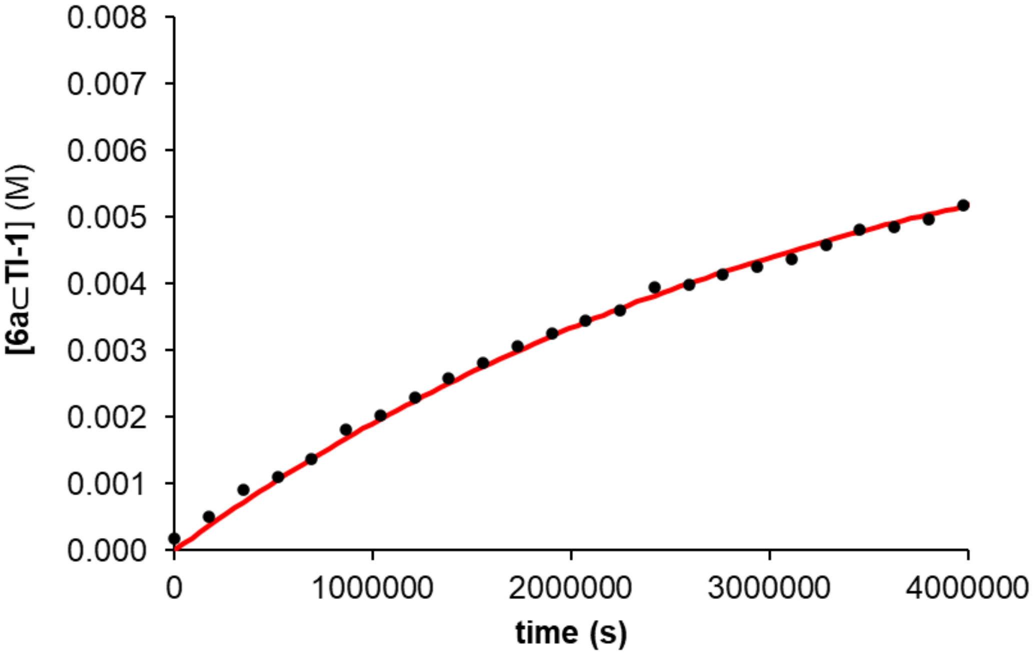

Figure S 39. Change in the total concentration of the two regioisomeric complexes **6a⊂TI-1** (black dots) with time, starting from a 1:1:1 mixture of **TI-1**, **4a** and **5** (8 mM). Solid red line represents the fit of the experimental data to the theoretical kinetic model using the parameters estimation module of COPASI software version 4.25. The k_{on}/k_{off} ratio of all the binding equilibria were manually fixed based on the estimated k_{off} and the determined thermodynamic binding constants. k_{intra} was the only variable parameter used for the fit $k_{intra} = 2.14 \times 10^{-6}$ s^{-1}.

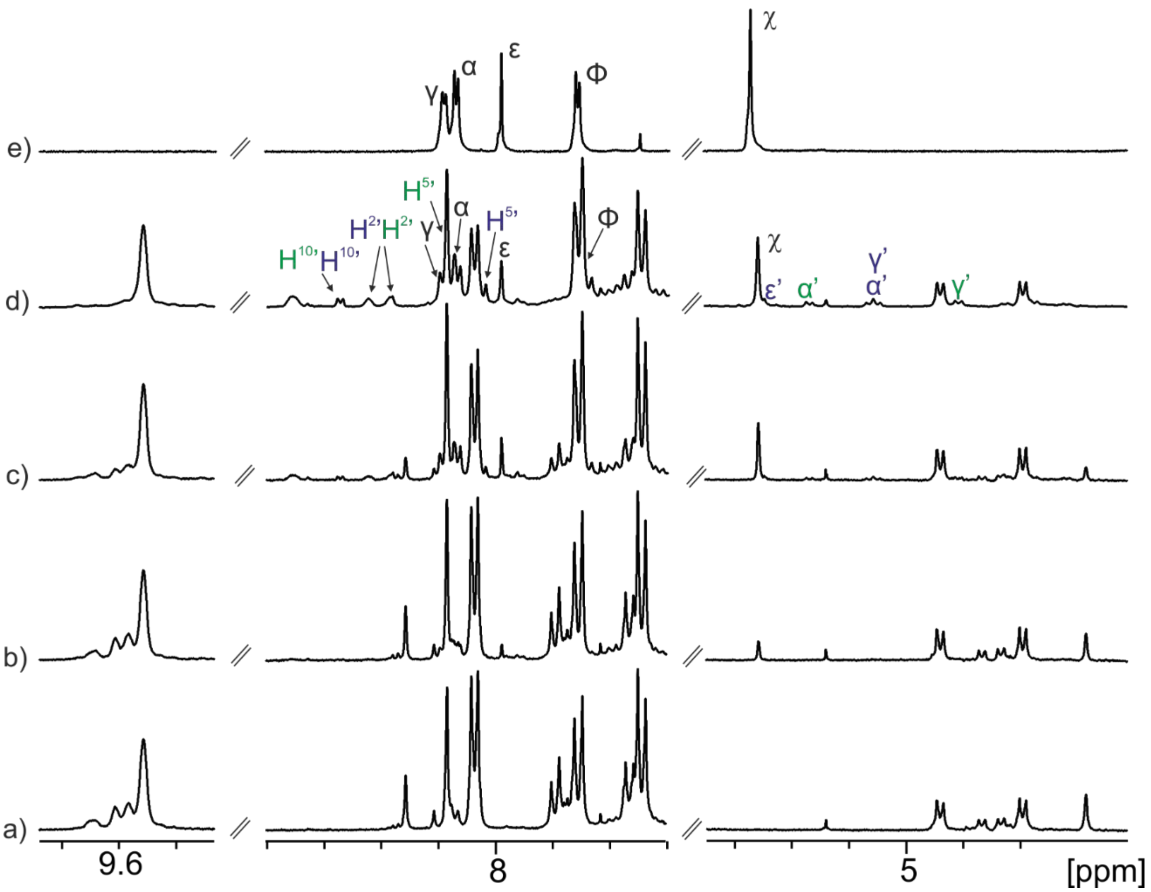

Figure S 40. Selected region of the ^{1}H NMR spectra corresponding to the monitoring of the formation of **6a** from a 1:1:4 mixture of **TI-1** (2 mM), **4a** (2 mM), and **5** (8 mM) in $CDCl_3$:CD_3CN 9:1 solvent mixture. a) 0 days, b) 5 days, c) 20 days, and d) 70 days. e) 1H NMR spectrum of free 6a in the same solvent mixture. Primed numbers correspond to 1:1 complexes, and blue and green colors discriminate the protons from the two possible regioisomers of the 1:1 complexes, **6a**^{SAE}⊂**TI-1** (blue), and **6a**^{AE}⊂**TI-1** (green).

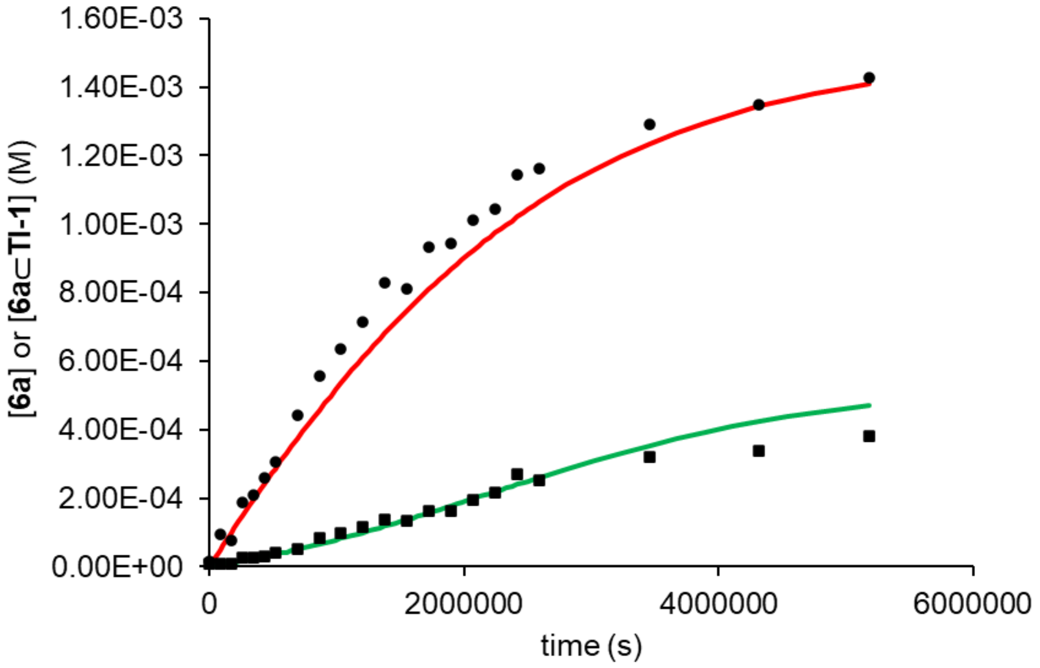

Figure S 41. Change in the concentration of the two regioisomeric complexes **6a⊂TI-1** (black dots) and free **6a** (black squares) with time, starting from a 1:1:4 2 mM mixture of **TI-1**, **4a** and **5**. Solid red and green lines represent the fit of the experimental data to the theoretical kinetic model using the parameters estimation module of COPASI software version 4.25. The k_{on}/k_{off} ratio of all the binding equilibria were manually fixed based on the estimated k_{off} and the determined thermodynamic binding constants. k_{intra} was the only variable parameter used for the fit k_{intra} = 2.15×10^{-6} s^{-1}.

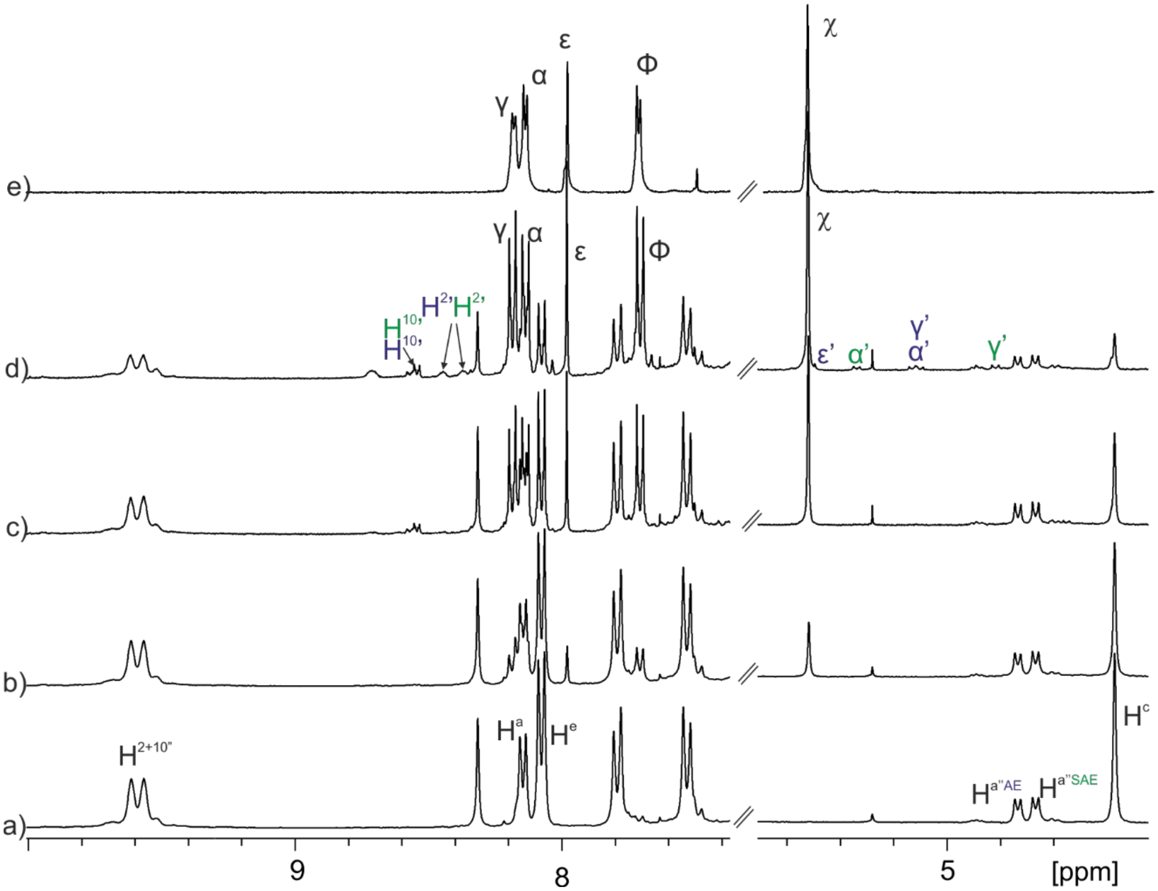

Figure S 42. Selected region of the ^{1}H NMR spectra corresponding to the monitoring of the formation of **6a** from a 1:4:4 mixture of **TI-1** (2 mM), **4a** (8 mM), and **5** (8 mM) in $CDCl_3$:CD_3CN 9:1 solvent mixture. a) 0 days, b) 40 days, c) 120 days, and d) 180 days. e) ^{1}H NMR spectrum of free **6a** in the same solvent mixture. Primed and double-primed numbers correspond to 1:1 and 2:1 complexes, respectively, and blue and green colors discriminate the protons from the two possible regioisomers of the 1:1 complexes, **6a**^{SAE}⊂**TI-1** (blue), and **6a**^{AE}⊂**TI-1** (green).

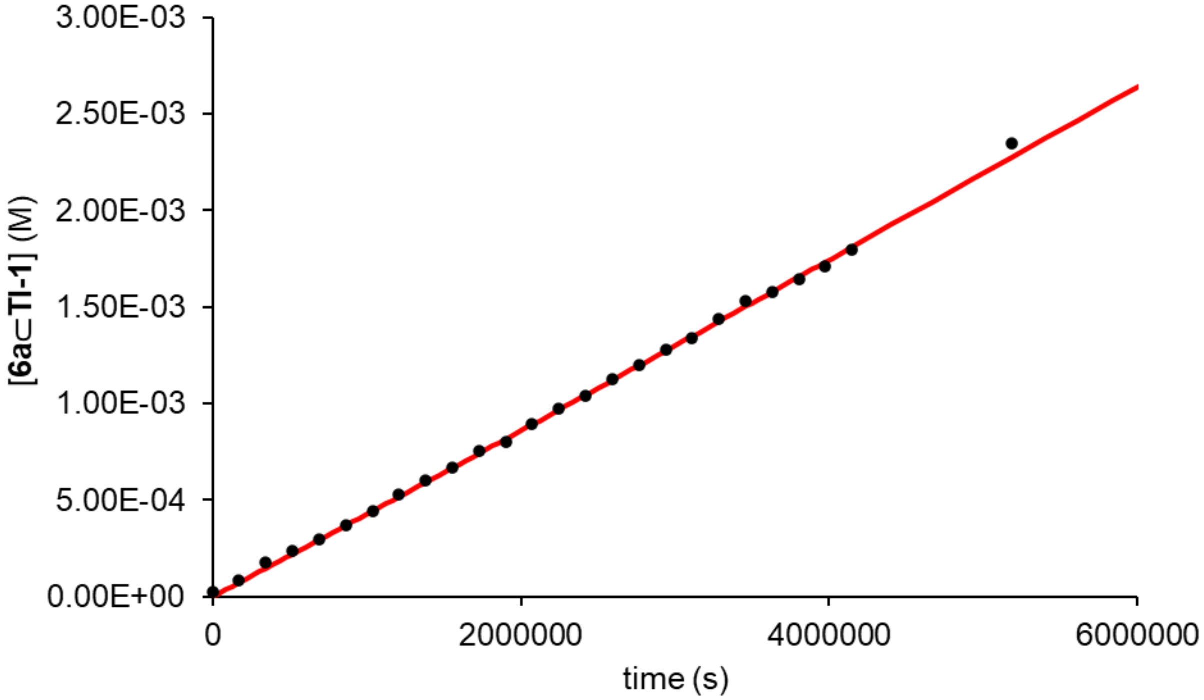

Figure S 43. Change in the concentration of the two regioisomeric complexes **6a⊂TI-1** (black dots) with time, starting from a 1:4:4 2 mM mixture of **TI-1**, **4a** and **5**. Solid red line represents the fit of the experimental data to the theoretical kinetic model using the parameters estimation module of COPASI software version 4.25. The k_{on}/k_{off} ratio of all the binding equilibria were manually fixed based on the estimated k_{off} and the determined thermodynamic binding constants. k_{intra} was the only variable parameter used for the fit k_{intra} = 1.85 (± 0.3) × 10^{-6} s^{-1}.

5.2. Cycloaddition reaction of 4b with 5

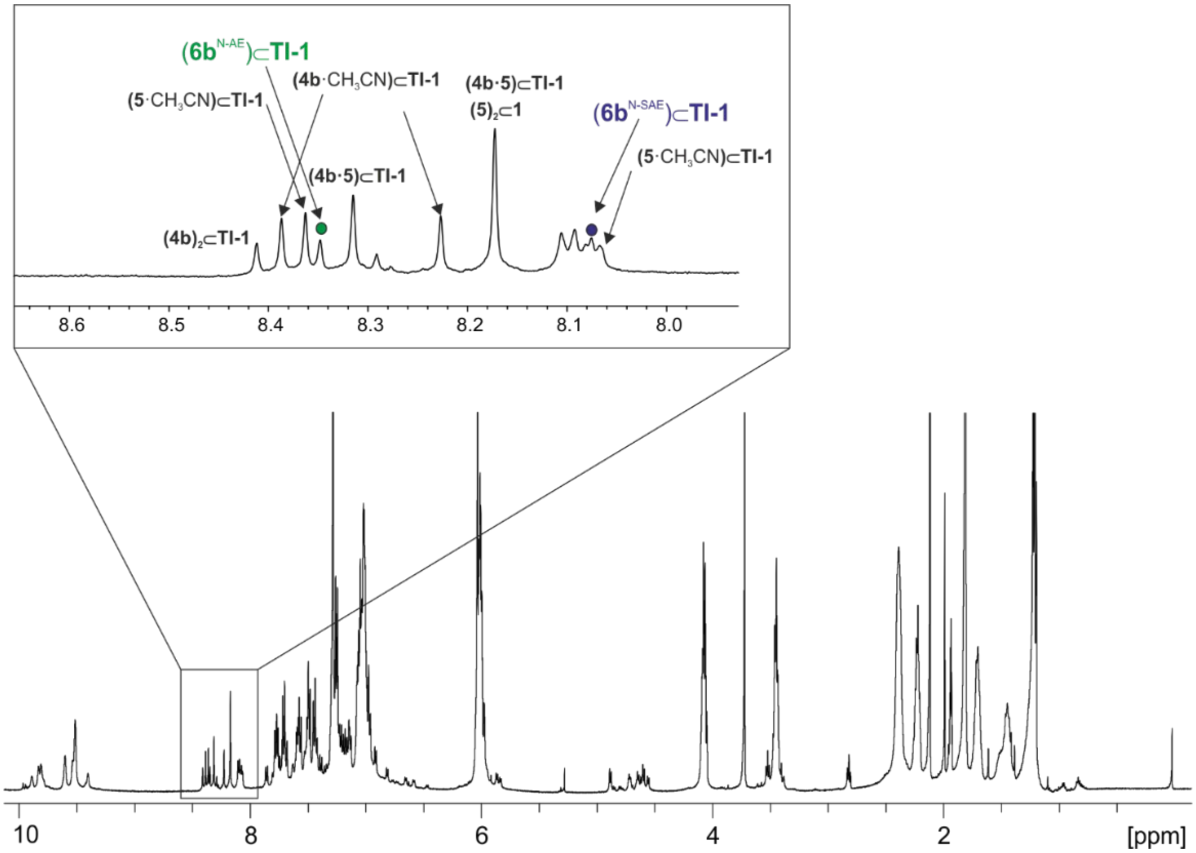

Figure S 44. ^{1}H NMR spectrum of a 1:1:1 mixture of **4b**, **5**, and **TI-1** in $CDCl_3$:CD_3CN 9:1 acquired immediately after the mixture.

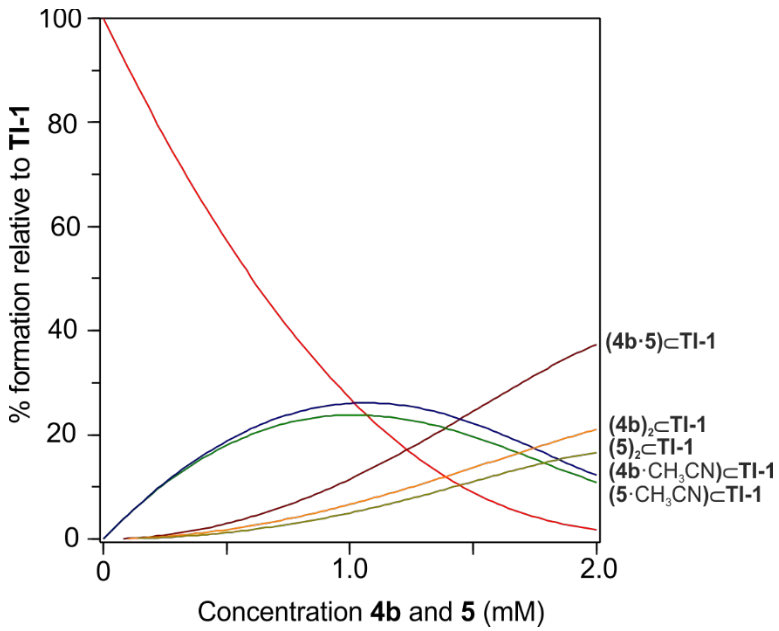

Figure S 45. Simulated speciation profile for the binding of **4b** and **5** (equimolar) to a 2 mM solution of **TI-1**, determined using Hyperquad Simulation and Speciation software (HySS2009) using the model that considers the reversible formation of 1:1 and 2:1 homo- and hetero-inclusion complexes with the constants determined using ITC and ^{1}H NMR binding studies. Regioisomeric complexes were treated as a single species and were not distinguished as separate entities in the model.

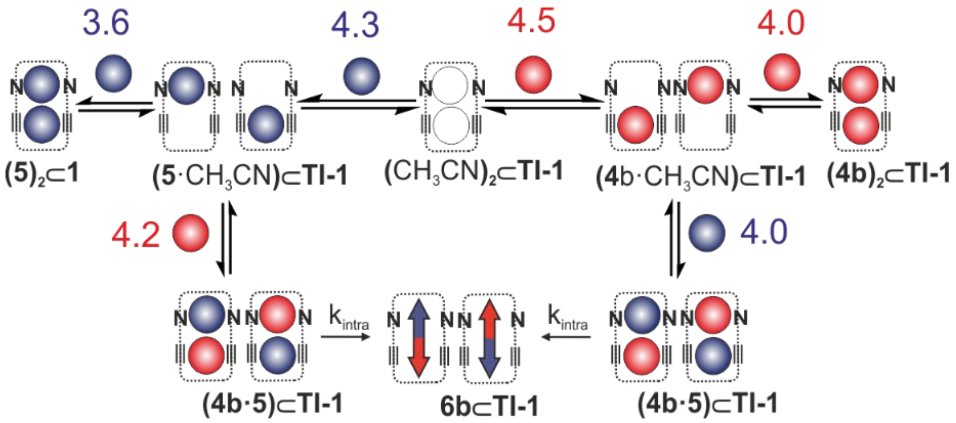

Figure S 46. Theoretical kinetic model used for the non-linear analysis of the experimental data from the mediation of the reaction between **4b** and **5.** The thermodynamic equilibrium constants for each binding process are included. Red spheres correspond to azido-substrate **4b** and blue spheres to ethynyl substrate **5.** Regioisomeric complexes were treated as a single species and were not distinguished as separate entities in the model.

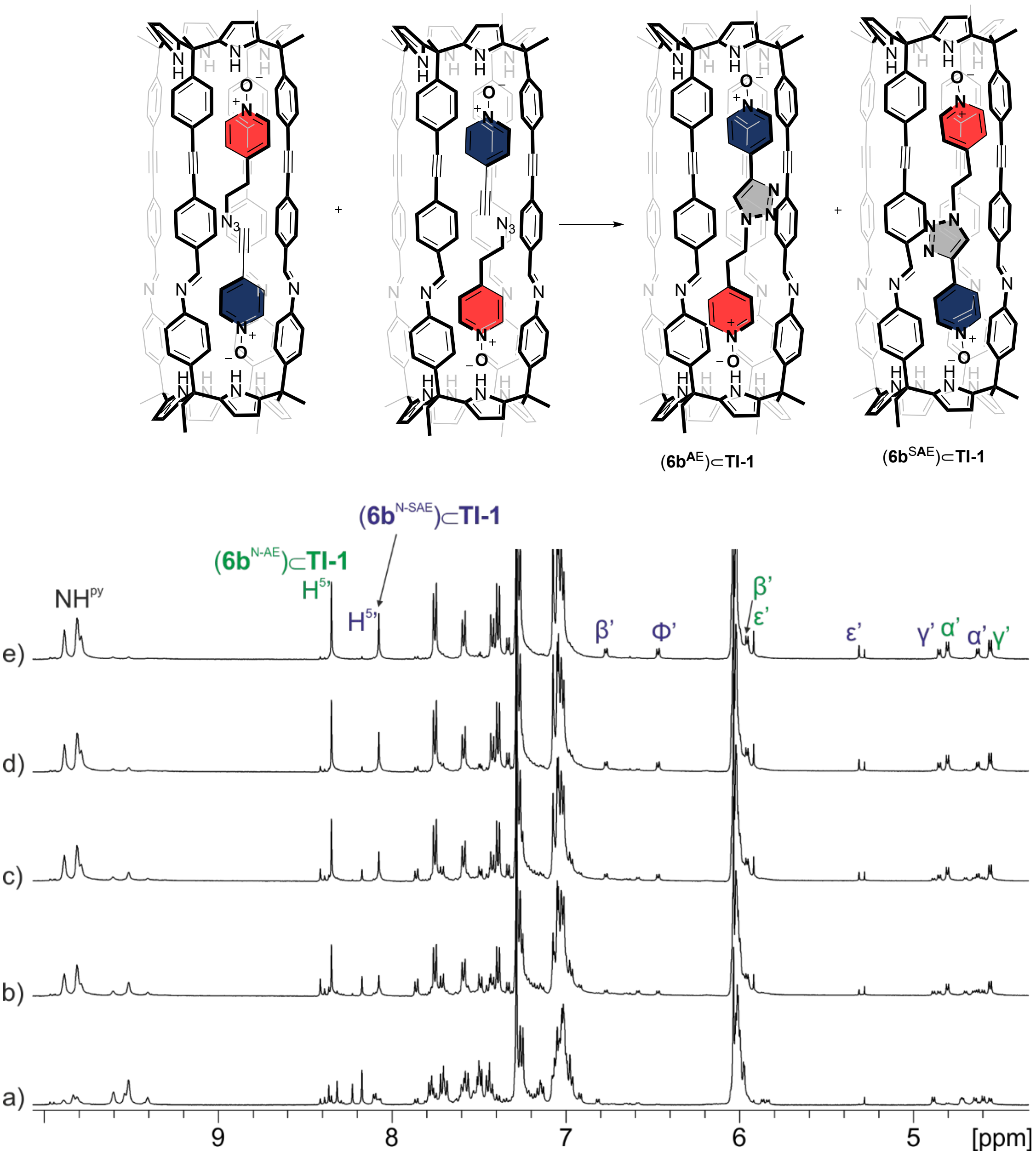

Figure S 47. Top) Dipolar cycloaddition reaction between **4b** and **5** included in **TI-1** to yield **6b⊂TI-1** complex. Bottom) Selected region of the ^{1}H NMR spectra corresponding to the monitoring of the formation of complex **6b⊂TI-1** from an equimolar 2 mM mixture of **TI-1**, **4b**, and **5** in $CDCl_3$:CD_3CN 9:1 solvent mixture. a) 0 min, b) 30 min, c) 1 h, d) 2 h, and e) 3 h. Primed numbers correspond to 1:1 complexes, and blue and green colors discriminate the protons from the two possible regioisomers of the 1:1 complexes, (CD_3CN•**6b**^{SAE})⊂**TI-1** (blue), and (CD_3CN•**6b**^{AE})⊂**TI-1** (green).

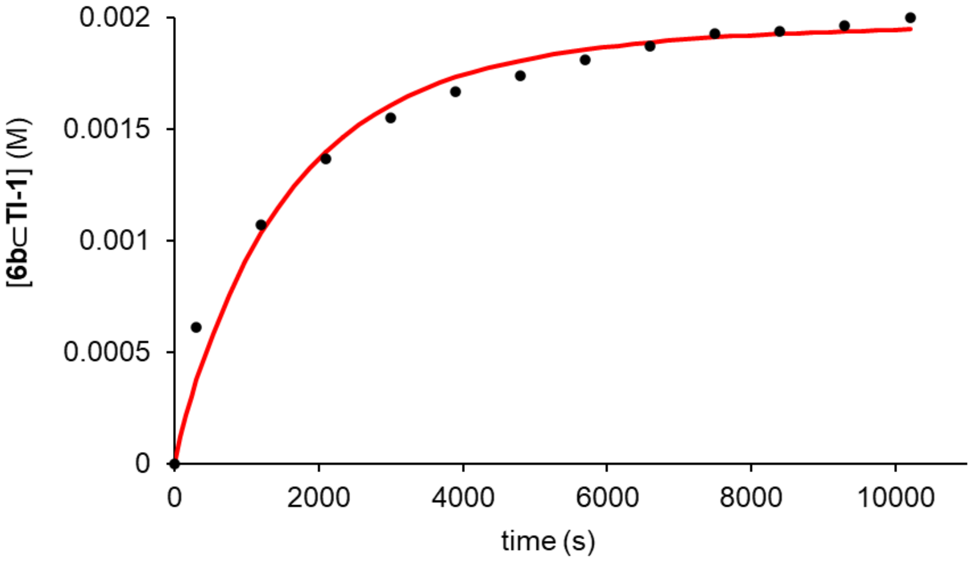

Figure S 48. Change in the concentration of the two regioisomeric complexes **6b⊂TI-1** (black dots) with time, starting from a 1:1:1 2 mM mixture of **TI-1**, **4b** and **5**. Solid red line represents the fit of the experimental data to the theoretical kinetic model using the parameters estimation module of COPASI software version 4.25. The k_{on}/k_{off} ratio of all the binding equilibria were manually fixed based on the estimated k_{off} and the determined thermodynamic binding constants. k_{intra} was the only variable parameter used for the fit k_{intra} = 3.76 (± 0.3) × 10^{-3} s^{-1}.

Table S 1. Kinetic parameters (k_{intra} and EM_{kin}) for the 1,3-dipolar cycloadditions of **5** with **4a** and **4b** occurring within **TI-1,** together with the association constants of the corresponding Michaelis complexes (K_a). The association constants (K_a) and thermodynamic effective molarities (EM_{thermo}) of the triazole complexes **6⊂TI-1** are also included.

Complex	K_a	k_{intra}	EM_{kin}	EM_{thermo}
**(4a•5)⊂TI-1**	2×10^8 M^{-2} [a]	$2.0 \pm 0.1 \times 10^{-6}$ [c] s^{-1}	36 ± 4 [d] M	n.a.
**(4b•5)⊂TI-1**	3×10^8 M^{-2} [a]	$3.8 \pm 0.3 \times 10^{-3}$ [c] s^{-1}	1.1 ± 0.7 x 10^5 [d] M	n.a
**6a⊂TI-1**	3×10^5 M^{-1} [b]	n.a.	n.a	3×10^{-3} [e] M
**6b⊂TI-1**	> 10^7 M^{-1} [b]	n.a	n.a.	> 0.1 [e] M

[a] Determined by iterative manual refinement of the theoretical speciation profiles produced using HySS2009 software to the experimentally measured concentration ratio of the different species detected in the ^{1}H NMR spectra of the titrations of **TI-1**. We used a binding model that accounts for all possible binding equilibria between substrates **4** and **5** and **TI-1** (1:1 and 2:1 homo- and hetero-complexes). A single geometric isomer was considered for the 1:1 and 2:1 heterocomplexes; [b] Determined from competitive ^{1}H NMR experiments using monotopic guest **5** as reference; [c] Determined from the fit of the full-time-course ^{1}H NMR kinetic data of the cycloaddition reaction between **5** and **4a** or **4b** in the presence of equimolar amounts of **TI-1** using the kinetic model described in Figure 5 and COPASI software; [d] $EM_{kin} = k_{intra}/k_{bulk}$. k_{bulk} was taken from reference 22.[e] EM_{thermo}= K(**6⊂TI-1**)/K(**5₂⊂TI-1**). K(**5₂⊂TI-1**) = 8.1×10^7 M^{-2}. n.a. not applicable

6. DFT calculations

DFT calculations

A TS geometry connecting one of the isomers of the Michaelis complexes to each of the products, yielding **6a^{AE}⊂TI-1** and **6b^{AE}⊂TI-1**, was located and optimized at the RI[1,2,3]-BP86[1]-def-SV(P)[4,5] level of theory as implemented in Turbomole 7.8.[6] Intrinsic reaction coordinate (IRC) calculations confirmed TS connectivity and furnished the optimized product inclusion complexes **6a^{AE}⊂TI-1** and **6b^{AE}⊂TI-1,** as well as the corresponding Michaelis complexes (**4^{AE}·5^{SAE}⊂TI-1**), which were further optimized at the same level of theory.

All dataset collection of computational results of this manuscript is available in the ioChem-BD repository[7] and can be accessed through this link https://iochem-bd.iciq.es/browse/review-collection/100/113869/48743406767898f5dad5e243.

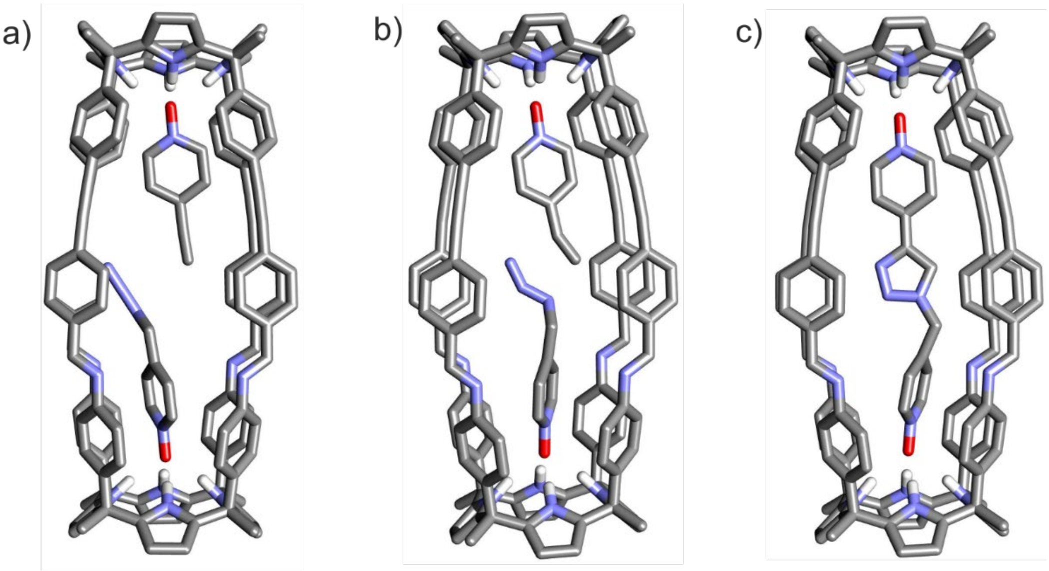

Figure S 49. DFT (RI-BP86 def-SV(P)) energy minimized structures of a) **(4a^{AE}•5^{SAE})$\subset$TI-1** ternary complex, b) **6a$^{AE\text{-}TS}$$\subset$TI-1** transition state geometry for the corresponding cycloaddition reaction calculated at the same theoretical level, and c) **6a^{AE}$\subset$TI-1** product complex. Supplementary Movie 1 shows the animated imaginary frequency of the **6a$^{AE\text{-}TS}$$\subset$TI-1** transition state.

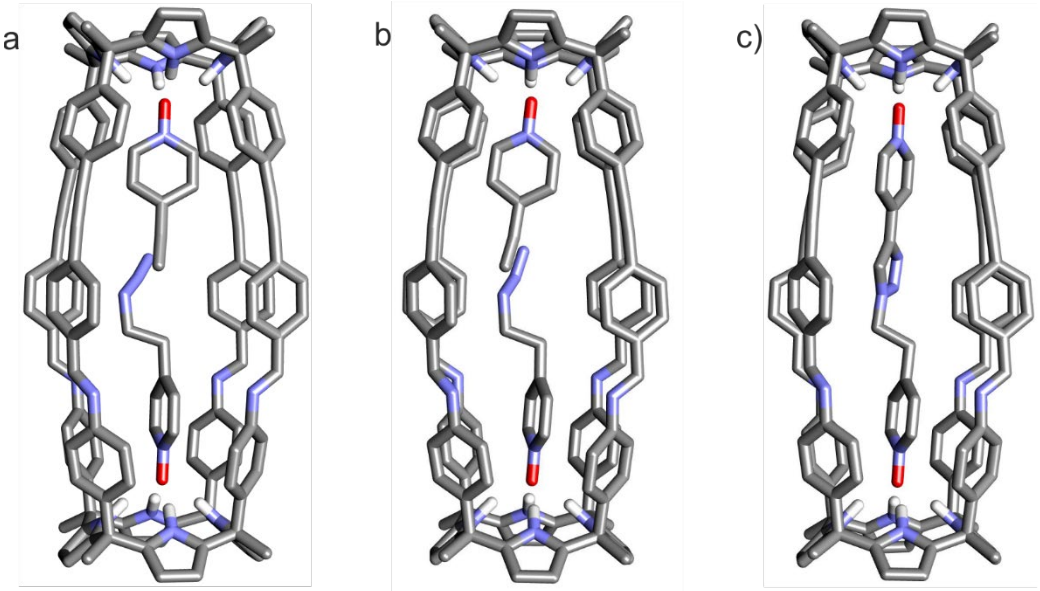

Figure S 50. DFT (RI-BP86 def-SV(P)) energy minimized structures of a) **(4b^{AE}•5^{SAE})$\subset$TI-1** ternary complex, b) **6b$^{AE\text{-}TS}$$\subset$TI-1** transition state geometry for the corresponding cycloaddition reaction calculated at the same theoretical level, and c) **6b^{AE}$\subset$TI-1** product complex. Supplementary Movie 1 shows the animated imaginary frequency of the **6b$^{AE\text{-}TS}$$\subset$TI-1** transition state.

7. X-ray crystal structure

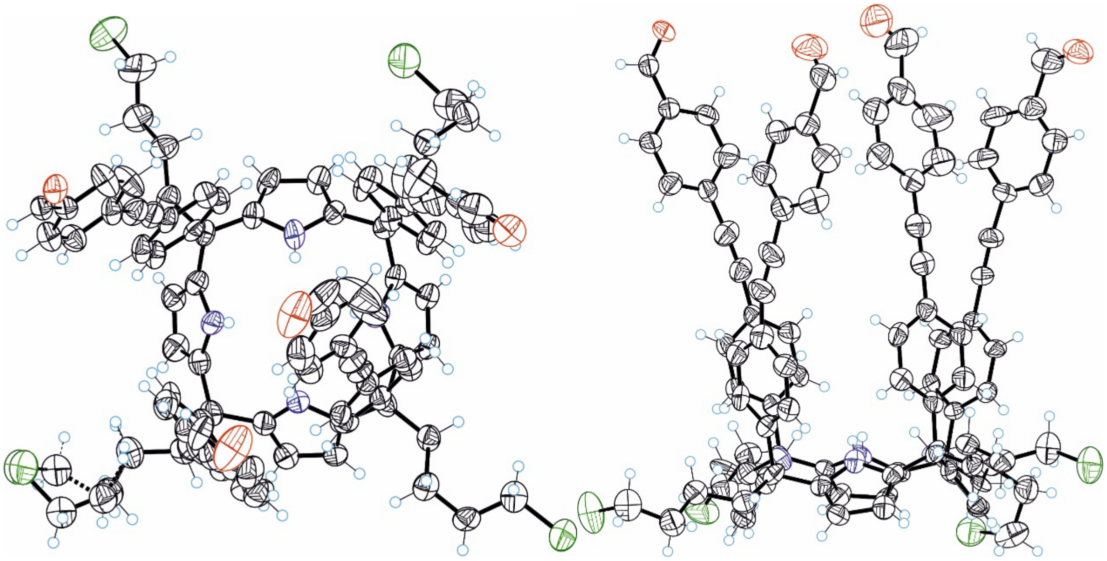

Figure S 51. Top and side views of the X-ray structure of tetra-formyl meso-tetra-4-chlorobutyl-tetra-p-(ethynyl-benzaldehyde)phenyl calix[4]pyrrole, SAE-C[4]P **2**. The structures are shown as ORTEP representations with thermal ellipsoids set at 50% probability for the non-hydrogen atoms. Hydrogen atoms are depicted as fixed-size spheres with a radius of 0.15 Å.

8. References

1 J. P. Perdew, Density-functional approximation for the correlation energy of the inhomogeneous electron gas, *Physical Review B*, 1986, **33**, 8822-8824.

2 K. Eichkorn, F. Weigend, O. Treutler and R. Ahlrichs, Auxiliary basis sets for main row atoms and transition metals and their use to approximate Coulomb potentials, *Theor. Chem. Acc.*, 1997, **97**, 119-124.

3 M. Sierka, A. Hogekamp and R. Ahlrichs, Fast evaluation of the Coulomb potential for electron densities using multipole accelerated resolution of identity approximation, *J. Chem. Phys.*, 2003, **118**, 9136-9148.

4 D. Rappoport and F. Furche, Property-optimized Gaussian basis sets for molecular response calculations, *J. Chem. Phys.*, 2010, **133**, 134105-134101-134105-134111.

5 A. Schäfer, H. Horn and R. Ahlrichs, Fully optimized contracted Gaussian basis sets for atoms Li to Kr, *J. Chem. Phys.*, 1992, **97**, 2571-2577.

6 TURBOMOLE Program Package For Electronic Structure 817 Calculations. https://www.turbomole.com.

[7] M. Álvarez-Moreno, C. de Graaf, N. López, F. Maseras, J. M. Poblet and C. Bo, *J. Chem. Inf. Model.*, 2015, **55**, 95-103.
