# Supplementary material for: Balancing acceleration and turnover in [1 + 1] tetra-imine bis-calix[4]pyrrole reactor for Huisgen cycloadditions
Source: Nat Commun. 2026 Apr 27;17:6525. doi: 10.1038/s41467-026-72315-w (PMC13377019; doi:10.1038/s41467-026-72315-w)
Supplement: Supplementary file 2 — Description of Additional Supplementary Files [file 41467_2026_72315_MOESM2_ESM.pdf]

Description of Additional Supplementary Files

File Name: Supplementary Movie 1
Description: Animated imaginary frequency of the $6a^{AE\text{-}TS}\subset$TI-1 transition state.

File Name: Supplementary Movie 2
Description: Animated imaginary frequency of the $6b^{AE\text{-}TS}\subset$TI-1 transition state.
