# Supplementary material for: Balancing acceleration and turnover in [1 + 1] tetra-imine bis-calix[4]pyrrole reactor for Huisgen cycloadditions
Source: Nat Commun. 2026 Apr 27;17:6525. doi: 10.1038/s41467-026-72315-w (PMC13377019; doi:10.1038/s41467-026-72315-w)
Supplement: Supplementary file 5 — Transparent Peer Review file [file 41467_2026_72315_MOESM5_ESM.pdf]

nature portfolio

Peer Review File

Balancing acceleration and turnover in [1 + 1] tetra-imine bis-calix[4]pyrrole reactor for Huisgen cycloadditions

Corresponding Author: Professor Pablo Ballester

This file contains all reviewer reports in order by version, followed by all author rebuttals in order by version.

Version 0:

Reviewer comments:

Reviewer #1

(Remarks to the Author)
The manuscript by Ballester et al titled "Balancing acceleration and turnover in [1 + 1] tetra-imine biscalix[4]pyrrole reactor for Huisgen cycloadditions" reports a tetra-imine-functionalized bis-calix[4]pyrrole cage exhibiting two chemically non-equivalent cavities that functions as a reactor vessel for azide-alkyne Huisgen cycloadditions. Para-substituted pyridine-N-oxides as guests form 1:1 and 1:2 complexes with the cage, showing that a confinement-driven acceleration and catalytic turnover in bis-calix[4]pyrrole cage proceed smoothly. The authors compere the tetra-imine to the previosly reported octa-imine cage and found that the ca. 0.6 Å elongation of the cavity in the tetra-imine cage modulates the balance between reaction acceleration and turnover, preserving 1,4-regioselectivity.

Crystallographically the predominantly organic (heaviest atom present = chlorine) supramolecular structure is remarkably well modelled despite the usual issues with supramolecular crystallography (weak diffraction/poor resolution, etc). The R-factors indicate the data is exceptionally weak with R1(Io > 2σ[Io])/R1(all data) = 8.2/18.1% and Rint = 12.8%, which though elevated, are completely within expectations for this type of structure. The authors have been careful in the main text not to extrapolate information from the CIF beyond what its quality would support. The authors properly addressed deficiencies in the CIF via embedded author responses, even clarifying/estimating the contents of the omitted electron density (done via Olex's Bypass functionality) as $C_6H_4Cl_2$, which fit well (292 versus 296 electrons given the data quality). There are no significant residual peaks of electron density warranting further comment. Examination of the ORTEP plot shows the authors treatment of the thermal parameters to be reasonable, despite a few outliers (e.g., C37). The only criticism would be the application of a 'global' RIGU restraint, which in itself is understandable for a supramolecular structure, but not when it is overlapped with other subsequently tighter RIGU restraints at non-default values, ISOR restraints (neither of which seem to be helping the model much), and EADP constraints. The specifically restrained atoms should be removed from the global RIGU restraint, so as not to overlap conflicting treatments of the thermal ellipsoids of the atoms in question. However, this criticism should be interpreted as 'best practice' and does not prevent the publication of the structure in its current form.

As a summary the science is excellently conducted, the results are interesting and, indeed, highlight the importance of subtle spatial modulations on the chemical structure of artificial capsular receptors. Based on the above publication of this fine piece of work in Nature Communications is recommended.

Reviewer #2

(Remarks to the Author)
Ballester and co-workers report on a very detailed study on the bonding and reactive properties of alkyne and azido derivatives in the confined space of a bis-calix[4]pyrrole cage. The design of the cage was slightly adapted compared to their recent report (JACS Au, 2025, 902); a tetra-imine linker was replaced by a tetra-alkynyl one which leads to a slight geometric difference (the two openings located on each side of the cage has a difference of only ~ 1 Å) which translated into strong differences in terms of binding and catalytic properties between the symmetrical (O1-1) and lower symmetrical (T1-1) cages. The study is conducted in a very professional manner with detailed and precise presentation of the experimental data and of their quantitative exploitation, even though some technicities somewhat complicate the reading. In my point of view, the fact that the acceleration/inhibition dichotomic issue displayed by (most of) the conventional enzymatic-like cages have been alleviated by a very subtle but powerful design of the cage makes the report very original and will contribute significantly to the design of future catalytic cages. A point that is somewhat surprising is that the azido substrate with the longer linker (4b),

which is thus expected to be more flexible, leads to stronger binding in the cage and then to product inhibition. This counterintuitive effect is well described by the kinetic and thermodynamic data and rationalized by a somewhat better fitting of the substrate and the product in the cavity, whose structures have been computed by DFT. I suggest the following points to be addressed prior to publication in Nature Commun:

(1) Problem of inhibition in cage-type catalysis: The introduction appears too narrow in its explication and scope to provide a global overview of the dichotomic issue of acceleration and inhibition. The authors make (sometimes implicitly) a distinction between systems for which acceleration is promoted by enthalpic or entropic effects. Following this classification, the authors rank a large part of the state-of-the-art systems as accelerated by enthalpic factors whilst their system is suggested to obey the original enzymatic-type of activation governed by entropy. This is not clear to my point of view on which scientific basis the authors make this distinction. Even in the present report, the contribution of enthalpy and entropy to the acceleration has not been made. The substrates that are strongly hydrogen-bonded (a typical enthalpy contribution) leads to the higher acceleration. The authors must support their claim that the observed acceleration is promoted by (reduction) of the entropic cost in the bimolecular reaction under study. Even though a reference is provided, it is not clear how the authors extract the dS from EMkin in the sentence “approaching enzyme-like entropic contributions (i.e. ~ - 35 e.u.).” In the same vein, the following sentence must be rephrased/clarified “The trend inverts for the reacting pair involving the longer azide 4b and 5 (Table 2): TI-1 delivers an enzyme-like entropic enhancement (EMkin > 10^4 M), whereas it is far less accelerating than OI-1 (EMkin = 103 M).”
(2) Strength of the hydrogen-bonding interactions: The better fitting of 4b and 6b relatively to 4a and 6a, resp., is attributed to stronger hydrogen bonding interactions between the pyrrole N-H and the pyridine N-oxide moieties. The authors must provide additional data to support these claims. The CIS is said to be smaller for 6a relatively to 6b but this is actually the opposite which is shown in Fig. 6c and 6d.
(3) Kinetic and thermodynamic parameters: There is a plethora of kinetic and thermodynamic parameters mentioned in the manuscript and there is only one Figure (Fig.5a) which helps to visualize the nature of these parameters. I strongly suggest the authors to list and define all these parameters in order to be better understood by the readers. Additional information must also be provided on how these parameters are extracted from the different fits.

Reviewer #3

(Remarks to the Author)
This manuscript by Ballester and coworkers describes the design and quantitative self-assembly of a [1+1] tetra-imine biscalix[4]pyrrole cage (TI-1) that functions as a molecular reactor for Huisgen azide–alkyne cycloadditions. The study presents an elegant supramolecular system in which subtle structural modulation of the cage cavity enables a systematic examination of the balance between catalytic acceleration and product inhibition. The work is carefully executed and combines synthesis, supramolecular binding studies, kinetic analysis, and DFT calculations to provide a coherent mechanistic picture of confined reactivity. Particularly noteworthy is the quantitative kinetic modeling that extracts intra-vessel rate constants while accounting for supramolecular equilibria, as well as the demonstration that sub-Å cavity elongation can significantly alter the interplay between transition-state stabilization and product release. The ability to tune catalytic acceleration versus turnover through minimal geometric modification represents an important conceptual contribution to the field of supramolecular catalysis and molecular reactor design. Overall, the manuscript is well written, the experimental evidence is convincing, and the conclusions are well supported. As such, I am pleased to recommend this work for publication in Nature Communications with minor revisions.

Minor revision:
1. Please remove the period following “Huisgen cycloadditions” in the title
2. The border artifacts in Figures 6a and 6b should be removed to improve the clarity of the figures.

Reviewer #4

(Remarks to the Author)
I co-reviewed this manuscript with one of the reviewers who provided the listed reports. This is part of the Nature Communications initiative to facilitate training in peer review and to provide appropriate recognition for Early Career Researchers who co-review manuscripts.

Reviewer #5

(Remarks to the Author)
This manuscript by Ballester and co-workers describes the synthesis of a new unsymmetrical calix pyrrole receptor alongside a detail physical-organic analysis of it’s host-guest properties and application as a catalyst for Huisgen cycloaddition. This is a really high-quality, detailed investigation that probes the effects that substrate structure has on the propensity of the receptor to accelerate chemical reactions based on very subtly different structures (c.f., enzymes). The key results the authors present are significant for the field of non-covalent catalysis as a whole; notably the interplay between acceleration and catalytic turnover, and how the reaction can be tuned to give both high acceleration or effective turnover. It is also extremely notable that the acceleration reported by the authors for one of the substrates is one of the highest reported for a system that relies almost solely on the entropic effects associated with dual binding. Moreover, it starts to approach the theoretical limit that was proposed by Jenks several decades ago. There are also lots of other interesting aspects to this paper, notably the use of a low symmetry receptor, which has been rarely reported in this area. I think this has significant implications for other catalytic approaches (e.g., based on electrostatics) that will be subsequently exploited. I thought the more subtle aspects of the paper, for example how kinetic binding was influenced by which end of the capsule the substrate

enters the cavity from, was also really interesting.

The quality of this paper, from the skilful, non-trivial analysis of the high-quality data through to the manuscript figures, is really excellent. The authors also cite a good range of sources, both on their own and similar systems as well as other examples of non-covalent catalysts (e.g., metallo-organic cages). My overall opinion is that this is a really excellent paper with lots of thought-provoking science that easily merits publication.

Version 1:

Reviewer comments:

Reviewer #1

(Remarks to the Author)
The authors have revised the manuscript accordingly and satisfactorily, thus this reviewer support the publication of this fine work in NAture Comunications in its current, revised form.

Reviewer #2

(Remarks to the Author)
The revised report by Ballester and co-workers on the tuning of the acceleration/inhibition of a catalytic Huisgen reaction in the confined space of a bis-calix[4]pyrrole cage results in a more accessible manuscript for the general audience of Nature Commun. This referee particularly appreciates the better positioning of the present work relatively to other systems of the literature notably regarding the enthalpic/entropic nature of the observed accelerations and the improved overall reading of the paper. Please precise the chemical nature of the R, R1 and R2 groups in Scheme 1 prior to publication (this can be added in proof, no need for further referring).

Reviewer #4

(Remarks to the Author)
I co-reviewed this manuscript with one of the reviewers who provided the listed reports. This is part of the Nature Communications initiative to facilitate training in peer review and to provide appropriate recognition for Early Career Researchers who co-review manuscripts.

Open Access This Peer Review File is licensed under a Creative Commons Attribution 4.0 International License, which permits use, sharing, adaptation, distribution and reproduction in any medium or format, as long as you give appropriate credit to the original author(s) and the source, provide a link to the Creative Commons license, and indicate if changes were made.
In cases where reviewers are anonymous, credit should be given to 'Anonymous Referee' and the source.
The images or other third party material in this Peer Review File are included in the article’s Creative Commons license, unless indicated otherwise in a credit line to the material. If material is not included in the article’s Creative Commons

license and your intended use is not permitted by statutory regulation or exceeds the permitted use, you will need to obtain permission directly from the copyright holder.
To view a copy of this license, visit https://creativecommons.org/licenses/by/4.0/

Reviewer #1

1. Examination of the ORTEP plot shows the authors treatment of the thermal parameters to be reasonable, despite a few outliers (e.g., C37). The only criticism would be the application of a ‘global’ RIGU restraint, which in itself is understandable for a supramolecular structure, but not when it is overlapped with other subsequently tighter RIGU restraints at non-default values, ISOR restraints (neither of which seem to be helping the model much), and EADP constraints. The specifically restrained atoms should be removed from the global RIGU restraint, so as not to overlap conflicting treatments of the thermal ellipsoids of the atoms in question. However, this criticism should be interpreted as ‘best practice’ and does not prevent the publication of the structure in its current form.

Answer) We thank the reviewer for the positive evaluation of our work and for the careful assessment of the crystal data. We agree with the comment regarding best practices in refinement. The extensive use of restraints and constraints in our refinement arises from the limited resolution of the diffraction data, which resulted in an unfavorable data-to-parameter ratio. Under these conditions, we employed these tools to maintain a stable refinement and ensure a chemically reasonable structural model.

We have clarified the refinement strategy more explicitly in the Supporting Information document. We also fully acknowledge the reviewer’s recommendation regarding the avoidance of overlapping restraint schemes (global and atom-specific RIGU restraints), and we will follow this best-practice approach in future refinements.

“*Due to the limited resolution of the diffraction data and the resulting low data-to-parameter ratio, additional restraints and constraints were applied to ensure a stable refinement and chemically reasonable structural model. Specifically, a global RIGU restraint was employed, supplemented by atom-specific RIGU and ISOR restraints where necessary. EADP constraints were applied to selected atom pairs in close proximity or disordered environments to further stabilize their displacement parameters.*”

Reviewer #2

1.1. Problem of inhibition in cage-type catalysis: The introduction appears too narrow in its explication and scope to provide a global overview of the dichotomic issue of acceleration and inhibition.

Answer) We completely agree with the reviewer's comment. Our intention in the introduction was not to provide a comprehensive overview of all examples of acceleration and inhibition cage-type catalysis, but rather to focus on those systems most relevant to the present work. For this reason, we focused on examples of catalytic turnover for bimolecular reactions where both substrates are simultaneously co-bound within a confined cavity to form a well-defined ternary complex. In such co-encapsulation scenarios, product inhibition is frequently observed because the ditopic product often binds more strongly to the host than the individual monotopic substrates.

To avoid excluding other important examples of turnover in supramolecular systems, we briefly mentioned examples that operate through different approaches and also achieve catalytic turnover, for example, systems in which only one substrate is encapsulated, or those in which the reaction occurs outside the cavity but is influenced by the host (refs. 13–16 in the original manuscript). Moreover, our aim was not to classify these examples in terms of enthalpic or entropic control (see point 1.2 raised by reviewer #2), but simply to acknowledge the diversity of approaches that can lead to catalytic turnover.

To address the reviewer's concern, we have revised the introduction to clarify the scope and make more explicit that our selection of examples was motivated by the mechanistic proximity to our work.

Original text: "*Other examples of DA reactions demonstrating molecular vessel turnover do not involve a well-defined ternary Michaelis complex, and the catalytic effect is mainly ascribed to enthalpic factors of TS stabilization.*[13,14,15,16]"

Revised version: "*Other reported examples of DA reactions showing catalytic turnover rely on fundamentally different mechanisms not involving the formation of a ternary Michaelis complex (do not co-confine both substrates). In many of these systems, only one substrate is included within the molecular cavity, or the reaction proceeds outside the cavity, and the resulting catalytic effect is mainly ascribed to substrate polarization and electronic stabilization of the TS, rather than to bimolecular pre-organization within a confined*

environment. Because these approaches operate through a distinct mechanism, they are not directly relevant for comparison with the present investigations."

1.2. The authors make (sometimes implicitly) a distinction between systems for which acceleration is promoted by enthalpic or entropic effects. Following this classification, the authors rank a large part of the state-of-the-art systems as accelerated by enthalpic factors whilst their system is suggested to obey the original enzymatic-type of activation governed by entropy.

This is not clear to me; from my point of view, the authors do not provide a scientific basis for this distinction. Even in the present report, the contributions of enthalpy and entropy to the acceleration have not been addressed.

Answer) We thank the reviewer for this important comment. We agree that the previous wording could be interpreted as implying a distinction between molecular vessels for which catalytic acceleration of bimolecular reaction was mainly derived from the enthalpic or entropic term. Our intention was instead to distinguish between two possible extreme scenarios commonly involved in supramolecular catalysis: (i) acceleration arising from substrate polarization/electronic TS stabilization, that is mainly enthalpic in origin and (ii) acceleration arising from reactants' organization within the container's cavity, impacting on the entropic counterpart, i.e., reduction of translational and rotational entropy costs associated with a bimolecular reaction in bulk solution. Andrew's recent perspective makes this distinction precisely in modern terms: organization (control of position/orientation; often discussed as confinement/effective molarity) versus polarization/electrostatic/H-bond/acid-base TS stabilization. We have used these terms in the revised version, avoiding an overgeneralized "enthalpic vs entropic classification". Accordingly, we have also cited Andrew's perspective.

1.3 The substrates that are strongly hydrogen-bonded (a typical enthalpy contribution) leads to the higher acceleration. The authors must support their claim that the observed acceleration is promoted by (reduction) of the entropic cost in the bimolecular reaction under study. Even though a reference is provided, it is not clear how the authors extract the dS from EM_{kin} in the sentence "approaching enzyme-like entropic contributions (i.e. ~ - 35 e.u.).

Answer) Strong binding interactions in the ternary complex are not, by themselves, evidence that the acceleration is "enthalpic." The key is to separate the stabilization of the ternary complex from the physical origin of the reaction-rate acceleration within the cage. As the reviewer points out, host-guest binding in organic solvents is often driven by large enthalpic terms arising from favorable electrostatic interactions (e.g., hydrogen bonds, CH-π, and π-π interactions). The favorable enthalpic terms are offset at least by an entropic penalty of bringing the molecules together. In short, strong binding is required to populate the reactive ternary complex to an appreciable extent under dilute (mM) conditions. This, in turn, demands sufficiently favorable host–guest intermolecular interactions, which in organic solvents are typically dominated by enthalpically favorable electrostatic contributions.

These binding thermodynamics are distinct from the physical origin of the catalytic rate enhancement once the substrates are co-localized inside the cage. The reaction rate enhancement for the confined reaction arises mainly because the cage converts a dilute, bimolecular process occurring in bulk solution into an intramolecular-complex, unimolecular-like reaction, thereby greatly reducing the entropy penalty of binding the two substrates together (i.e. $\Delta S^{\ddagger}_{intra}$ is less negative than $\Delta S^{\ddagger}_{bulk}$).

We consider our results consistent with an organization or effective-molarity-driven origin of the acceleration. In fact, in a previous related study from our group (Chemical Science 2025, 16, 14534), we demonstrated, through Eyring analysis, that the observed rate acceleration was predominantly driven by a reduction in the entropic cost of the confined bimolecular process, consistent with the cage functioning as an "entropic trap." We have clarified this point in the revised manuscript and explicitly referenced these results. We also added a note in the revised manuscript that explains how the $\Delta\Delta S^{\ddagger}$ value between the reaction confined in the cage and in the bulk is extracted from the experimentally determined EM_{kin}. We use the relationship $EM_{kin} = e^{-\Delta\Delta S^{\ddagger}/R}$. In doing so, we assign the acceleration of the confined reaction exclusively to entropic factors *(i.e. $\Delta\Delta H^{\ddagger} \approx 0$)*, in agreement with organization effects being the primary responsible factor. Therefore, a $\Delta\Delta S^{\ddagger} = \Delta S^{\ddagger}_{bulk} - \Delta S^{\ddagger}_{intra} = -R \ln EM_{kin} = -35$ e.u. corresponds to an $EM_{kin} \sim 10^{7}$ M.

Below, we included the revised parts that can be found in the revised manuscript to clarify these issues:

Original text: "*For bimolecular reactions, the co-binding of the substrates in the so-called Michaelis complex converts an intermolecular process into an effectively intramolecular*

one. Collectively, these are entropic effects impacting the catalytic properties of the cages."

Revised version: "*For bimolecular reactions, the co-binding of the substrates in the so-called Michaelis complex converts an intermolecular process into an effectively intramolecular one. Collectively, these are organization effects dominating the catalytic properties of the cages. Polarization/electronic stabilization of the TS and solvation/desolvation effects may also contribute to, or even be the main reason for catalytic activity in other systems.*"

Original text: *"The resulting acceleration expressed as EM_{kin}, was estimated to be ~10^5 M, approaching the predicted theoretical maximum (~10^6 M).*"

Revised version: "*The resulting acceleration expressed as EM_{kin}, was estimated to be ~10^5 M, under the assumption that it arises exclusively from reactants' organization within the host cavity. This value is close to the predicted theoretical maximum (~10^6 M).*"

Original text: *"This result agrees with the initial-rate estimate (EM_{kin} ≈ 45 M). Global fitting of the reaction **4b** + **5** inside **TI-1** yields $k_{\mathbf{6b}\text{-}intra}$= 3.8 ± 0.3 × 10^{-3} s^{-1} (Figure 5c), corresponding to EM_{kin} = 6.8 ± 0.7 x 10^4 M (ΔS ≈ −22 e.u.), approaching enzyme-like entropic contributions (i.e. ~ - 35 e.u.).*"

Revised version: *"This result agrees with the initial-rate estimate (EM_{kin} ≈ 45 M). Global fitting of the reaction **4b** + **5** inside **TI-1** yields $k_{\mathbf{6b}\text{-}intra}$= 3.8 ± 0.3 × 10^{-3} s^{-1} (Figure 5c), corresponding to EM_{kin} = 1.1 ± 0.7 x 10^5 M ($\Delta\Delta S^{\ddagger} = \Delta S^{\ddagger}_{bulk} - \Delta S^{\ddagger}_{intra} = -R \ln(EM_{kin})$ ≈ −*23 e.u.*), approaching enzyme-like entropic contributions (i.e. ~ - 35 e.u.)."*

Additional note in the revised manuscript: "This approximation assumes that the acceleration arises exclusively from entropic effects (i.e. $\Delta\Delta H^{\ddagger} \approx 0$)."

Original text: *"In the octa-imine cage **OI-1**, two converging polar binding sites strongly bind para-substituted pyridine-N-oxide and 2-pyridinone derivatives, suitable for Huisgen cycloadditions, and organize their reactive ends in face-to-face arrangements resembling, in the 1:1:1 "Michaelis" complex, the bulk transition state (TS) geometry of the 1,4-triazole, accounting for both acceleration (primarily entropic) and regioselectivity.*"

Revised version: "*In the octa-imine cage **OI-1**, two converging polar binding sites strongly bind para-substituted pyridine-N-oxide and 2-pyridinone derivatives, suitable for Huisgen cycloadditions, and organize their reactive ends in face-to-face arrangements resembling,*

in the 1:1:1 "Michaelis" complex, the bulk transition state (TS) geometry of the 1,4-triazole, accounting for both acceleration (primarily entropic) and regioselectivity. We showed through Eyring analysis that the observed rate acceleration arose mainly from a reduction in the entropic cost of the bimolecular process, consistent with the cage acting as an "entropic trap"."

1.4. In the same vein, the following sentence must be rephrased/clarified "The trend inverts for the reacting pair involving the longer azide 4b and 5 (Table 2): TI-1 delivers an enzyme-like entropic enhancement (EM_{kin} > 10^4 M), whereas it is far less accelerating than OI-1 (EM_{kin} = 10^3 M)."

Answer) Following the reviewer's recommendation, we rephrased the specified sentence in the revised version of the manuscript to clarify the comparison between the two systems and now it reads as follows:

Original text: "*The trend inverts for the reacting pair involving the longer azide 4b and 5 (Table 2): TI-1 delivers an enzyme-like entropic enhancement (EM_{kin} > 10^4 M), whereas it is far less accelerating than OI-1 (EM_{kin} = 10^3 M).*"

Revised version: "*This trend is reversed for the reacting pair involving the longer azide **4b** and **5** (Table 2). The **TI-1** cage provides an enzyme-like entropic enhancement (EM_{kin} > 10^4 M), whereas the **OI-1** cage shows a significantly smaller effect (EM_{kin} = 10^3 M).*"

2. Strength of the hydrogen-bonding interactions: The better fitting of 4b and 6b relatively to 4a and 6a, resp., is attributed to stronger hydrogen bonding interactions between the pyrrole N-H and the pyridine N-oxide moieties. The authors must provide additional data to support these claims. The CIS is said to be smaller for 6a relatively to 6b, but this is actually the opposite, which is shown in Fig. 6c and 6d.

   Answer) We thank the reviewer for spotting that Figures 6c and 6d contradicted the discussion in the manuscript. Upon revision, we identified an error in the labelling of the ^{1}H NMR spectra in Figure 6: the spectrum originally labelled as (c) actually corresponds to the isomers of **6b⊂TI-1**, while the one labelled as (d) corresponds to the isomers of **6a⊂TI-1** complex. We have corrected this mistake in the revised version of the manuscript. With this correction, the trends discussed in the manuscript are consistent with stronger hydrogen-bonding interactions between the pyrrole N-H of **TI-1** cage and the pyridine *N*-oxide moieties in **6b** compared to **6a**.

3. Kinetic and thermodynamic parameters: There is a plethora of kinetic and thermodynamic parameters mentioned in the manuscript and there is only one Figure (Fig.5a) which helps to visualize the nature of these parameters. I strongly suggest the authors to list and define all these parameters in order to be better understood by the readers. Additional information must also be provided on how these parameters are extracted from the different fits.

Answer) We thank the reviewer for this suggestion. We have added a new table summarizing all the kinetic and thermodynamic parameters used in the manuscript in the revised supplementary information. We have clearly indicated how each parameter was obtained.

Table S1. Kinetic parameters (k_{intra} and EM_{kin}) for the 1,3-dipolar cycloadditions of **5** with **4a** and **4b** occurring within **TI-1,** together with the association constants of the corresponding Michaelis complexes (K_a). The association constants (K_a) and thermodynamic effective molarities (EM_{thermo}) of the triazole complexes **6⊂TI-1** are also included.

Complex	K_a	k_{intra}	EM_{kin}	EM_{thermo}
**(4a•5)⊂TI-1**	2×10^{8} M^{-2} [a]	$2.0 \pm 0.1 \times 10^{-6}$ [c] s^{-1}	36 ± 4 [d] M	n.a.
**(4b•5)⊂TI-1**	3×10^{8} M^{-2} [a]	$3.8 \pm 0.3 \times 10^{-3}$ [c] s^{-1}	$1.1 \pm 0.7 \times 10^{5}$ [d] M	n.a
**6a⊂TI-1**	3×10^{5} M^{-1} [b]	n.a.	n.a	3×10^{-3} [e] M
**6b⊂TI-1**	$> 10^{7}$ M^{-1} [b]	n.a	n.a.	> 0.1 [e] M

[a] Determined by iterative manual refinement of the theoretical speciation profiles produced using HySS2009 software to the experimentally measured concentration ratio of the different species detected in the ^{1}H NMR spectra of the titrations of **TI-1**. We used a binding model that accounts for all possible binding equilibria between substrates **4** and **5** and **TI-1** (1:1 and 2:1 homo- and hetero-complexes). A single geometric isomer was considered for the 1:1 and 2:1 heterocomplexes; [b] Determined from competitive ^{1}H NMR experiments using monotopic guest **5** as reference; [c] Determined from the fit of the full-time-course ^{1}H NMR kinetic data of the cycloaddition reaction between **5** and **4a** or **4b** in the presence of equimolar amounts of **TI-1** using the kinetic model described in Figure 5 and COPASI software; [d] $EM_{kin} = k_{intra}/k_{bulk}$. k_{bulk} was taken from reference 22.[e] EM_{thermo}= K(**6⊂TI-1**)/K(**5₂⊂TI-1**). K(**5₂⊂TI-1**) = 8.1×10^{7} M^{-2}. n.a. not applicable

While preparing the table above, we realized there was an error in the EM_{kin} value for **6b**. We have now corrected the value throughout the manuscript.

Reviewer #3

1. Please remove the period following “Huisgen cycloadditions” in the title

Answer) We thank the reviewer for noticing this typographical issue. We have removed the final period from the title in the revised manuscript.

2. The border artifacts in Figures 6a and 6b should be removed to improve the clarity of the figures.

Answer) In our version, there are no border artifacts. These might be caused by the PDF generation during the submission process. In any case, we have regenerated the figure to ensure the best quality.

Reviewer #4

No comments provided.

Reviewer #5

We thank the reviewer for the very positive assessment of our manuscript and for recognizing the relevance of our work.

REVIEWERS' COMMENTS

Reviewer #1 (Remarks to the Author):

The authors have revised the manuscript accordingly and satisfactorily; thus this reviewer support the publication of this fine work in Nature Communications in its current, revised form.

Answer) We thank the reviewer for the very positive assessment of our work.

Reviewer #2 (Remarks to the Author):

The revised report by Ballester and co-workers on the tuning of the acceleration/inhibition of a catalytic Huisgen reaction in the confined space of a bis-calix[4]pyrrole cage results in a more accessible manuscript for the general audience of Nature Commun. This referee particularly appreciates the better positioning of the present work relatively to other systems of the literature notably regarding the enthalpic/entropic nature of the observed accelerations and the improved overall reading of the paper. Please precise the chemical nature of the R, R1 and R2 groups in Scheme 1 prior to publication (this can be added in proof, no need for further referring).

Answer) We thank the reviewer for this suggestion. We have defined the nature of R, R1, and R2 in Scheme 1, which is labeled Figure 1 in the revised version of the manuscript.

Reviewer #3

No comments provided.

Reviewer #4

No comments provided.
